# Palladium-catalyzed amidocarbonylation of thioethers: access to α-amide-substituted thioether derivatives

**DOI:** 10.1039/d5sc06696d

**Published:** 2025-10-30

**Authors:** Xudong Mao, Le-Cheng Wang, Xiao-Feng Wu

**Affiliations:** a Dalian National Laboratory for Clean Energy, Dalian Institute of Chemical Physics, Chinese Academy of Sciences Dalian China Xiao-Feng.Wu@catalysis.de xwu2020@dicp.ac.cn; b Leibniz-Institut für Katalyse e.V. Rostock Germany

## Abstract

Thioethers play a crucial role as structural components in a wide range of natural products, pharmaceuticals, and industrial materials, influencing their chemical characteristics and biological functions. Herein, we report a palladium-catalyzed aminocarbonylation reaction of thioethers with amines as nucleophiles. This palladium-catalyzed carbonylation reaction features a wide substrate scope and functional group tolerance, enabling the efficient C(sp^3^)–H aminocarbonylation of thioethers to afford the corresponding amides in good yields.

## Abstract 2

A palladium-catalyzed aminocarbonylation reaction of thioethers with amines as nucleophiles has been developed.

## Graphical abstract

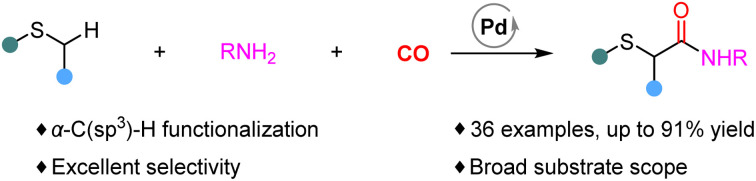

## Introduction

Thioethers are important structural skeletons that are widely found in natural products, pharmaceutical drugs, and industrial materials.^1^ In addition, they serve as powerful transition-metal ligands^2^ and organocatalysts^3^ in organic synthesis, and a promising class of cathode materials for rechargeable metal batteries.^4^ Furthermore, sulfoxides and sulfones, both important molecular scaffolds, can be synthesized from thioethers through straightforward oxidation.^5^ In the synthesis, metal- and organocatalyzed C–S bond cleavage reactions provide an efficient approach for constructing C–C and C–X bonds, with thioethers serving as common substrates in such transformations.^6^ Meanwhile, thioether compounds can serve as directing groups, promoting remote C–H bond activation and functionalization *via* coordination between the sulfur atom and transition metals.^7^ The unique and versatile nature of sulfide derivatives has consistently driven the development of innovative and efficient synthetic approaches for their construction and derivatization.

**Scheme 1:**
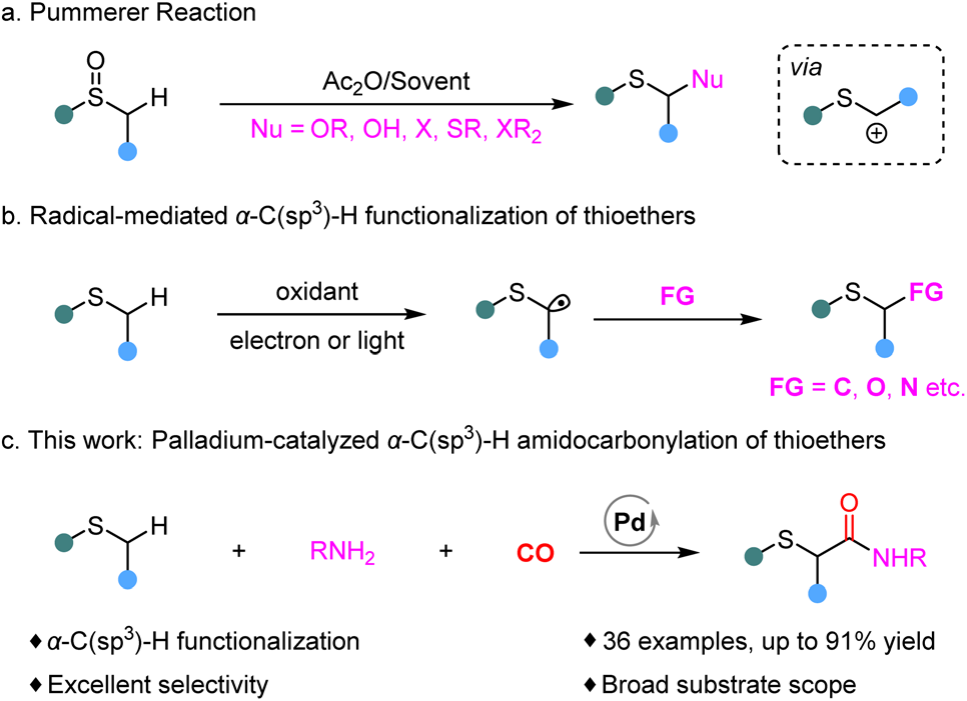
α-C(sp^3^)–H functionalization of thioethers.

C–H functionalization has emerged as one of the most efficient and atom-economical strategies among the diverse synthetic approaches. In the last century, the Pummerer reaction was discovered and has since been widely recognized as a powerful synthetic approach for the functionalization of the α-C(sp^3^)–H bond in sulfides (Scheme 1a).^8^ Since then, numerous methods for C–H bond functionalization of thioethers have been continuously reported,^9^ enabling the construction of C–C and C–X through radical intermediates or transition-metal catalysis (Scheme 1b).

Carbon monoxide, as a common C1 source, has been widely employed in industrial production and organic synthesis. Over the past decades, significant advances have been achieved in this field using Rh, Pd, Ru, Cu or Co catalysts.^10^ Among various carbonylation strategies, transition metal-catalyzed C(sp^3^)–H carbonylation has emerged as an efficient, atom-economical, and operationally simple strategy for the synthesis of carbonyl compounds.^11^ However, to our knowledge, α-C(sp^3^)–H carbonylation of thioethers has not yet been reported. Nevertheless, incorporating CO into this system presents several challenges: (1) both carbon monoxide and sulfur can strongly coordinate with metal catalysts and poison them; (2) the oxidation states of sulfur are sensitive to the oxidizing system and (3) undesired regioselectivity may occur during transformations. Following our previous reports and continuing interest in C(sp^3^)–H carbonylation,^12^ we herein report a palladium-catalyzed α-C(sp^3^)–H carbonylation of thioethers using amines as nucleophiles, affording a series of α-aminocarbonylated thioether derivatives with excellent selectivity and good functional group tolerance (Scheme 1c).

## Results and discussion

Inspired by our previous efforts and past work on α-C(sp^3^)–H functionalization,^13^ we chose tetrahydrothiophene (1a) and aniline (2a) as model substrates to explore the proposed reaction (Table 1). Through systematic optimization, we found that using PdCl_2_ as the palladium catalyst, xantphos as the ligand, and DTBP as the oxidant, under 60 bar CO pressure at 120 °C afforded the desired α-C(sp^3^)–H aminocarbonylation product 3a in 85% isolated yield with excellent regioselectivity (Table 1, entry 1). Control experiments indicate that the palladium catalyst, ligand and DTBP were all essential for this reaction (Table 1, entries 2–4). Subsequent evaluation of the palladium catalyst demonstrated that the catalyst significantly influenced the reaction efficiency, providing substantially lower yields when Pd(acac)_2_, PdI_2_ or Pd(OAc)_2_ was used (Table 1, entry 5 and 7), and the desired product was not detected when CoCl_2_, NiCl_2_ or CuCl_2_ was used as the metal pre-catalyst (Table 1, entry 8). In addition, we tried different ligands, and both monophosphine (PPh_3_) and diphosphine ligands (DPEphos and DPPF), which in lower yields (Table 1, entry 9 and 11). Besides this, all tested peroxides (TBHP, BPO and TBPB) failed to afford the desired product, indicating the high sensitivity of the reaction to the oxidation system (Table 1, entry 12 and 14). Notably, the yield of 3a decreased significantly when we tested the reaction with a lower loading of palladium (5 mol%). It is worth mentioning that no by-product from β-position C–H bond activation of tetrahydrothiophene was observed during the optimization process.

**Table 1:** Optimization of reaction conditions^a^

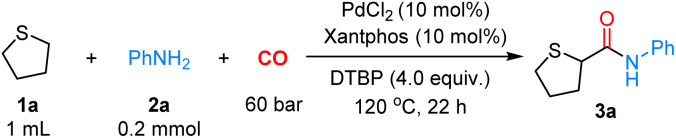		
Entry	Variation in standard conditions	Yield (%)^b^
1	None	77 (85)^c^
2	No PdCl_2_	0
3	No xantphos	0
4	No DTBP	0
5	Pd(acac)_2_ instead of PdCl_2_	18
6	PdI_2_ instead of PdCl_2_	19
7	Pd(OAc)_2_ instead of PdCl_2_	19
8	CoCl_2_, NiCl_2_ or CuCl_2_ instead of PdCl_2_	0
9	DPEphos instead of xantphos	55
10	DPPF instead of xantphos	20
11	PPh_3_ instead of xantphos	62
12	TBHP instead of DTBP	0
13	BPO instead of DTBP	0
14	TBPB instead of DTBP	0

a Reaction conditions: 1a (1.0 mL), 2a (0.2 mmol), PdCl_2_ (10 mol%), xantphos (10 mol%), DTBP (4.0 equiv.), 60 bar CO, 120 °C, 22 h.

b Yields were determined by GC analysis using hexadecane as the internal standard.

c Isolated yield.

**Scheme 2:**
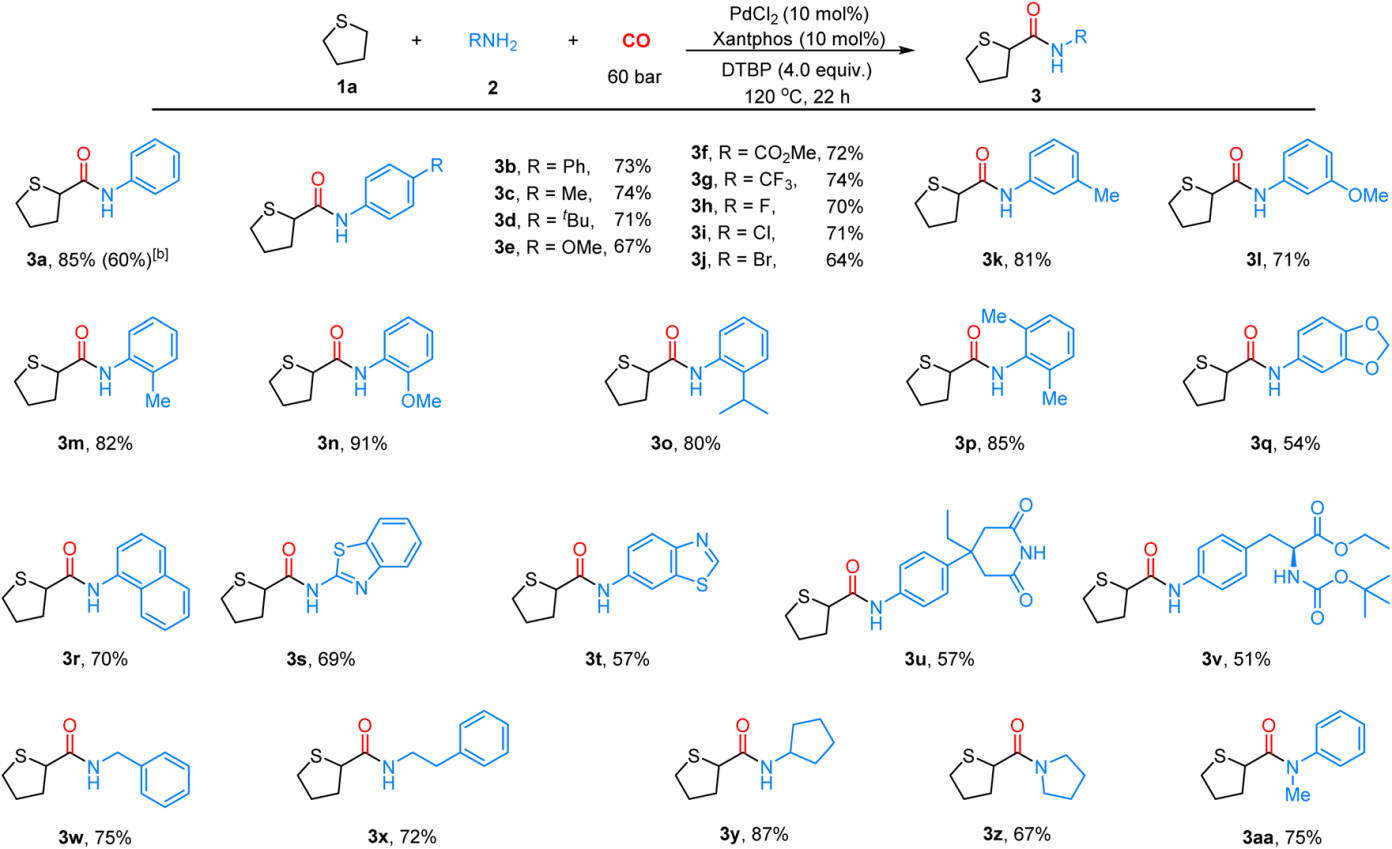
Scope of amines. ^*a*^Unless noted otherwise, reaction conditions were as follows: ^*a*^reaction conditions: 1 (1.0 mL), 2 (0.2 mmol, 1.0 equiv.), PdCl_2_ (10 mol%), xantphos (10 mol%), DTBP (4.0 equiv.), CO (60 bar), 120 °C, 22 h. ^*b*^2.0 mmol level: 1 (10.0 mL), 2 (2.0 mmol, 1.0 equiv.), PdCl_2_ (10 mol%), xantphos (10 mol%), DTBP (4.0 equiv.), CO (60 bar), 120 °C, 22 h.

With the optimized conditions in hand, we next explored the substrate scope of this α-C(sp^3^)–H aminocarbonylation. As shown in Scheme 2, a series of amines were used as nucleophilic reagents in this reaction. First, various anilines bearing an electron-donating or electron-withdrawing group were compatible with the reaction, affording the desired products 3a–3j in moderate to good yields. Variations in the substituent position on aniline, including *ortho*-isopropyl (3o) and *ortho*-dimethyl (3p) groups, had minimal influence on the reaction efficiency, affording yields of 80% and 85%, respectively. In addition, heterocyclic amines were well-tolerated, affording the corresponding products 3q–3t in 54–70% yields. Moreover, we tried to extend the reaction to some complex natural products and pharmaceutical derivatives, and aminoglutethimide 3u and amino acid derivatives 3v were obtained in 57% and 71% yields, respectively. Then, we tested several alkylamines as nucleophiles, giving products 3w–3y in 72–87% yields, and 2° amines were also applicable to this reaction, giving products 3z and 3aa in moderate yields. To further probe the synthetic utility of this reaction, the model reaction was conducted on a 2.0 mmol gram scale, leading to product 3a in 60% yield.

**Scheme 3:**
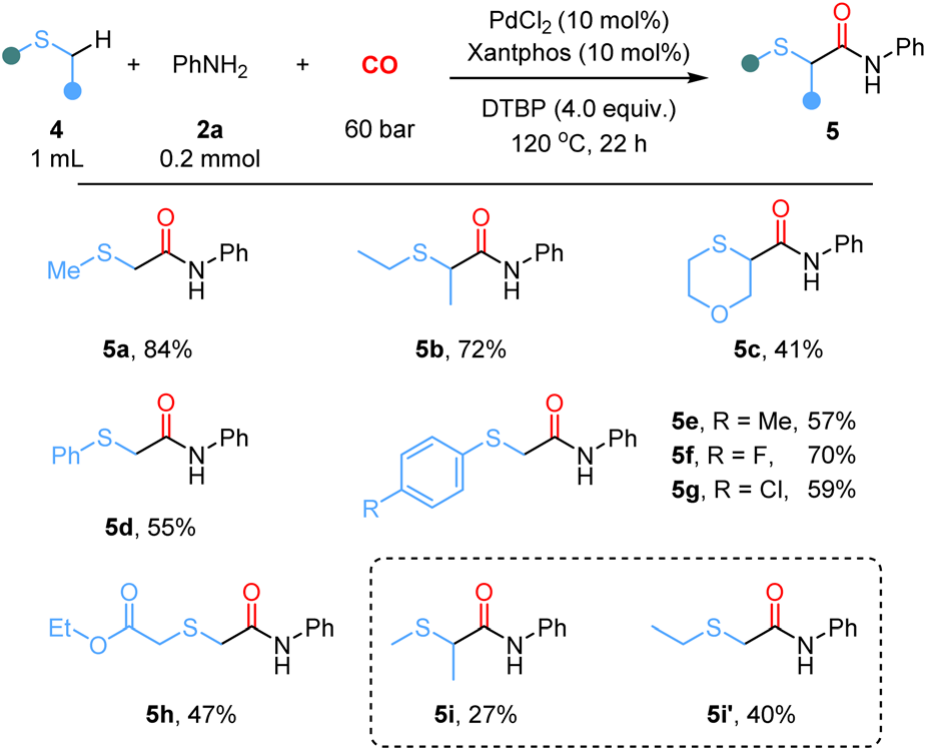
Scope for thioethers. ^*a*^Reaction conditions: 4 (1.0 mL), 2a (0.2 mmol, 1.0 equiv.), PdCl_2_ (10 mol%), xantphos (10 mol%), DTBP (4.0 equiv.), CO (60 bar), 120 °C, 22 h.

Next, the substrate scope of thioethers was investigated in the α-C(sp^3^)–H aminocarbonylation of aniline 2a (Scheme 3). The thioether was used as both the reagent and solvent in the following reaction. Initially, symmetrical linear and cyclic sulfides were subjected to this reaction, affording the corresponding carbonylation products (5a–5c) in moderate to good yields. Subsequently, a series of aryl methyl sulfides were examined, and the reaction consistently afforded the desired products in 57–70% yields regardless of substituent variations (5d–5g). Notably, when 4-methylphenyl methyl sulfide was used, the reaction selectively targeted the α-C(sp^3^)–H bond of the thioethers, affording product 5e. Here the slightly decreased yield was due to the oxidation of the methyl group on the aromatic ring. Moreover, the reaction of asymmetric thioethers gave product 5h in 47% yield. In the case of methylthioethane, a mixture of products was obtained, with a combined yield of 67% (5i and **5i′**).

**Scheme 4:**
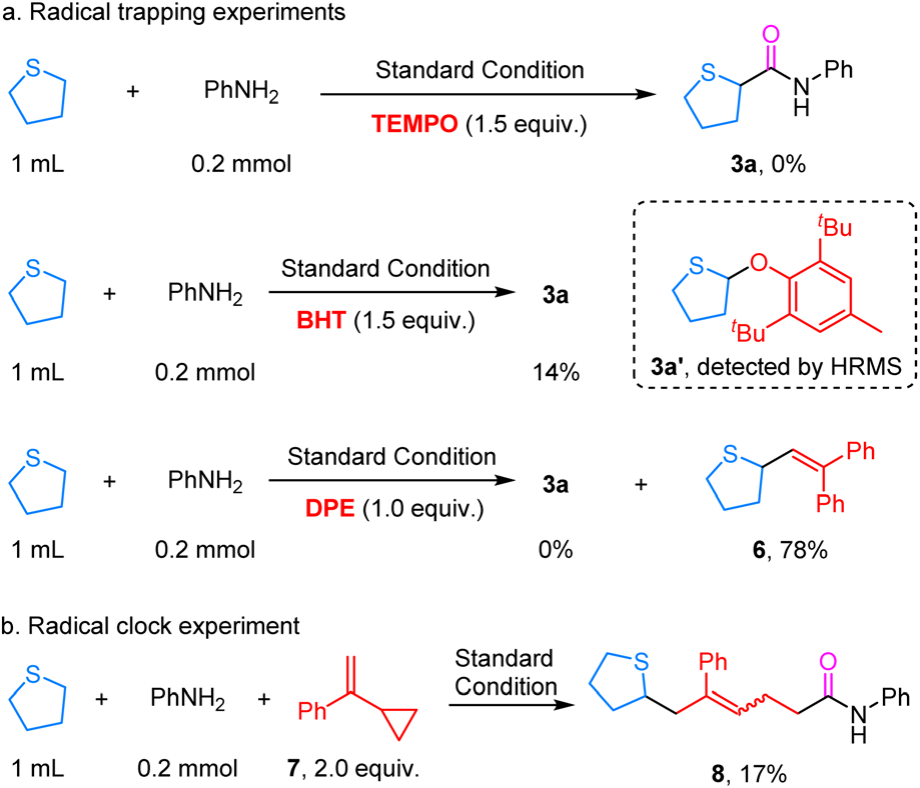
Mechanistic experiments.

To gain some insight into the reaction mechanism, a series of mechanistic studies were conducted (Scheme 4). First, we added some radical trapping reagents; with the addition of 2,2,6,6-tetramethylpiperidine-1-oxy (TEMPO), the reaction was completely quenched. When BHT (butylated hydroxytoluene) was added to this reaction, the yield was significantly suppressed, affording the desired product 3a in only 14% yield. Meanwhile, high resolution mass spectrometry (HRMS) detected a signal corresponding to the BHT-trapped intermediate **3a′**, providing preliminary evidence for the possible involvement of a tetrahydrothiophene radical pathway in the reaction. Subsequently, the addition of TBH as a radical scavenger afforded the coupling product 5 in 78% yield. Subsequently, using (2-cyclopropylallyl)benzene 6 under standard conditions afforded the ring-opened product 7 in 78% yield, further supporting the involvement of a radical pathway in the reaction (Scheme 4b).

**Scheme 5:**
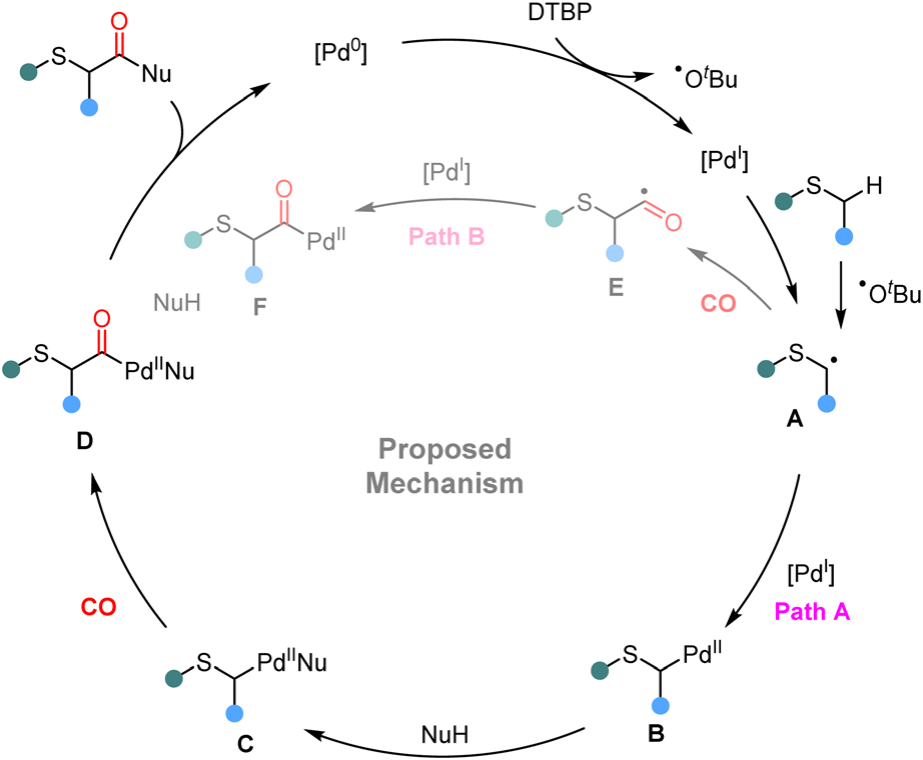
Proposed mechanism.

Based on mechanistic studies and literature reports,^14^ a plausible mechanistic pathway is proposed as follows (Scheme 5). Initially, Pd(0) is oxidized by DTBP to generate Pd(i) species, accompanied by the formation of a *tert*-butoxy radical. The generated *tert*-butoxy radical undergoes a hydrogen atom transfer (HAT) process with the thioether, generating the corresponding alkyl radical intermediate A. The resulting radical A is captured by the Pd(i) species to form a Pd(ii) intermediate B. After ligand exchange, intermediate C is generated, which then undergoes CO insertion to afford acyl-Pd(ii) species D. Finally, reductive elimination of Pd(ii) species D affords the desired α-carbonylated product and regenerates the Pd(0) catalyst, which is then ready for the next catalytic cycle. Alternatively, another mechanistic scenario involves the direct carbonylation of the alkyl radical with CO to generate an acyl radical intermediate E. This intermediate can then undergo radical addition to the Pd(i) center to form the acyl-Pd(ii) species F. Subsequent ligand exchange and reductive elimination deliver the final product. However, additional work is required to further clarify the mechanism.

## Conclusions

In summary, we have developed a new α-C(sp^3^)–H carbonylation reaction of thioethers. Given the significant synthetic value and pharmaceutical relevance of thioether-containing compounds, along with the inherent challenges of this transformation, various metal catalysts and oxidants were systematically screened. Ultimately, a palladium catalyst in combination with DTBP as the oxidant enabled the efficient synthesis of a series of sulfur-containing amide derivatives with excellent regioselectivity and good functional group tolerance. Mechanistic studies suggest that the carbonylation proceeds *via* a HAT process, generating an alkyl radical intermediate from the thioether, which subsequently undergoes palladium-catalyzed carbonylation.

## Author contributions

X. M. and L. C. W. performed all the experiments and prepared the manuscript and SI. X. F. W. conceived the project, supervised the research, and revised the manuscript.

## Conflicts of interest

There are no conflicts to declare.

## Supplementary Material

SC-016-D5SC06696D-s001

## Data Availability

The data supporting this article have been included as part of the supplementary information (SI). Supplementary information: general comments, general procedure, analytic data, and NMR spectra. See DOI: https://doi.org/10.1039/d5sc06696d.
