# Supplementary material for: Palladium-catalyzed amidocarbonylation of thioethers: access to α-amide-substituted thioether derivatives
Source: Chem Sci. 2025 Oct 30;16(48):23310–4. doi: 10.1039/d5sc06696d (PMC12591059; doi:10.1039/d5sc06696d)

Supplementary Information (SI) for Chemical Science.
This journal is © The Royal Society of Chemistry 2025

Supporting Information

Contents

1. General Information 1
2. Optimization of Reaction Conditions 2
3. Experimental Procedures and Product Characterization 4
4. Mechanistic Studies 5
5. Spectroscopic Data of Products 7
6. Reference 21
7. The NMR Spectrum 22

1. General Information

General. Unless otherwise noted, all reactions were carried out under carbon monoxide or nitrogen atmosphere. All reagents were from commercial sources and used as received without further purification. All solvents were dried by standard techniques and distilled prior to use. Column chromatography was performed on silica gel (200-300 meshes) using petroleum ether (bp. 60~90 °C), dichloromethane and ethyl acetate as eluent. All NMR spectra were recorded at ambient temperature using Bruker Avance III 400 MHz NMR (^{1}H, 400 MHz; ^{13}C {^{1}H}, 101 MHz, ^{19}F 376 MHz), Bruker AVANCE III HD 700 MHz NMR spectrometers (^{1}H, 700 MHz; ^{13}C{^{1}H}, 176 MHz). ^{1}H NMR chemical shifts are reported relative to TMS and were referenced via residual proton resonances of the corresponding deuterated solvent ($CDCl_3$: 7.26 ppm; d_6-DMSO: 2.50 ppm) whereas ^{13}C{^{1}H} NMR spectra are reported relative to TMS via the carbon signals of the deuterated solvent ($CDCl_3$: 77.0 ppm; d_6-DMSO: 39.5 ppm). Data for ^{1}H are reported as follows: chemical shift (δ ppm), multiplicity (s = singlet, d = doublet, t = triplet, q = quartet, dd (doublet of doublets), dt (doublet of triplets), m = multiplet), coupling constant (Hz), and integration. All ^{13}C NMR spectra were broad band ^{1}H decoupled. All reactions were monitored by GC-FID or NMR analysis. HRMS data was obtained with Micromass HPLC-Q-TOF mass spectrometer (ESI-TOF) or Agilent 6540 Accurate-MS spectrometer (Q-TOF).

Caution!!! (The high toxicity of carbon monoxide, all the reactions should be performed in an autoclave. The laboratory should be well-equipped with a CO detector and alarm system.)

2. Optimization of Reaction Conditions

Table S1. Optimization of metals

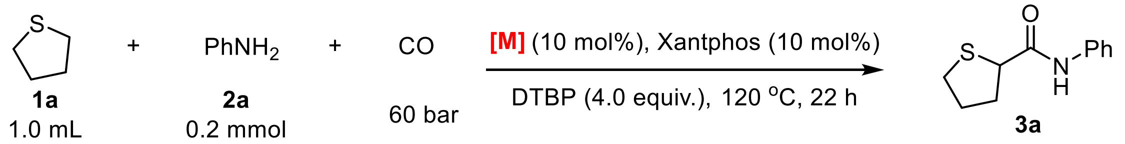

Entry	[M]	Yield[a]
1	$PdCl_2$	77% (85%)[b]
2	$Pd(OAc)_2$	19%
3	$Pd(acac)_2$	18%
4	PdI_2	19%
5	$Pd(dba)_2$	13%
6	$Pd(PPh_3)_4$	14%
7	$FeCl_2$	ND
8	$FeCl_3$	ND
9	$CoCl_2$	ND
10	$CuCl_2$	ND
11	$NiCl_2$	ND

[a]Determined by GC using hexadecane as an internal standard. [b]Isolated yield

Table S2. Optimization of ligands

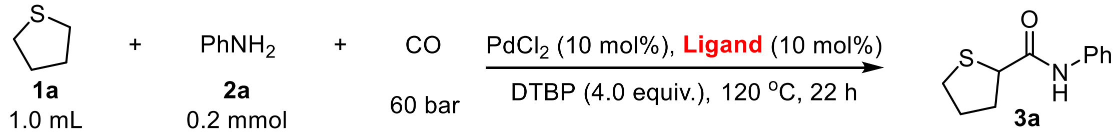

Entry	Ligands	Yield[a]
1	PPh_3	62%
2	DPPE	18%
3	DPPF	20%
4	DPE-Phos	55%
5	BINAP	9%
6	Bpy	19%
7	1,10-Phen	14%

[a]Determined by GC using hexadecane as an internal standard.

Table S3. Optimization of peroxides

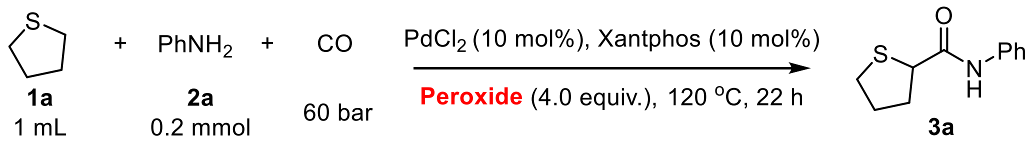

Entry	**Peroxide**	Yield[a]
1	BPO	ND
2	TBHP	ND
3	H_2O_2	ND
4	$K_2S_2O_8$	ND
5	*m*-CPBA	ND
6	TBPB	ND

[a]Determined by GC using hexadecane as an internal standard.

3. Experimental Procedures and Product Characterization

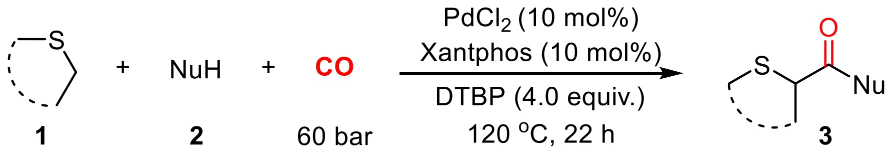

General Procedure: Under an argon atmosphere, a 4 mL screw-cap vial was charged with equipped with a magnetic stir bar was charged with $PdCl_2$ (3.5 mg, 0.02 mmol, 10 mol%), Xantphos (11.6 mg, 0.02 mmol, 10 mol%), **1** sulfides (1 mL, 0.2 M), **2** amines (1 equiv., 0.2 mmol) and DTBP (4.0 equiv.) were added nitrogen atmosphere, the vial was moved to an alloy plate and put into a Parr 4560 series autoclave (300 mL) under an argon atmosphere. At room temperature, the autoclave was flushed with CO three times and charged with 60 bar of CO. The autoclave was placed on a heating plate equipped with a magnetic stirrer and an aluminum block. The reaction mixture was heated to 120 °C for 22 h. After the reaction was complete, the autoclave was cooled down with ice water to room temperature and the pressure was released carefully. After cooling to room temperature, the reaction mixture was directly purified by column chromatography on silica gel using petroleum ether and ethyl acetate to afford the corresponding product.

4. Mechanistic Studies

Radical trapping experiment

Following the standard procedure of the model reaction, when 1.5 equiv. of radical inhibitor 2,2,6,6-tetramethylpiperidine-1-oxy (TEMPO) was added to the reaction mixture, the formation of the desired product **3a** was completely inhibited. When 2,6-di-tert-butyl-4-methylphenol (BHT) was added to the reaction mixture, the formation of the desired product **3a** was partly inhibited. The adducts of tetrahydrothiophene radical by BHT were confirmed by high-resolution mass spectrometry (HRMS).

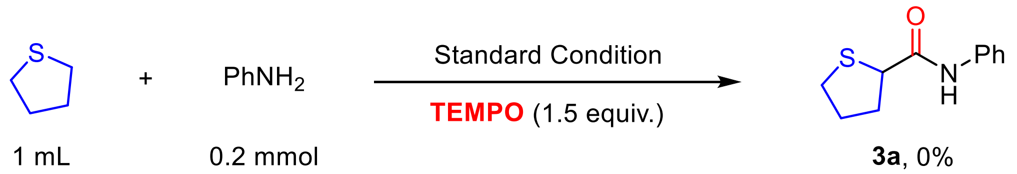

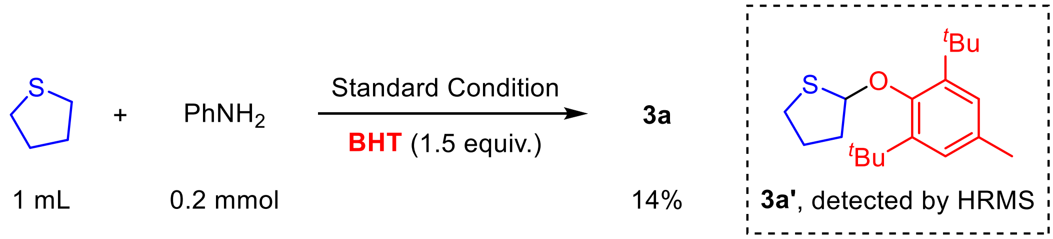

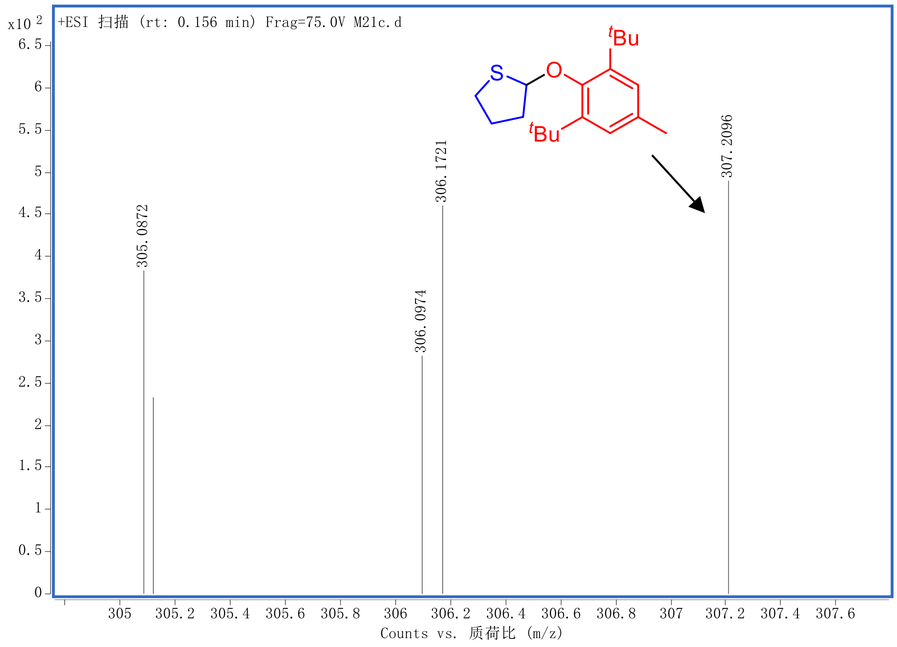

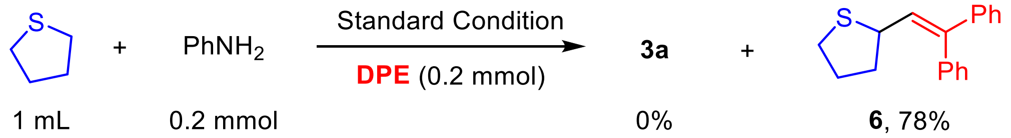

Following the standard procedure of the model reaction, when 0.2 mmol of radical inhibitor 1,1-Diphenylethylene (DPE) was added to the reaction mixture, the formation of the desired

product **3a** was completely inhibited, and the coupling product **6** was obtained in 78% yield.

2-(2,2-Diphenylvinyl)tetrahydrothiophene (**6**)

41.5 mg, 78% yield, a colorless oil, eluent: petroleum ether/ethyl acetate = 20:1.

^{1}H NMR (400 MHz, CDCl$_3$) δ 7.40-7.35 (m, 2H), 7.34-7.29 (m, 1H), 7.29-7.16 (m, 7H), 6.04 (d, *J* = 10.3 Hz, 1H), 4.10-3.90 (m, 1H), 3.07-2.96 (m, 1H), 2.91-2.82 (m, 1H), 2.25-2.07 (m, 2H), 1.92-1.69 (m, 2H).

^{13}C NMR (101 MHz, CDCl$_3$) δ 142.0, 141.6, 139.4, 130.6, 130.0, 129.9, 128.2, 128.1, 127.4, 127.2, 47.7, 38.6, 33.4, 31.2.

Radical clock experiments

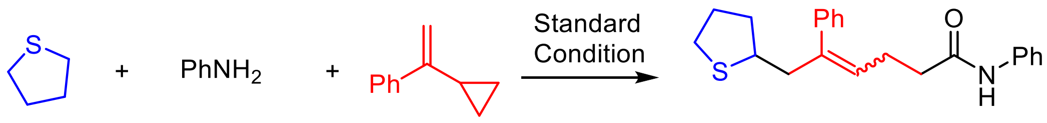

The radical clock reaction was performed according to the general procedure. Following the standard procedure of the model reaction, when 1.0 equiv. (1-cyclopropylvinyl)benzene was added to the reaction mixture. The ring-opened product **8** was obtained as an E/Z mixture of alkenes in 17% yield.

***N*,5-Diphenyl-6-(tetrahydrothiophen-2-yl)hex-4-enamide** (**8**)

11.5 mg, 17% yield, a colorless oil, eluent: petroleum ether/ethyl acetate = 100:1.

^{1}H NMR (700 MHz, CDCl$_3$) δ 7.53 (d, *J* = 8.0 Hz, 1.8H), 7.48 (d, *J* = 8.0 Hz, 0.8H), 7.35-7.28 (m, 4.8H), 7.27-7.21 (m, 1.7H), 7.13 (s, 0.8H), 7.12-7.07 (m, 1.1H), 5.67 (t, *J* = 7.4 Hz, 0.6H), 5.57 (t, *J* = 6.2 Hz, 0.4H), 3.36-3.30 (m, 0.6H), 3.29-3.19 (m, 0.4H), 2.90-2.81 (m, 2.3H), 2.81-2.76 (m, 1.1H), 2.72-2.65 (m, 1.6H), 2.56-2.49 (m, 1.7H), 2.39-2.33 (m, 1.7H), 2.07-2.01 (m, 1.2H), 1.98-1.92 (m, 1H), 1.84-1.77 (m, 1H), 1.59-1.52 (m, 1H).

^{13}C NMR (175 MHz, CDCl$_3$) δ 170.7, 170.6, 142.2, 141.5, 140.5, 139.8, 137.9, 137.9, 128.94, 128.87, 128.3, 128.2, 127.01, 126.95, 126.7, 126.6, 124.2, 124.1, 119.80, 119.77, 47.6, 47.3, 47.2, 38.0, 37.6, 37.2, 36.9, 36.7, 32.3, 32.2, 30.2, 24.9, 24.8.

5. Spectroscopic Data of Products

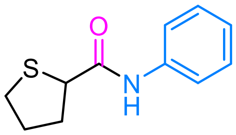

***N*-Phenyltetrahydrothiophene-2-carboxamide (3a)**[1] Prepared according to the general procedure from **1a** (1 mL, 0.2 M) and **2a** (0.20 mmol) purified by column chromatography on silica gel with petroleum ether/ethyl acetate (10:1) to provide the title compound **3a** as a white solid (35.3 mg, 85 % yield); m.p. 110.5-111.3 °C; ^{1}H NMR (400 MHz, $CDCl_3$) δ 8.90 (s, 1H), 7.56 (d, *J* = 7.6 Hz, 2H), 7.32 (t, *J* = 7.5 Hz, 2H), 7.19-7.06 (m, 1H), 4.16-3.91 (m, 1H), 3.18-3.00 (m, 1H), 3.00-2.87 (m, 1H), 2.53-2.35 (m, 1H), 2.38-2.16 (m, 1H), 2.16-2.02 (m, 1H), 2.01-1.86 (m, 1H); ^{13}C NMR (101 MHz, $CDCl_3$) δ 170.2, 137.5, 129.0, 124.5, 119.7, 52.4, 36.2, 33.3, 30.0; HRMS (ESI) Calcd for $C_{11}H_{14}NOS$ $[M + H]^+$ 208.0791, found 208.0799.

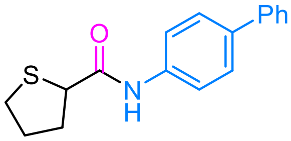

***N*-([1,1'-Biphenyl]-4-yl)tetrahydrothiophene-2-carboxamide (3b)** Prepared according to the general procedure from **1a** (1 mL, 0.2 M) and **2b** (0.20 mmol) purified by column chromatography on silica gel with petroleum ether/ethyl acetate (10:1) to provide the title compound **3b** as a light yellow solid (41.3 mg, 73% yield); m.p. 151.3-153.3 °C; ^{1}H NMR (400 MHz, $CDCl_3$) δ 8.96 (s, 1H), 7.64 (d, *J* = 8.5 Hz, 2H), 7.59-7.52 (m, 4H), 7.41 (t, *J* = 7.6 Hz, 2H), 7.31 (t, *J* = 7.3 Hz, 1H), 4.06 (dd, *J* = 7.5, 4.4 Hz, 1H), 3.12-2.89 (m, 2H), 2.50-2.40 (m, 1H), 2.30-2.17 (m, 1H), 2.15-2.03 (m, 1H), 2.01-1.89 (m, 1H); ^{13}C NMR (101 MHz, $CDCl_3$) δ 170.2, 140.4, 137.3, 136.8, 128.7, 127.5, 127.1, 126.8, 119.9, 52.3, 36.1, 33.3, 29.9; HRMS (ESI) Calcd for $C_{17}H_{18}NOS$ $[M + H]^+$ 284.1104, found 284.1110.

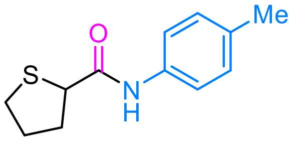

***N*-(*p*-Tolyl)tetrahydrothiophene-2-carboxamide (3c)** Prepared according to the general procedure from **1a** (1 mL, 0.2 M) and **2c** (0.20 mmol) purified by column chromatography on silica gel with petroleum ether/ethyl acetate (10:1) to provide the title compound **3c** as a light yellow solid (32.7 mg, 74% yield); m.p. 90.9-92.7; ^{1}H NMR (400 MHz, $CDCl_3$) δ 8.83 (s, 1H),

7.43 (d, J = 8.4 Hz, 2H), 7.12 (d, J = 8.3 Hz, 2H), 4.04 (dd, J = 7.5, 4.4 Hz, 1H), 3.11-2.89 (m, 2H), 2.49-2.39 (m, 1H), 2.31 (s, 3H), 2.27-2.16 (m, 1H), 2.13-2.05 (m, 1H), 2.00-1.87 (m, 1H); ^{13}C NMR (101 MHz, $CDCl_3$) δ 170.0, 134.9, 134.1, 129.4, 119.7, 52.3, 36.1, 33.2, 29.9, 20.8; HRMS (ESI) Calcd for $C_{12}H_{16}NOS$ $[M + H]^+$ 222.0947, found 222.0956.

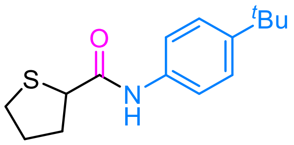

***N*-(4-(*tert*-Butyl)phenyl)tetrahydrothiophene-2-carboxamide (3d)** Prepared according to the general procedure from **1a** (1 mL, 0.2 M) and **2d** (0.20 mmol) purified by column chromatography on silica gel with petroleum ether/ethyl acetate (10:1) to provide the title compound **3d** as a white solid (37.4 mg, 71% yield); m.p. 101.4-103.3 °C; ^{1}H NMR (400 MHz, $CDCl_3$) δ 8.85 (s, 1H), 7.51-7.44 (m, 2H), 7.38-7.31 (m, 2H), 4.05 (dd, J = 7.6, 4.4 Hz, 1H), 3.10-3.03 (m, 1H), 2.97-2.89 (m, 1H), 2.49-2.37 (m, 1H), 2.27-2.18 (m, 1H), 2.14-2.04 (m, 1H), 1.98-1.87 (m, 1H), 1.30 (s, 9H); ^{13}C NMR (101 MHz, $CDCl_3$) δ 170.0, 147.5, 134.9, 125.7, 119.4, 52.3, 36.2, 34.3, 33.2, 31.3, 29.9; HRMS (ESI) Calcd for $C_{15}H_{22}NOS$ $[M + H]^+$ 264.1417, found 264.1425.

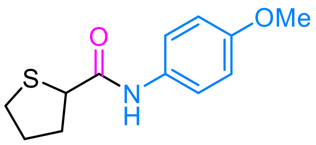

4-(4-(Trimethylsilyl)phenethyl)benzonitrile (3e) Prepared according to the general procedure from **1a** (1 mL, 0.2 M) and **2e** (0.20 mmol) purified by column chromatography on silica gel with petroleum ether/ethyl acetate (10:1) to provide the title compound **3e** as a white solid (31.8 mg, 67% yield); m.p. 99.4-101.1 °C; ^{1}H NMR (400 MHz, $CDCl_3$) δ 8.79 (s, 1H), 7.53-7.37 (m, 2H), 6.92-6.78 (m, 2H), 4.05 (dd, J = 7.6, 4.4 Hz, 1H), 3.79 (s, 3H), 3.12-3.02 (m, 1H), 2.99-2.90 (m, 1H), 2.50-2.39 (m, 1H), 2.30-2.18 (m, 1H), 2.16-2.05 (m, 1H), 2.00-1.87 (m, 1H); ^{13}C NMR (101 MHz, $CDCl_3$) δ 169.9, 156.5, 130.7, 121.4, 114.1, 55.5, 52.3, 36.3, 33.3, 29.9; HRMS (ESI) Calcd for $C_{12}H_{16}NO_2S$ $[M + H]^+$ 238.0896, found 238.0904.

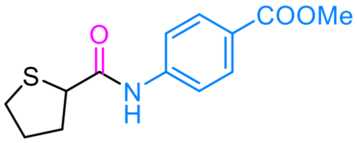

Methyl 4-(tetrahydrothiophene-2-carboxamido)benzoate (3f) Prepared according to the general procedure from **1a** (1 mL, 0.2 M) and **2f** (0.20 mmol) purified by column chromatography on silica gel with petroleum ether/ethyl acetate (5:1) to provide the title compound **3f** as a dark red solid (38.2 mg, 72% yield); m.p. 92.4-94.0 °C; ^{1}H NMR (400 MHz, $CDCl_3$) δ 9.06 (s, 1H), 8.02 (d, J = 8.8 Hz, 2H), 7.66 (d, J = 8.7 Hz, 2H), 4.07 (dd, J = 7.6, 4.4 Hz, 1H), 3.90 (s, 3H), 3.13-3.05 (m, 1H), 3.01-2.92 (m, 1H), 2.50-2.41 (m, 1H), 2.31-2.22 (m, 1H), 2.17-2.09 (m, 1H), 1.99-1.89 (m, 1H); ^{13}C NMR (101 MHz, $CDCl_3$) δ 170.5, 166.5, 141.6, 130.7, 125.8, 118.7, 52.3, 52.0, 36.1, 33.3, 30.0; HRMS (ESI) Calcd for $C_{13}H_{16}NO_3S$ $[M + H]^+$ 266.0845, found 266.0841.

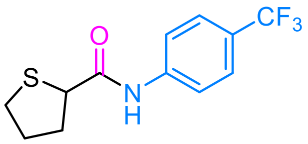

***N*-(4-(Trifluoromethyl)phenyl)tetrahydrothiophene-2-carboxamide (3g)** Prepared according to the general procedure from **1a** (1 mL, 0.2 M) and **2g** (0.20 mmol) purified by column chromatography on silica gel with petroleum ether/ethyl acetate (10:1) to provide the title compound **3g** as a white solid (40.7 mg, 74% yield); m.p. 93.7-95.0 °C; ^{1}H NMR (400 MHz, $CDCl_3$) δ 9.05 (s, 1H), 7.70 (d, J = 8.5 Hz, 2H), 7.59 (d, J = 8.5 Hz, 2H), 4.07 (dd, J = 7.6, 4.4 Hz, 1H), 3.14-3.05 (m, 1H), 3.02-2.92 (m, 1H), 2.50-2.39 (m, 1H), 2.32-2.21 (m, 1H), 2.18-2.08 (m, 1H), 2.00-1.88 (m, 1H); ^{13}C NMR (101 MHz, $CDCl_3$) δ 170.6, 140.5, 126.2 (q, J_{C-F} = 33 Hz), 126.3 (q, J_{C-F} = 4 Hz) 124.0 (q, J_{C-F} = 270 Hz), 119.2, 52.4, 36.2, 33.4, 30.0; ^{19}F NMR (376 MHz, $CDCl_3$) δ -62.14; HRMS (ESI) Calcd for $C_{12}H_{13}F_3NOS$ $[M + H]^+$ 276.0664, found 276.0660.

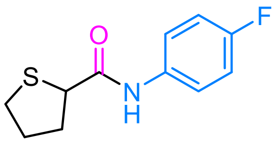

***N*-(4-Fluorophenyl)tetrahydrothiophene-2-carboxamide (3h)** Prepared according to the general procedure from **1a** (1 mL, 0.2 M) and **2h** (0.20 mmol) purified by column chromatography on silica gel with petroleum ether/ethyl acetate (10:1) to provide the title

compound **3h** as a yellow solid (31.5 mg, 70% yield); m.p. 71.3-73.5 °C; ^1H NMR (400 MHz, $CDCl_3$) δ 8.89 (s, 1H), 7.52 (dd, J = 9.0, 4.8 Hz, 2H), 7.02 (t, J = 8.6 Hz, 2H), 4.05 (dd, J = 7.6, 4.4 Hz, 1H), 3.13-3.03 (m, 1H), 2.99-2.89 (m, 1H), 2.49-2.37 (m, 1H), 2.30-2.17 (m, 1H), 2.17-2.04 (m, 1H), 2.01-1.86 (m, 1H); ^{13}C NMR (101 MHz, $CDCl_3$) δ 170.2, 159.1 (d, J_{C-F} = 243 Hz), 133.5 (d, J_{C-F} = 3 Hz), 121.4 (d, J_{C-F} = 8 Hz), 115.7 (d, J_{C-F} = 22 Hz), 52.2, 36.1, 33.3, 29.9; ^{19}F NMR (376 MHz, $CDCl_3$) δ -117.69; HRMS (ESI) Calcd for $C_{11}H_{13}FNOS$ $[M + H]^+$ 226.0696, found 226.0695.

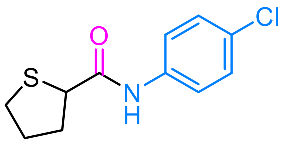

***N*-(4-Chlorophenyl)tetrahydrothiophene-2-carboxamide (3i)** Prepared according to the general procedure from **1a** (1 mL, 0.2 M) and **2i** (0.20 mmol) purified by column chromatography on silica gel with petroleum ether/ethyl acetate (10:1) to provide the title compound **3i** as a white solid (34.1 mg, 71% yield); m.p. 91.1-92.7 °C; ^1H NMR (400 MHz, $CDCl_3$) δ 8.90 (s, 1H), 7.58-7.47 (m, 2H), 7.33-7.26 (m, 2H), 4.05 (dd, J = 7.6, 4.3 Hz, 1H), 3.13-3.03 (m, 1H), 3.01-2.91 (m, 1H), 2.50-2.39 (m, 1H), 2.31-2.19 (m, 1H), 2.17-2.07 (m, 1H), 1.99-1.86 (m, 1H); ^{13}C NMR (101 MHz, $CDCl_3$) δ 170.2, 136.1, 129.5, 129.0, 120.9, 52.4, 36.2, 33.3, 29.9; HRMS (ESI) Calcd for $C_{11}H_{13}ClNOS$ $[M + H]^+$ 242.0401, found 242.0409.

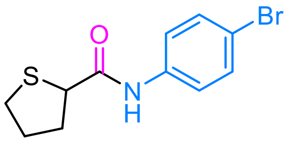

***N*-(4-Bromophenyl)tetrahydrothiophene-2-carboxamide (3j)** Prepared according to the general procedure from **1a** (1 mL, 0.2 M) and **2j** (0.20 mmol) purified by column chromatography on silica gel with petroleum ether/ethyl acetate (10:1) to provide the title compound **3j** as a white solid (36.6 mg, 64% yield); m.p. 96.6-98.9 °C; ^1H NMR (400 MHz, $CDCl_3$) δ 8.90 (s, 1H), 7.51-7.40 (m, 4H), 4.04 (dd, J = 7.6, 4.4 Hz, 1H), 3.13-3.02 (m, 1H), 3.01-2.89 (m, 1H), 2.50-2.36 (m, 1H), 2.33-2.19 (m, 1H), 2.19-2.04 (m, 1H), 2.01-1.85 (m, 1H); ^{13}C NMR (101 MHz, $CDCl_3$) δ 170.2, 136.6, 131.9, 121.2, 117.1, 52.4, 36.2, 33.3, 29.9; HRMS (ESI) Calcd for $C_{11}H_{13}BrNOS$ $[M + H]^+$ 285.9896, found 285.9891.

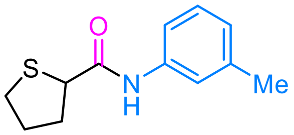

***N*-(*m*-Tolyl)tetrahydrothiophene-2-carboxamide (3k)** Prepared according to the general procedure from **1a** (1 mL, 0.2 M) and **2k** (0.20 mmol) purified by column chromatography on silica gel with petroleum ether/ethyl acetate (10:1) to provide the title compound **3k** as a yellow oil (35.9 mg, 81% yield); ^{1}H NMR (400 MHz, $CDCl_3$) δ 8.85 (s, 1H), 7.41 (s, 1H), 7.34 (d, *J* = 7.9 Hz, 1H), 7.20 (t, *J* = 7.8 Hz, 1H), 6.93 (d, *J* = 7.6 Hz, 1H), 4.04 (dd, *J* = 7.5, 4.4 Hz, 1H), 3.11-3.02 (m, 1H), 2.98-2.89 (m, 1H), 2.48-2.40 (m, 1H), 2.27-2.17 (m, 1H), 2.14-2.05 (m, 1H), 2.00-1.88 (m, 1H); ^{13}C NMR (101 MHz, $CDCl_3$) δ 170.1, 138.8, 137.4, 128.7, 125.2, 120.2, 116.7, 52.3, 36.1, 33.2, 29.9, 21.4; HRMS (ESI) Calcd for $C_{12}H_{16}NOS$ $[M + H]^+$ 222.0947, found 222.0952.

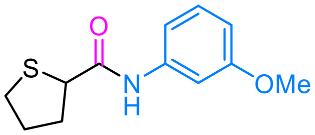

***N*-(3-Methoxyphenyl)tetrahydrothiophene-2-carboxamide (3l)** Prepared according to the general procedure from **1a** (1 mL, 0.2 M) and **2l** (0.20 mmol) purified by column chromatography on silica gel with petroleum ether/ethyl acetate (10:1) to provide the title compound **3l** as a colorless oil (33.7 mg, 71% yield); ^{1}H NMR (400 MHz, $CDCl_3$) δ 8.87 (s, 1H), 7.34 (t, *J* = 2.1 Hz, 1H), 7.22 (t, *J* = 8.1 Hz, 1H), 7.09-6.96 (m, 1H), 6.68 (dd, *J* = 8.0, 2.2 Hz, 1H), 4.05 (dd, *J* = 7.6, 4.4 Hz, 1H), 3.81 (s, 3H), 3.15-3.03 (m, 1H), 3.00-2.89 (m, 1H), 2.51-2.30 (m, 1H), 2.30-2.19 (m, 1H), 2.16-2.06 (m, 1H), 2.01-1.86 (m, 1H); ^{13}C NMR (101 MHz, $CDCl_3$) δ 170.2, 160.1, 138.7, 129.6, 111.7, 110.5, 105.1, 55.3, 52.4, 36.1, 33.3, 29.9; HRMS (ESI) Calcd for $C_{12}H_{16}NO_2S$ $[M + H]^+$ 238.0896, found 238.0899.

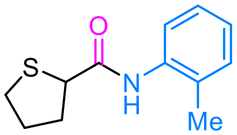

***N*-(*o*-Tolyl)tetrahydrothiophene-2-carboxamide (3m)** Prepared according to the general procedure from **1a** (1 mL, 0.2 M) and **2m** (0.20 mmol) purified by column chromatography on silica gel with petroleum ether/ethyl acetate (10:1) to provide the title compound **3m** as a white solid (36.3 mg, 82% yield); m.p. 103.4-105.8 °C; ^{1}H NMR (400 MHz, $CDCl_3$) δ 8.95 (s, 1H), 7.94 (d, *J* = 8.1 Hz, 1H), 7.20 (dd, *J* = 16.7, 8.0 Hz, 2H), 7.06 (t, *J* = 7.4 Hz, 1H), 4.10 (dd, *J* =

7.7, 4.1 Hz, 1H), 3.13-3.05 (m, 1H), 3.00-2.92 (m, 1H), 2.52-2.43 (m, 1H), 2.28 (s, 3H), 2.28-2.20 (m, 1H), 2.18-2.05 (m, 1H), 2.00-1.88 (m, 1H); ^{13}C NMR (101 MHz, $CDCl_3$) δ 169.9, 135.6, 130.4, 128.4, 126.8, 124.9, 121.7, 52.8, 36.4, 33.2, 29.8, 17.7; HRMS (ESI) Calcd for $C_{12}H_{16}NOS$ $[M + H]^+$ 222.0947, found 222.0947.

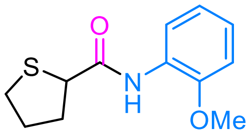

***N*-(2-Methoxyphenyl)tetrahydrothiophene-2-carboxamide (3n)** Prepared according to the general procedure from **1a** (1 mL, 0.2 M) and **2n** (0.20 mmol) purified by column chromatography on silica gel with petroleum ether/ethyl acetate (10:1) to provide the title compound **3n** as a light yellow oil (43.2 mg, 91% yield); ^{1}H NMR (400 MHz, $CDCl_3$) δ 9.34 (s, 1H), 8.36 (dd, J = 8.0, 1.7 Hz, 1H), 7.05 (td, J = 7.8, 1.7 Hz, 1H), 6.95 (td, J = 7.8, 1.5 Hz, 1H), 6.88 (dd, J = 8.1, 1.5 Hz, 1H), 4.06 (dd, J = 7.5, 4.4 Hz, 1H), 3.90 (s, 3H), 3.14-3.04 (m, 1H), 3.00-2.88 (m, 1H), 2.50-2.38 (m, 1H), 2.29-2.16 (m, 1H), 2.15-2.04 (m, 1H), 2.03-1.90 (m, 1H); ^{13}C NMR (101 MHz, $CDCl_3$) δ 170.1, 148.4, 127.3, 124.0, 120.9, 119.5, 110.0, 55.8, 52.6, 36.1, 33.2, 29.9; HRMS (ESI) Calcd for $C_{12}H_{16}NO_2S$ $[M + H]^+$ 238.0896, found 238.0896.

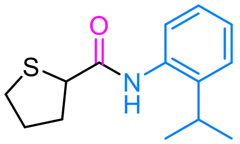

***N*-(2-Isopropylphenyl)tetrahydrothiophene-2-carboxamide (3o)** Prepared according to the general procedure from **1a** (1 mL, 0.2 M) and **2o** (0.20 mmol) purified by column chromatography on silica gel with petroleum ether/ethyl acetate (10:1) to provide the title compound **3o** as a white solid (39.8 mg, 80% yield); m.p. 87.9-90.2 °C; ^{1}H NMR (400 MHz, $CDCl_3$) δ 9.07 (s, 1H), 7.88 (dd, J = 7.9, 1.5 Hz, 1H), 7.28 (dd, J = 7.6, 1.8 Hz, 1H), 7.21 (td, J = 7.6, 1.8 Hz, 1H), 7.15 (td, J = 7.5, 1.5 Hz, 1H), 4.12 (dd, J = 7.6, 4.1 Hz, 1H), 3.12-2.93 (m, 3H), 2.53-2.44 (m, 1H), 2.32-2.21 (m, 1H), 2.19-2.09 (m, 1H), 1.99-1.88 (m, 1H); ^{13}C NMR (101 MHz, $CDCl_3$) δ 170.1, 139.1, 134.2, 126.4, 125.5, 125.4, 122.9, 52.8, 36.4, 33.1, 29.8, 28.1, 22.8, 22.7; HRMS (ESI) Calcd for $C_{14}H_{20}NOS$ $[M + H]^+$ 250.1260, found 250.1252.

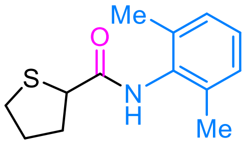

***N*-(2,6-Dimethylphenyl)tetrahydrothiophene-2-carboxamide (3p)** Prepared according to the general procedure from **1a** (1 mL, 0.2 M) and **2p** (0.20 mmol) purified by column chromatography on silica gel with petroleum ether/ethyl acetate (10:1) to provide the title compound **3p** as a white solid (39.8 mg, 85% yield); m.p. 137.2-138.8 °C; ^{1}H NMR (400 MHz, $CDCl_3$) δ 8.43 (s, 1H), 7.12-7.04 (m, 3H), 4.11 (dd, *J* = 7.6, 4.2 Hz, 1H), 3.13-3.06 (m, 1H), 3.01-2.93 (m, 1H), 2.51-2.43 (m, 1H), 2.29-2.23 (m, 1H), 2.21 (s, 6H), 2.18-2.13 (m, 1H), 2.08-1.96 (m, 1H); ^{13}C NMR (101 MHz, $CDCl_3$) δ 170.3, 135.0, 133.7, 128.2, 127.2, 51.9, 36.3, 33.2, 30.0, 18.4; HRMS (ESI) Calcd for $C_{13}H_{18}NOS$ $[M + H]^+$ 236.1104, found 236.1109.

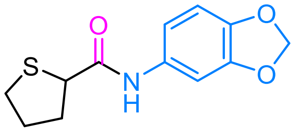

***N*-(Benzo[*d*][1,3]dioxol-5-yl)tetrahydrothiophene-2-carboxamide (3q)** Prepared according to the general procedure from **1a** (1 mL, 0.2 M) and **2q** (0.20 mmol) purified by column chromatography on silica gel with petroleum ether/ethyl acetate (10:1) to provide the title compound **3q** as a yellow solid (27.1 mg, 54% yield); m.p. 109.3-110.7 °C; ^{1}H NMR (400 MHz, $CDCl_3$) δ 8.80 (s, 1H), 7.27 (d, *J* = 2.3 Hz, 1H), 6.84 (dd, *J* = 8.3, 2.1 Hz, 1H), 6.74 (d, *J* = 8.3 Hz, 1H), 5.94 (s, 2H), 4.03 (dd, *J* = 7.5, 4.3 Hz, 1H), 3.12-3.01 (m, 1H), 2.99-2.88 (m, 1H), 2.49-2.36 (m, 1H), 2.29-2.17 (m, 1H), 2.16-2.04 (m, 1H), 1.99-1.86 (m, 1H); ^{13}C NMR (101 MHz, $CDCl_3$) δ 169.9, 147.8, 144.4, 131.8, 112.9, 108.0, 102.4, 101.2, 52.3, 36.2, 33.3, 29.9; HRMS (ESI) Calcd for $C_{12}H_{14}NO_3S$ $[M + H]^+$ 252.0689, found 252.0685.

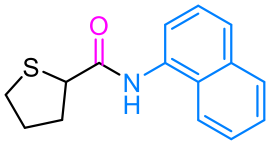

***N*-(Naphthalen-1-yl)tetrahydrothiophene-2-carboxamide (3r)** Prepared according to the general procedure from **1a** (1 mL, 0.2 M) and **2r** (0.20 mmol) purified by column chromatography on silica gel with petroleum ether/ethyl acetate (10:1) to provide the title compound **3r** as a white solid (36.0 mg, 70% yield); m.p. 123.7-125.5 °C; ^{1}H NMR (400 MHz, $CDCl_3$) δ 9.59 (s, 1H), 8.10 (d, *J* = 7.6 Hz, 1H), 7.96-7.81 (m, 2H), 7.68 (d, *J* = 8.2 Hz, 1H),

7.60-7.44 (m, 3H), 4.22 (dd, J = 7.8, 4.1 Hz, 1H), 3.24-3.13 (m, 1H), 3.10-2.94 (m, 1H), 2.64-2.47 (m, 1H), 2.38-2.25 (m, 1H), 2.24-2.12 (m, 1H), 2.07-1.95 (tt, J = 14.3, 8.6 Hz, 1H); ^{13}C NMR (101 MHz, $CDCl_3$) δ 170.3, 134.0, 132.1, 128.8, 126.6, 126.4, 126.0, 125.8, 125.4, 120.3, 119.2, 53.0, 36.5, 33.4, 29.9; HRMS (ESI) Calcd for $C_{15}H_{16}NOS$ $[M + H]^+$ 258.0947, found 258.0939.

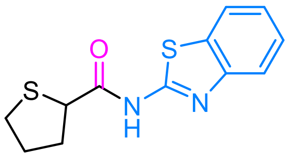

***N*-(Benzo[*d*]thiazol-2-yl)tetrahydrothiophene-2-carboxamide (3s)** Prepared according to the general procedure from **1a** (1 mL, 0.2 M) and **2s** (0.20 mmol) purified by column chromatography on silica gel with petroleum ether/ethyl acetate (10:1) to provide the title compound **3s** as a white solid (36.8 mg, 69% yield); m.p. 119.3-120.9 °C; ^1H NMR (400 MHz, $CDCl_3$) δ 10.57 (s, 1H), 7.82 (t, J = 7.5 Hz, 2H), 7.49-7.41 (m, 1H), 7.32 (td, J = 7.5, 1.2 Hz, 1H), 4.14 (dd, J = 7.6, 4.3 Hz, 1H), 3.12-3.03 (m, 1H), 3.01-2.91 (m, 1H), 2.52-2.42 (m, 1H), 2.31-2.19 (m, 1H), 2.18-2.07 (m, 1H), 2.06-1.93 (m, 1H); ^{13}C NMR (101 MHz, $CDCl_3$) δ 171.2, 157.7, 148.3, 132.1, 126.3, 124.1, 121.4, 121.0, 51.0, 35.8, 33.4, 30.1; HRMS (ESI) Calcd for $C_{12}H_{13}N_2OS_2$ $[M + H]^+$ 265.0464, found 265.0459.

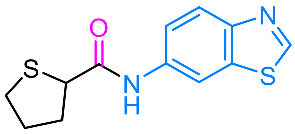

***N*-(Benzo[*d*]thiazol-6-yl)tetrahydrothiophene-2-carboxamide (3t)** Prepared according to the general procedure from **1a** (1 mL, 0.2 M) and **2t** (0.20 mmol) purified by column chromatography on silica gel with petroleum ether/ethyl acetate (10:1) to provide the title compound **3t** as a yellow solid (30.0 mg, 57% yield); m.p. 97.4-99.7 °C; ^1H NMR (400 MHz, $CDCl_3$) δ 9.13 (s, 1H), 8.92 (s, 1H), 8.59 (d, J = 2.1 Hz, 1H), 8.06 (d, J = 8.7 Hz, 1H), 7.41 (dd, J = 8.8, 2.2 Hz, 1H), 4.10 (dd, J = 7.6, 4.4 Hz, 1H), 3.16-3.07 (m, 1H), 3.01-2.94 (m, 1H), 2.52-2.43 (m, 1H), 2.32-2.23 (m, 1H), 2.18-2.10 (m, 1H), 2.01-1.90 (m, 1H); ^{13}C NMR (101 MHz, $CDCl_3$) δ 170.4, 153.5, 150.0, 135.3, 134.7, 123.5, 118.8, 112.2, 52.3, 36.1, 33.3, 30.0; HRMS (ESI) Calcd for $C_{12}H_{13}N_2OS_2$ $[M + H]^+$ 265.0464, found 265.0465.

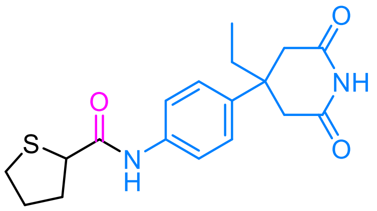

N-(4-(4-Ethyl-2,6-dioxopiperidin-4-yl)phenyl)tetrahydrothiophene-2-carboxamide (3u) Prepared according to the general procedure from **1a** (1 mL, 0.2 M) and **2u** (0.20 mmol) purified by column chromatography on silica gel with petroleum ether/ethyl acetate (10:1-5:1) to provide the title compound **3u** as a dark yellow oil (39.4 mg, 57% yield); ^{1}H NMR (400 MHz, $CDCl_3$) δ 8.92 (s, 1H), 7.99 (s, 1H), 7.58 (d, J = 8.8 Hz, 2H), 7.24 (d, J = 8.8 Hz, 2H), 4.06 (dd, J = 7.5, 4.4 Hz, 1H), 3.12-3.04 (m, 1H), 2.99-2.92 (m, 1H), 2.60 (dt, J = 15.4, 2.2 Hz, 1H), 2.47-2.39 (m, 2H), 2.38-2.33 (m, 1H), 2.29-2.20 (m, 2H), 2.14-2.08 (m, 1H), 2.05-2.00 (m, 1H), 1.96-1.86 (m, 2H), 0.86 (t, J = 7.4 Hz, 3H); ^{13}C NMR (101 MHz, $CDCl_3$) δ 175.0, 172.1, 170.3, 136.9, 134.6, 126.8, 120.1, 52.3, 50.7, 36.2, 33.3, 32.9, 29.9, 29.2, 27.0, 9.0; HRMS (ESI) Calcd for $C_{18}H_{23}N_2O_3S$ $[M + H]^+$ 347.1424, found 347.1419.

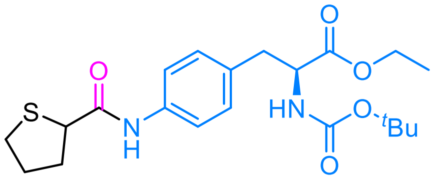

Ethyl (2S)-2-((*tert*-butoxycarbonyl)amino)-3-(4-(tetrahydrothiophene-2-carboxamido)phenyl)propanoate (3v) Prepared according to the general procedure from **1a** (1 mL, 0.2 M) and **2v** (0.20 mmol) purified by column chromatography on silica gel with petroleum ether/ethyl acetate (10:1-5:1) to provide the title compound **3v** as a white solid (43.2 mg, 51% yield); m.p. 128.4-130.1 °C; ^{1}H NMR (400 MHz, $CDCl_3$) δ 8.88 (s, 1H), 7.49 (d, J = 8.5 Hz, 2H), 7.10 (d, J = 8.5 Hz, 2H), 4.98 (d, J = 8.2 Hz, 1H), 4.52 (q, J = 6.5 Hz, 1H), 4.16 (q, J = 7.1 Hz, 2H), 4.04 (dd, J = 7.5, 4.4 Hz, 1H), 3.16-2.98 (m, 3H), 2.98-2.91 (m, 1H), 2.50-2.38 (m, 1H), 2.29-2.18 (m, 1H), 2.16-2.06 (m, 1H), 2.00-1.86 (m, 1H), 1.72 (s, 1H), 1.42 (s, 9H), 1.25 (t, J = 7.1 Hz, 3H); ^{13}C NMR (101 MHz, $CDCl_3$) δ 171.7, 170.1, 155.1, 136.4, 132.2, 129.9, 119.7, 79.8, 61.4, 54.4, 52.4, 37.6, 36.2, 33.3, 29.9, 28.3, 14.1; HRMS (ESI) Calcd for $C_{21}H_{31}N_2O_5S$ $[M + H]^+$ 423.1948, found 423.1948.

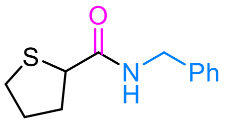

***N*-Benzyltetrahydrothiophene-2-carboxamide (3w)** Prepared according to the general procedure from **1a** (1 mL, 0.2 M) and **2w** (0.20 mmol) purified by column chromatography on silica gel with petroleum ether/ethyl acetate (10:1) to provide the title compound **3w** as a yellow solid (33.2 mg, 75% yield); m.p. 81.7-83.5 °C; ^{1}H NMR (400 MHz, $CDCl_3$) δ 7.36-7.31 (m, 2H), 7.27 (q, *J* = 7.6 Hz, 3H), 4.55-4.34 (m, 2H), 3.97 (dd, *J* = 7.6, 4.4 Hz, 1H), 3.03-2.83 (m, 2H), 2.42-2.31 (m, 1H), 2.24-2.12 (m, 1H), 2.10-2.00 (m, 1H), 1.96-1.82 (m, 1H); ^{13}C NMR (101 MHz, $CDCl_3$) δ 171.9, 138.1, 128.7, 127.6, 127.5, 51.6, 43.8, 36.0, 33.1, 29.9; HRMS (ESI) Calcd for $C_{12}H_{16}NOS$ $[M + H]^+$ 222.0947, found 222.0951.

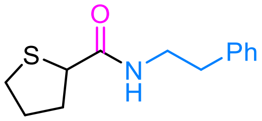

***N*-Phenethyltetrahydrothiophene-2-carboxamide (3x)** Prepared according to the general procedure from **1a** (1 mL, 0.2 M) and **2x** (0.20 mmol) purified by column chromatography on silica gel with petroleum ether/ethyl acetate (10:1) to provide the title compound **3x** as a dark red solid (33.9 mg, 72% yield); m.p. 89.7-91.9 °C; ^{1}H NMR (400 MHz, $CDCl_3$) δ 7.34-7.29 (m, 2H), 7.26-7.22 (m, 1H), 7.21-7.18 (m, 2H), 7.02 (s, 1H), 3.88 (dd, *J* = 7.5, 4.3 Hz, 1H), 3.58-3.45 (m, 2H), 2.89-2.79 (m, 4H), 2.32-2.24 (m, 1H), 2.17-2.07 (m, 1H), 2.05-1.96 (m, 1H), 1.80-1.69 (m, 1H); ^{13}C NMR (101 MHz, $CDCl_3$) δ 171.9, 138.7, 128.8, 128.6, 126.5, 51.7, 40.9, 36.0, 35.6, 32.9, 29.7; HRMS (ESI) Calcd for $C_{13}H_{18}NOS$ $[M + H]^+$ 236.1104, found 236.1109.

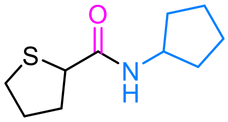

***N*-Cyclopentyltetrahydrothiophene-2-carboxamide (3y)** Prepared according to the general procedure from **1a** (1 mL, 0.2 M) and **2y** (0.20 mmol) purified by column chromatography on silica gel with petroleum ether/ethyl acetate (20:1) to provide the title compound **3ya** as a white solid (34.7 mg, 87% yield); m.p. 110.8-112.5 °C; ^{1}H NMR (400 MHz, $CDCl_3$) δ 6.94 (s, 1H), 4.23-4.09 (m, 1H), 3.89 (dd, *J* = 7.6, 4.5 Hz, 1H), 3.03-2.83 (m, 2H), 2.36-2.27 (m, 1H), 2.21-2.10 (m, 1H), 2.08-1.93 (m, 3H), 1.92-1.90 (m, 1H), 1.71-1.56 (m, 4H), 1.44-1.32 (m, 2H); ^{13}C NMR (101 MHz, $CDCl_3$) δ 171.4, 51.6, 51.3, 36.0, 33.1, 33.0, 32.9, 29.8, 23.69, 23.68; HRMS

(ESI) Calcd for $C_{10}H_{18}NOS$ $[M + H]^+$ 200.1104, found 200.1110.

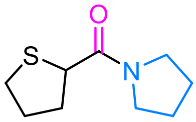

Pyrrolidin-1-yl(tetrahydrothiophen-2-yl)methanone (3z) Prepared according to the general procedure from **1a** (1 mL, 0.2 M) and **2z** (0.20 mmol) purified by column chromatography on silica gel with petroleum ether/ethyl acetate (20:1) to provide the title compound **3z** as a yellow oil (24.8 mg, 67% yield); ^{1}H NMR (400 MHz, $CDCl_3$) δ 3.96 (t, J = 5.8 Hz, 1H), 3.56-3.37 (m, 4H), 3.05-2.84 (m, 2H), 2.52-2.42 (m, 1H), 2.38-2.26 (m, 1H), 2.09-2.00 (m, 1H), 2.00-1.90 (m, 3H), 1.90-1.81 (m, 2H); ^{13}C NMR (101 MHz, $CDCl_3$) δ 171.0, 46.8, 46.7, 46.0, 33.2, 33.0, 31.3, 26.2, 24.2; HRMS (ESI) Calcd for $C_9H_{16}NOS$ $[M + H]^+$ 186.0947, found 186.0956.

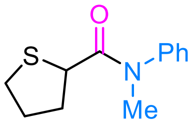

***N*-Methyl-*N*-phenyltetrahydrothiophene-2-carboxamide (3aa)** Prepared according to the general procedure from **1a** (1 mL, 0.2 M) and **2aa** (0.20 mmol) purified by column chromatography on silica gel with petroleum ether/ethyl acetate (20:1) to provide the title compound **3aa** as a white solid (33.2 mg, 75% yield); m.p. 88.5-90.4 °C; ^{1}H NMR (400 MHz, $CDCl_3$) δ 7.47-7.40 (m, 2H), 7.39-7.33 (m, 1H), 7.26-7.22 (m, 2H), 3.78 (t, J = 5.9 Hz, 1H), 3.28 (s, 3H), 3.03-2.91 (m, 1H), 2.83-2.71 (m, 1H), 2.40-2.27 (m, 2H), 1.94-1.79 (m, 2H); ^{13}C NMR (101 MHz, $CDCl_3$) δ 173.2, 143.4, 129.8, 128.0, 127.8, 45.6, 37.8, 33.7, 33.5, 31.6; HRMS (ESI) Calcd for $C_{12}H_{16}NOS$ $[M + H]^+$ 222.0947, found 222.0953.

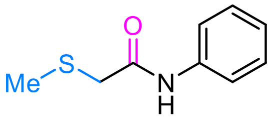

2-(Methylthio)-*N*-phenylacetamide (5a) Prepared according to the general procedure from **4a** (1 mL, 0.2 M) and **2a** (0.20 mmol) purified by column chromatography on silica gel with petroleum ether/ethyl acetate (10:1) to provide the title compound **5a** as a yellow oil (30.5 mg, 84% yield); ^{1}H NMR (400 MHz, $CDCl_3$) δ 8.71 (s, 1H), 7.57 (d, J = 7.4 Hz, 2H), 7.42-7.29 (m, 2H), 7.14 (t, J = 7.4 Hz, 1H), 3.34 (s, 2H), 2.19 (s, 3H); ^{13}C NMR (101 MHz, $CDCl_3$) δ 166.6, 137.4, 129.0, 124.6, 119.7, 39.0, 16.3; HRMS (ESI) Calcd for $C_9H_{12}NOS$ $[M + H]^+$ 182.0634, found 182.0640.

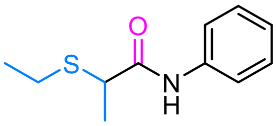

2-(Ethylthio)-*N*-phenylpropanamide (5b) Prepared according to the general procedure from **4b** (1 mL, 0.2 M) and **2a** (0.20 mmol) purified by column chromatography on silica gel with petroleum ether/ethyl acetate (10:1) to provide the title compound **5b** as a white solid (30.1 mg, 72% yield); m.p. 78.9-80.4 °C; ^{1}H NMR (400 MHz, $CDCl_3$) δ 8.66 (s, 1H), 7.56 (d, *J* = 7.1 Hz, 2H), 7.34 (t, *J* = 7.9 Hz, 2H), 7.12 (t, *J* = 7.5 Hz, 1H), 3.54 (q, *J* = 7.3 Hz, 1H), 2.62 (q, *J* = 7.5 Hz, 2H), 1.55 (d, *J* = 7.3 Hz, 3H), 1.28 (t, *J* = 7.5 Hz, 3H); ^{13}C NMR (101 MHz, $CDCl_3$) δ 170.8, 137.6, 129.0, 124.4, 119.6, 45.3, 25.7, 18.5, 14.5; HRMS (ESI) Calcd for $C_{11}H_{16}NOS$ $[M + H]^+$ 210.0947, found 210.0957.

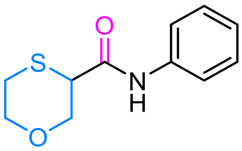

***N*-Phenyl-1,4-oxathiane-3-carboxamide (5c)** Prepared according to the general procedure from **4c** (1 mL, 0.2 M) and **2a** (0.20 mmol) purified by column chromatography on silica gel with petroleum ether/ethyl acetate (10:1) to provide the title compound **5c** as a light yellow oil (18.3 mg, 41% yield); ^{1}H NMR (400 MHz, $CDCl_3$) δ 8.99 (s, 1H), 7.60 (d, *J* = 8.4 Hz, 2H), 7.35 (t, *J* = 7.7 Hz, 2H), 7.19-7.10 (m, 1H), 4.68 (dd, *J* = 12.0, 3.1 Hz, 1H), 4.21-4.10 (m, 1H), 3.99 (dd, *J* = 12.0, 2.8 Hz, 1H), 3.91-3.79 (m, 1H), 3.27 (d, *J* = 3.2 Hz, 1H), 3.11-3.01 (m, 1H), 2.49-2.39 (m, 1H); ^{13}C NMR (101 MHz, $CDCl_3$) δ 167.3, 137.5, 129.0, 124.6, 119.8, 69.0, 68.2, 41.6, 25.1; HRMS (ESI) Calcd for $C_{11}H_{14}NO_2S$ $[M + H]^+$ 224.0740, found 224.0745.

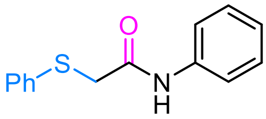

***N*-Phenyl-2-(phenylthio)acetamide (5d)** Prepared according to the general procedure from **4d** (1 mL, 0.2 M) and **2a** (0.20 mmol) purified by column chromatography on silica gel with petroleum ether/ethyl acetate (10:1) to provide the title compound **5d** as a yellow solid (26.8 mg, 55% yield); m.p. 71.3-73.5 °C; ^{1}H NMR (400 MHz, $CDCl_3$) δ 8.59 (s, 1H), 7.47 (d, *J* = 7.8 Hz, 2H), 7.37-7.28 (m, 6H), 7.22 (t, *J* = 7.1 Hz, 1H), 7.11 (t, *J* = 7.4 Hz, 1H), 3.75 (s, 2H); ^{13}C NMR (101 MHz, $CDCl_3$) δ 166.0, 137.2, 134.1, 129.5, 129.0, 128.4, 127.0, 124.7, 119.9, 38.4; HRMS (ESI) Calcd for $C_{14}H_{14}NOS$ $[M + H]^+$ 244.0791, found 244.8000.

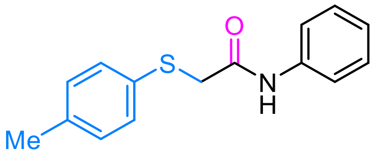

***N*-Phenyl-2-(*p*-tolylthio)acetamide (5e)** Prepared according to the general procedure from **4e** (1 mL, 0.2 M) and **2a** (0.20 mmol) purified by column chromatography on silica gel with petroleum ether/ethyl acetate (10:1) to provide the title compound **5e** as a yellow oil (29.3 mg, 57% yield); ^{1}H NMR (400 MHz, $CDCl_3$) δ 8.59 (s, 1H), 7.48 (dd, J = 8.6, 1.2 Hz, 2H), 7.34-7.29 (m, 2H), 7.28-7.24 (m, 2H), 7.15-7.08 (m, 3H), 3.71 (s, 2H), 2.30 (s, 3H); ^{13}C NMR (101 MHz, $CDCl_3$) δ 166.1, 137.4, 137.3, 130.4, 130.2, 129.2, 129.0, 124.7, 119.8, 39.1, 21.0; HRMS (ESI) Calcd for $C_{15}H_{16}NOS$ $[M + H]^+$ 258.0947, found 258.0956.

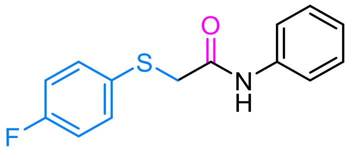

2-((4-Fluorophenyl)thio)-*N*-phenylacetamide (5f) Prepared according to the general procedure from **4f** (1 mL, 0.2 M) and **2a** (0.20 mmol) purified by column chromatography on silica gel with petroleum ether/ethyl acetate (10:1) to provide the title compound **5f** as a white solid (36.6 mg, 70% yield); m.p. 74.9-76.5 °C; ^{1}H NMR (400 MHz, $CDCl_3$) δ 8.49 (s, 1H), 7.48 (dd, J = 8.6, 1.2 Hz, 2H), 7.40-7.30 (m, 4H), 7.16-7.10 (m, 1H), 7.06-6.98 (m, 2H), 3.71 (s, 2H); ^{13}C NMR (101 MHz, $CDCl_3$) δ 165.9, 162.3 (d, $J_{C\text{-}F}$ = 246 Hz), 137.2, 131.5 (d, $J_{C\text{-}F}$ = 8 Hz), 129.1, 124.9, 119.9, 116.7 (d, $J_{C\text{-}F}$ = 22 Hz), 39.6; ^{19}F NMR (376 MHz, $CDCl_3$) δ -113.72; HRMS (ESI) Calcd for $C_{14}H_{13}FNOS$ $[M + H]^+$ 262.0696, found 262.0697.

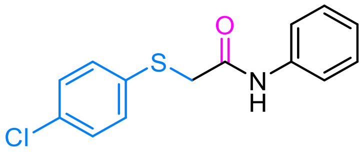

2-((4-Chlorophenyl)thio)-*N*-phenylacetamide (5g) Prepared according to the general procedure from **4g** (1 mL, 0.2 M) and **2a** (0.20 mmol) purified by column chromatography on silica gel with petroleum ether/ethyl acetate (10:1) to provide the title compound **5g** as a white solid (33.0 mg, 59% yield); m.p. 93.7-95.5 °C; ^{1}H NMR (400 MHz, $CDCl_3$) δ 8.47 (s, 1H), 7.47 (d, J = 7.8 Hz, 2H), 7.32 (t, J = 7.9 Hz, 2H), 7.28 (s, 4H), 7.13 (t, J = 7.4 Hz, 1H), 3.74 (s, 2H); ^{13}C NMR (101 MHz, $CDCl_3$) δ 165.6, 137.1, 133.2, 132.6, 129.8, 129.6, 129.1, 124.9, 119.9, 38.5; HRMS (ESI) Calcd for $C_{14}H_{13}ClNOS$ $[M + H]^+$ 278.0401, found 278.0409.

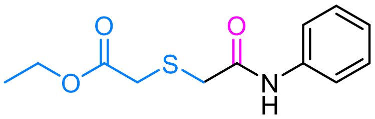

Ethyl 2-((2-oxo-2-(phenylamino)ethyl)thio)acetate (5h) Prepared according to the general procedure from **4h** (1 mL, 0.2 M) and **2a** (0.20 mmol) purified by column chromatography on silica gel with petroleum ether/ethyl acetate (10:1) to provide the title compound **5h** as a yellow solid (23.8 mg, 47% yield); m.p. 71.3-73.3 °C; ^{1}H NMR (400 MHz, $CDCl_3$) δ 8.88 (s, 1H), 7.59 (d, J = 7.8 Hz, 2H), 7.33 (t, J = 7.9 Hz, 2H), 7.12 (t, J = 7.4 Hz, 1H), 4.16 (q, J = 7.1 Hz, 2H), 3.47 (s, 2H), 3.37 (s, 2H), 1.25 (t, J = 7.1 Hz, 3H); ^{13}C NMR (101 MHz, $CDCl_3$) δ 170.2, 166.2, 137.6, 129.0, 124.5, 119.7, 62.0, 37.6, 34.8, 14.0; HRMS (ESI) Calcd for $C_{12}H_{16}NO_3S$ $[M + H]^+$ 254.0845, found 254.0850.

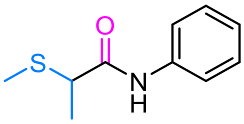

5i

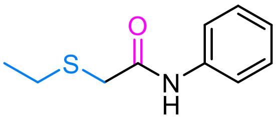

5i'

2-(Methylthio)-*N*-phenylpropanamide (5i) and 2-(Ethylthio)-*N*-phenylacetamide (5i') Prepared according to the general procedure from **4i** (1 mL, 0.2 M) and **2a** (0.20 mmol) purified by column chromatography on silica gel with petroleum ether/ethyl acetate (10:1) to provide the title compound **5i** as a dark yellow oil (26.2 mg, 67% yield, rr = 2:3); m.p. 91.4-93.1 °C; ^{1}H NMR (400 MHz, $CDCl_3$) δ 8.58 (s, 1H), 7.62-7.51 (m, 2H), 7.38-7.31 (m, 2H), 7.13 (t, J = 7.4 Hz, 1H), 3.47 (q, J = 7.3 Hz, 1H), 2.15 (s, 3H), 1.55 (d, J = 7.3 Hz, 3H); ^{13}C NMR (101 MHz, $CDCl_3$) δ 170.3, 137.7, 129.0, 124.4, 119.6, 46.4, 17.9, 14.1; HRMS (ESI) Calcd for $C_{10}H_{14}NOS$ $[M + H]^+$ 196.0791, found 196.0796.

(5i'): dark yellow oil; ^{1}H NMR (400 MHz, $CDCl_3$) δ 8.74 (s, 1H), 7.56 (d, J = 7.7 Hz, 2H), 7.35 (t, J = 7.9 Hz, 2H), 7.14 (t, J = 7.4 Hz, 1H), 3.38 (s, 2H), 2.63 (q, J = 7.4 Hz, 2H), 1.30 (t, J = 7.4 Hz, 3H); ^{13}C NMR (101 MHz, $CDCl_3$) δ 166.9, 137.4, 129.0, 124.6, 119.6, 36.5, 27.2, 14.4; HRMS (ESI) Calcd for $C_{10}H_{14}NOS$ $[M + H]^+$ 196.0791, found 196.0785.

Procedure for 2 mmol-scale reaction: Under an argon atmosphere, a 25 mL flask was charged with equipped with a magnetic stir bar was charged with $PdCl_2$ (35.4 mg, 0.2 mmol, 10 mol%), Xantphos (115.7 mg, 0.2 mmol, 10 mol%), **1a** sulfides (10 mL, 0.2 M), **2a** amines (1 equiv., 2 mmol) and DTBP (4.0 equiv.) were added nitrogen atmosphere, the vial was moved to an alloy plate and put into a Parr 4560 series autoclave (300 mL) under an argon atmosphere. At room temperature, the autoclave was flushed with CO three times and charged with 60 bar of CO. The autoclave was placed on a heating plate equipped with a magnetic stirrer and an aluminum block. The reaction mixture was heated to 120 ºC for 22 h. After the reaction was complete, the autoclave was cooled down with ice water to room temperature and the pressure was released carefully. After cooling to room temperature, the reaction mixture was directly purified by column chromatography on silica gel using petroleum ether and ethyl acetate to afford the corresponding product.

6. Reference

[1] J. T. Wróbel, E. Hejchman, *Synthesis* **1987**, *5*, 452-455.

7. NMR Spectra

^{1}H NMR (400 MHz, CDCl3) and ^{13}C NMR (101 MHz, CDCl3) spectrum of 3a

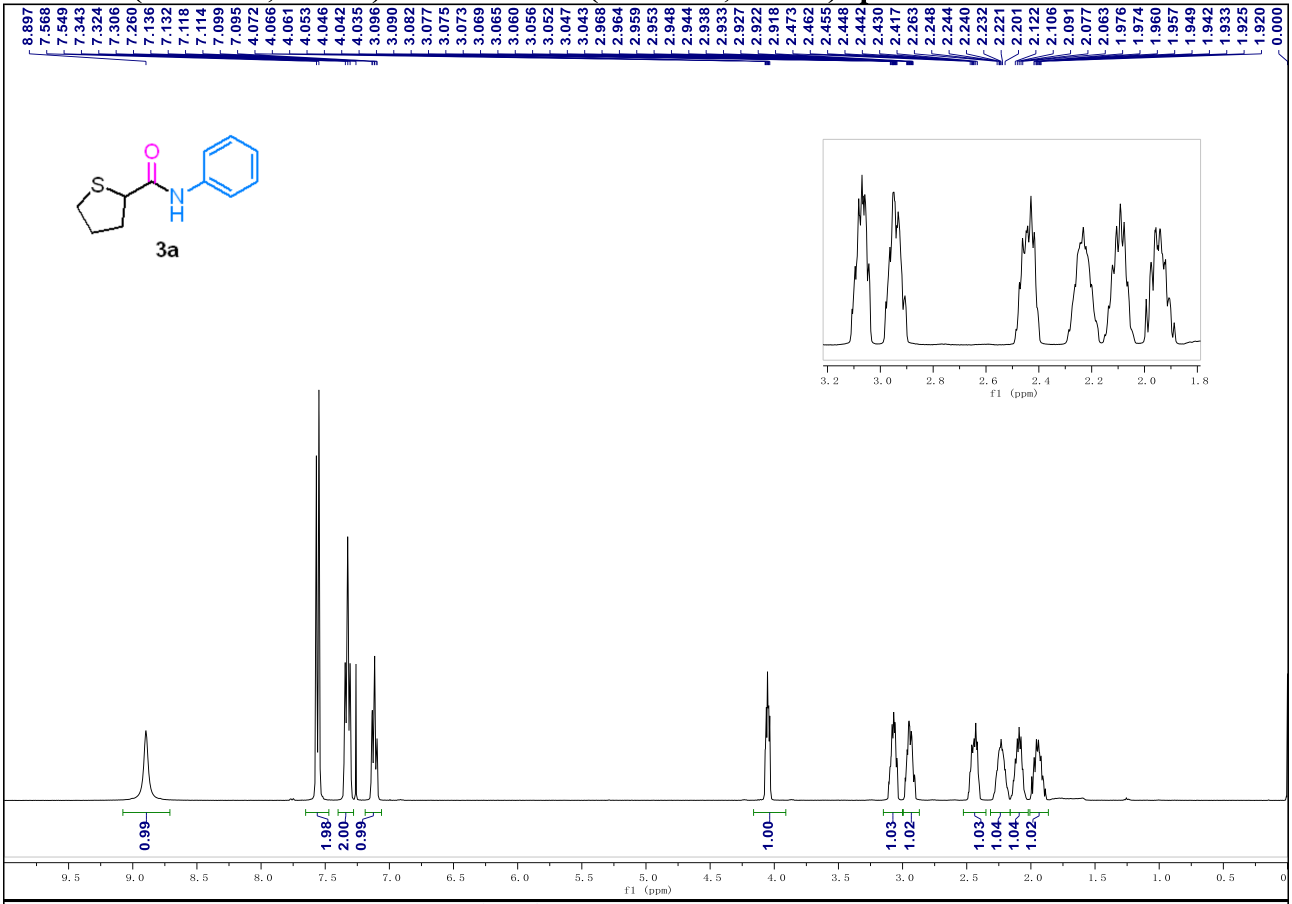

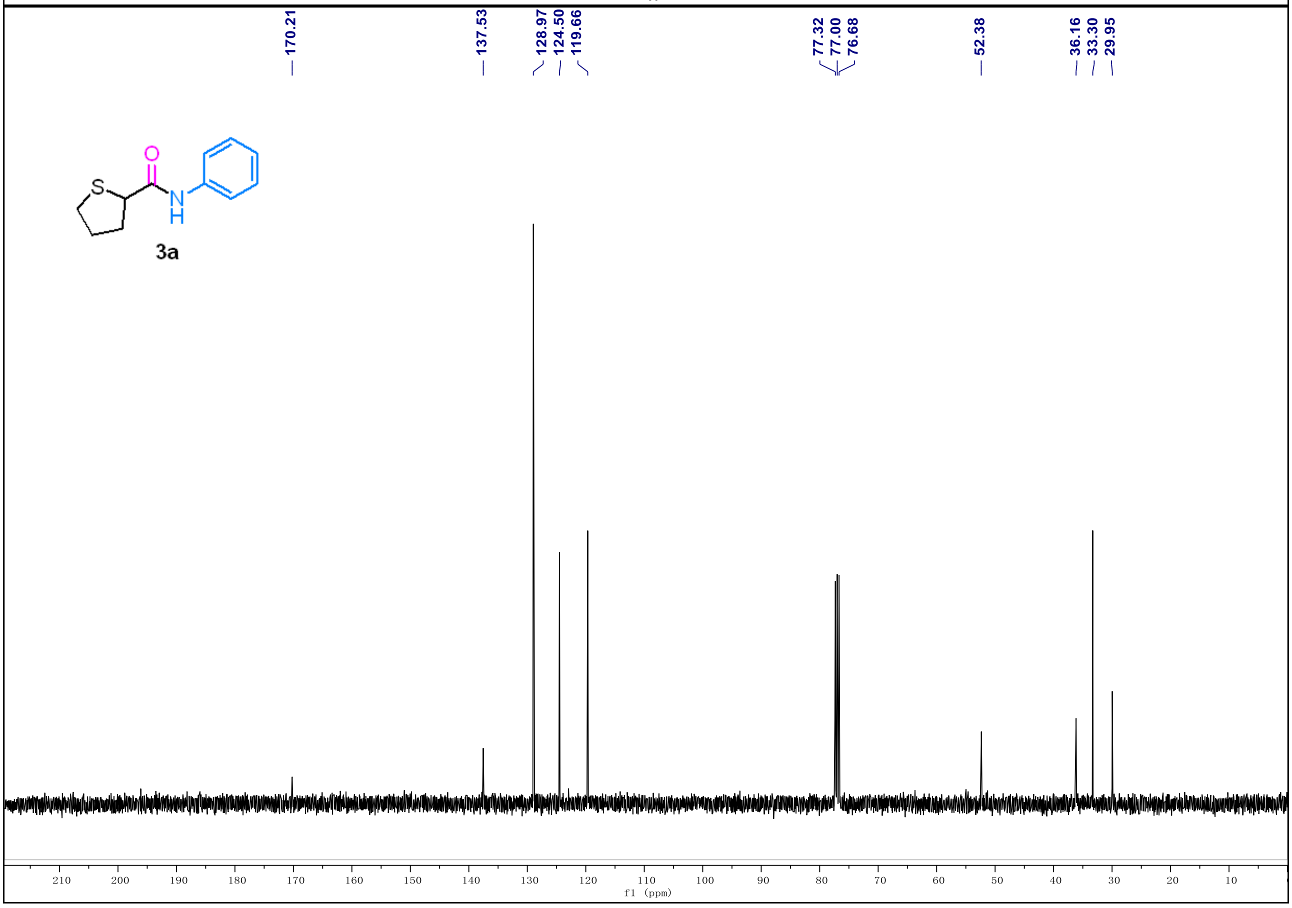

^{1}H NMR (400 MHz, $CDCl_3$) and ^{13}C NMR (101 MHz, $CDCl_3$) spectrum of 3b

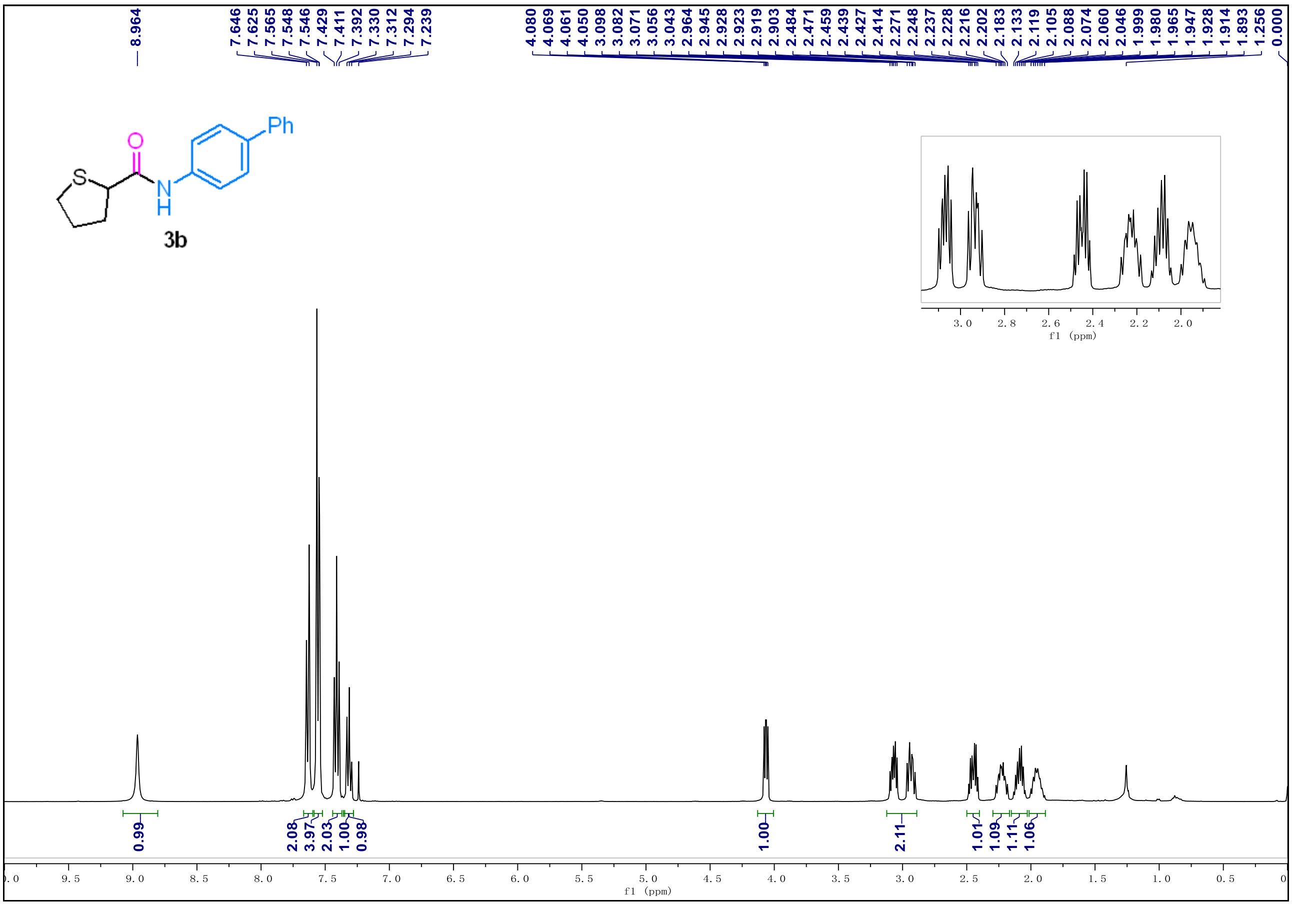

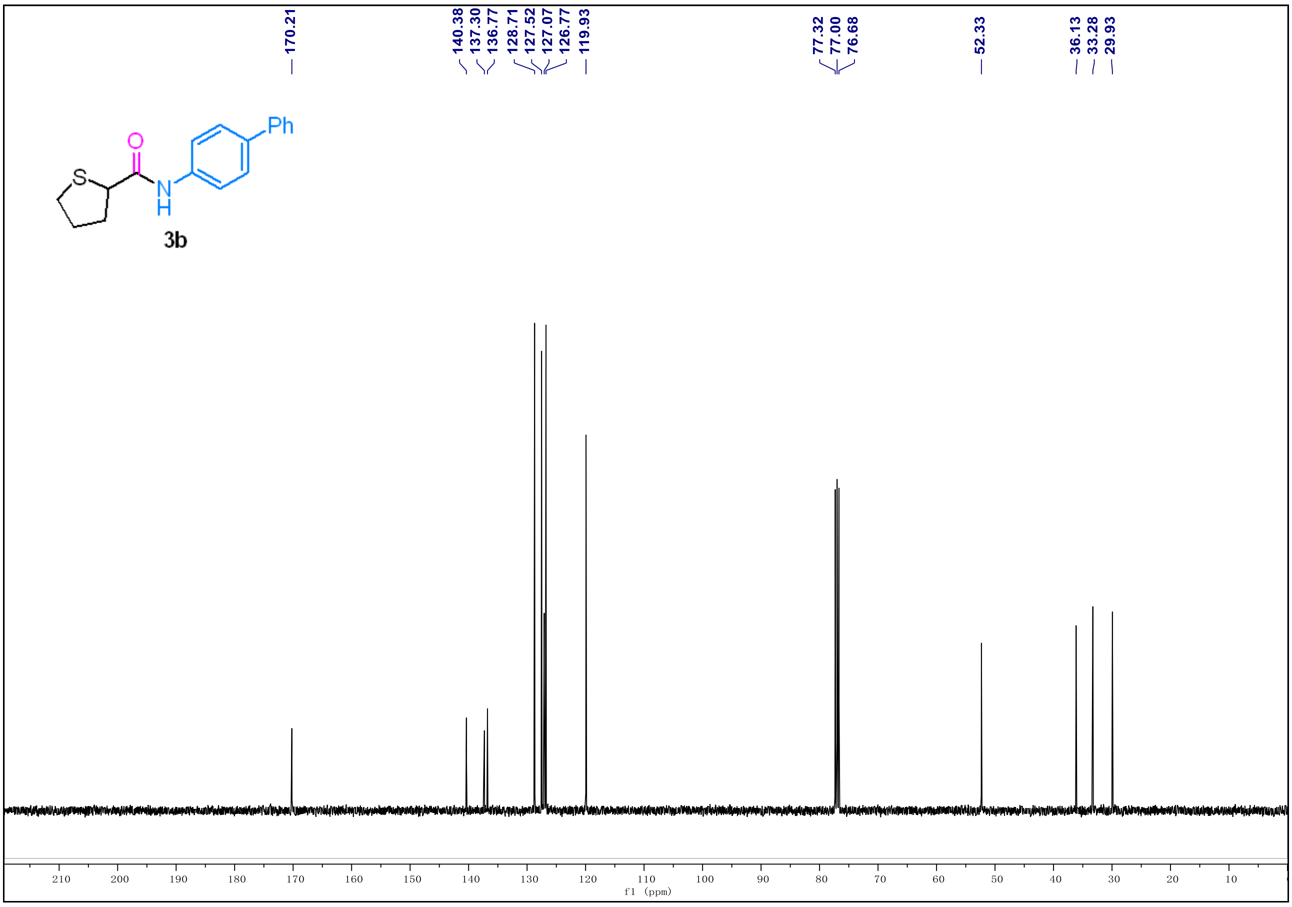

^{1}H NMR (400 MHz, CDCl$_3$) and ^{13}C NMR (101 MHz, CDCl$_3$) spectrum of 3c

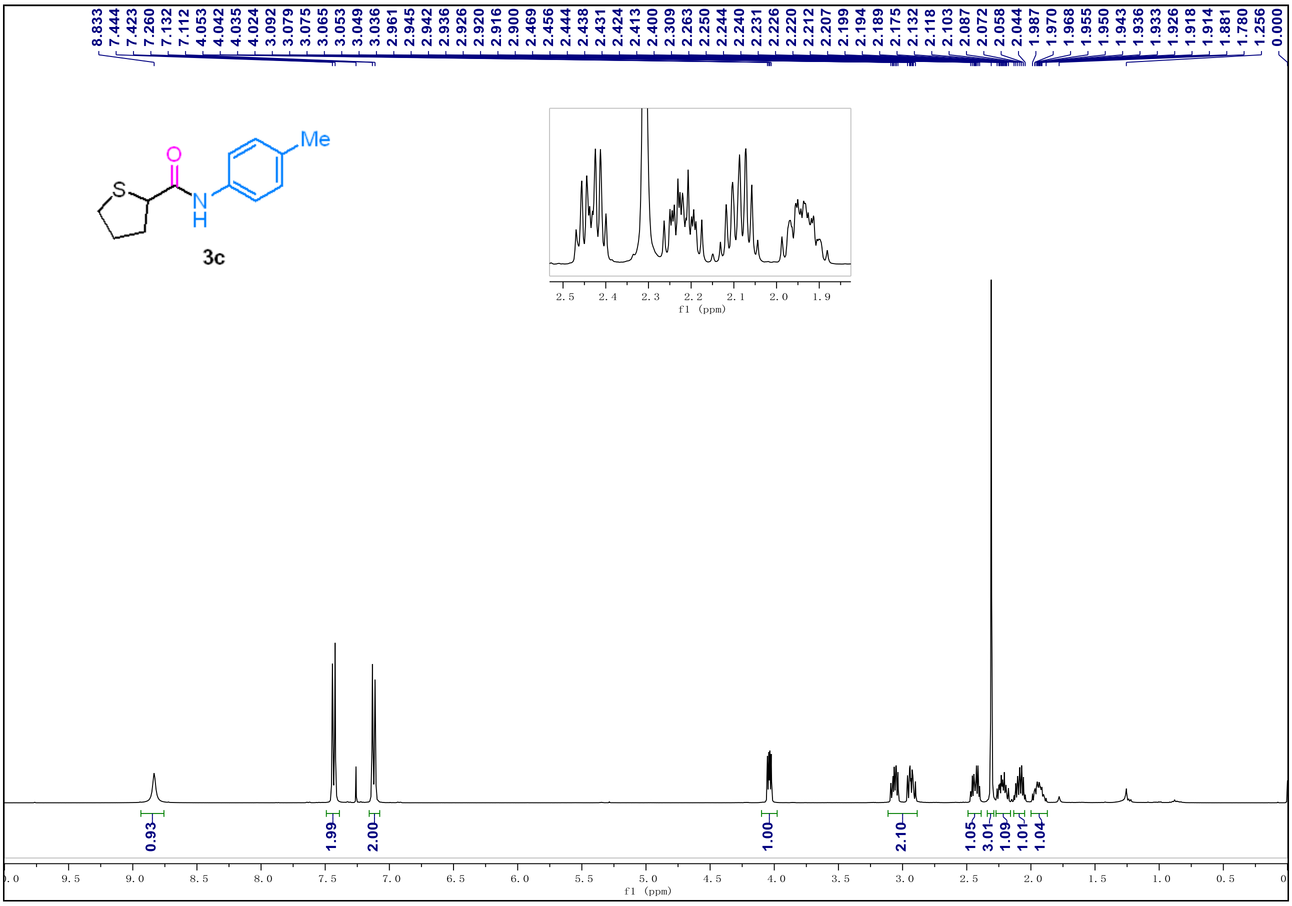

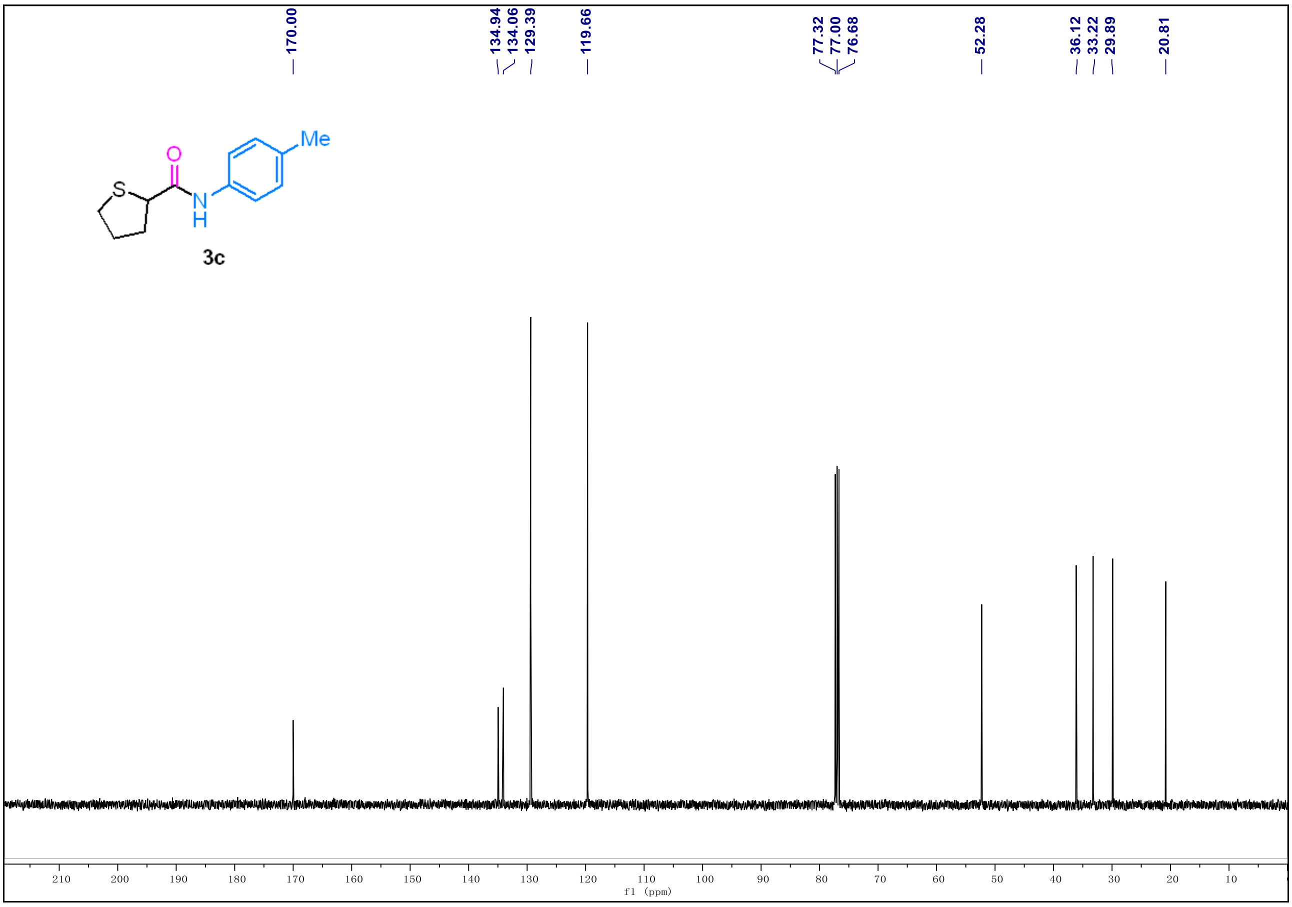

^{1}H NMR (400 MHz, $CDCl_3$) and ^{13}C NMR (101 MHz, $CDCl_3$) spectrum of 3d

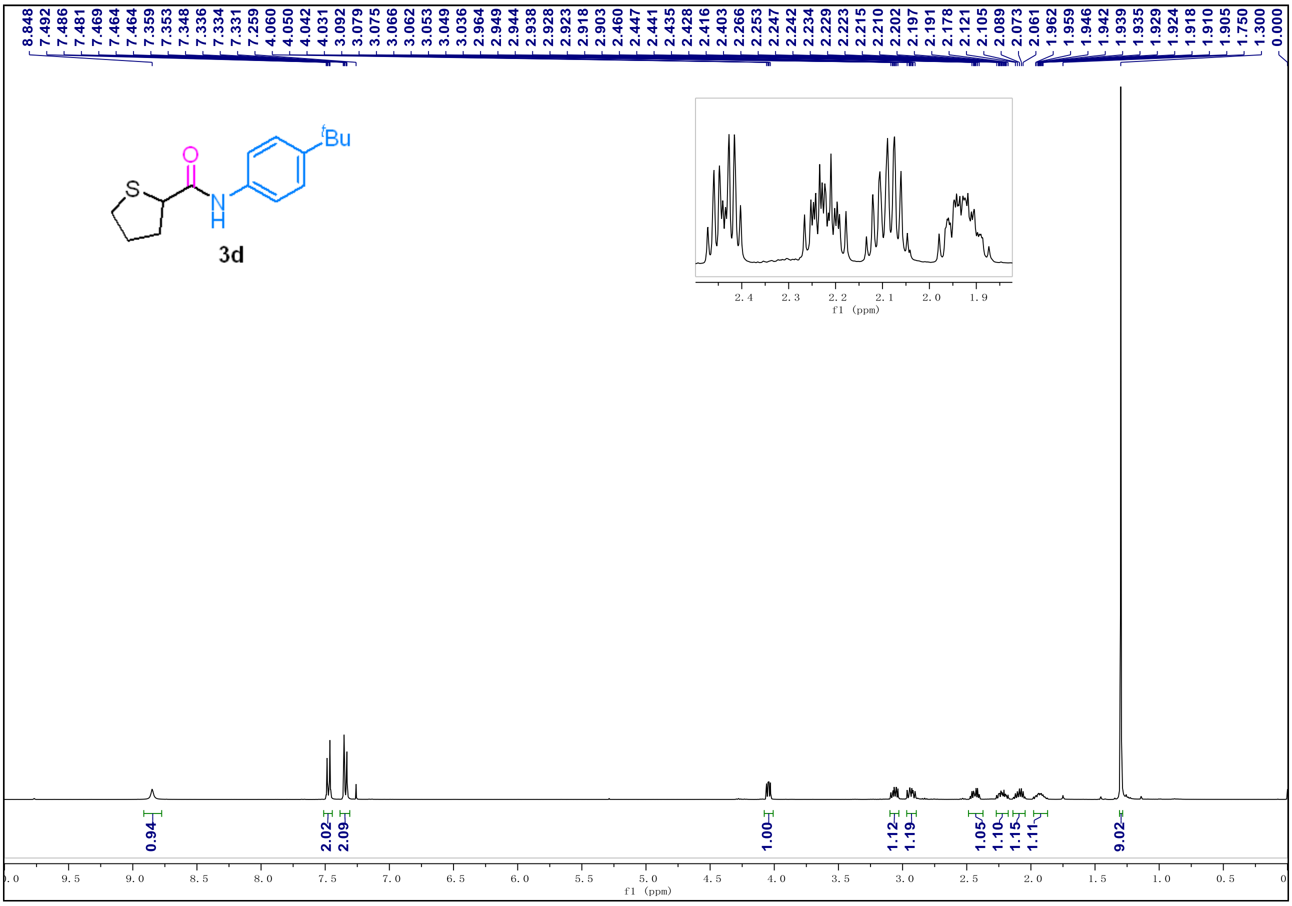

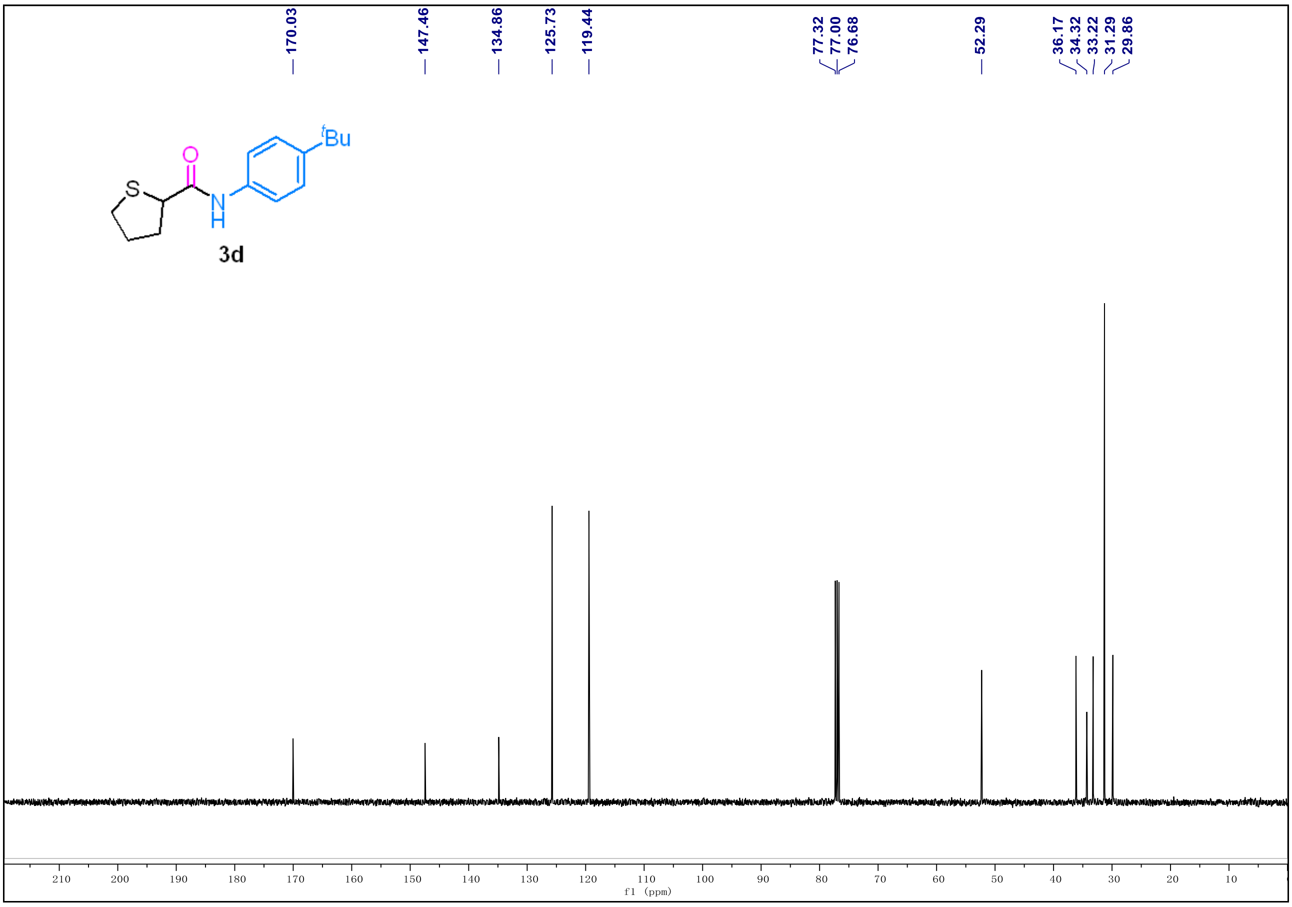

^{1}H NMR (400 MHz, CDCl3) and ^{13}C NMR (101 MHz, CDCl3) spectrum of 3e

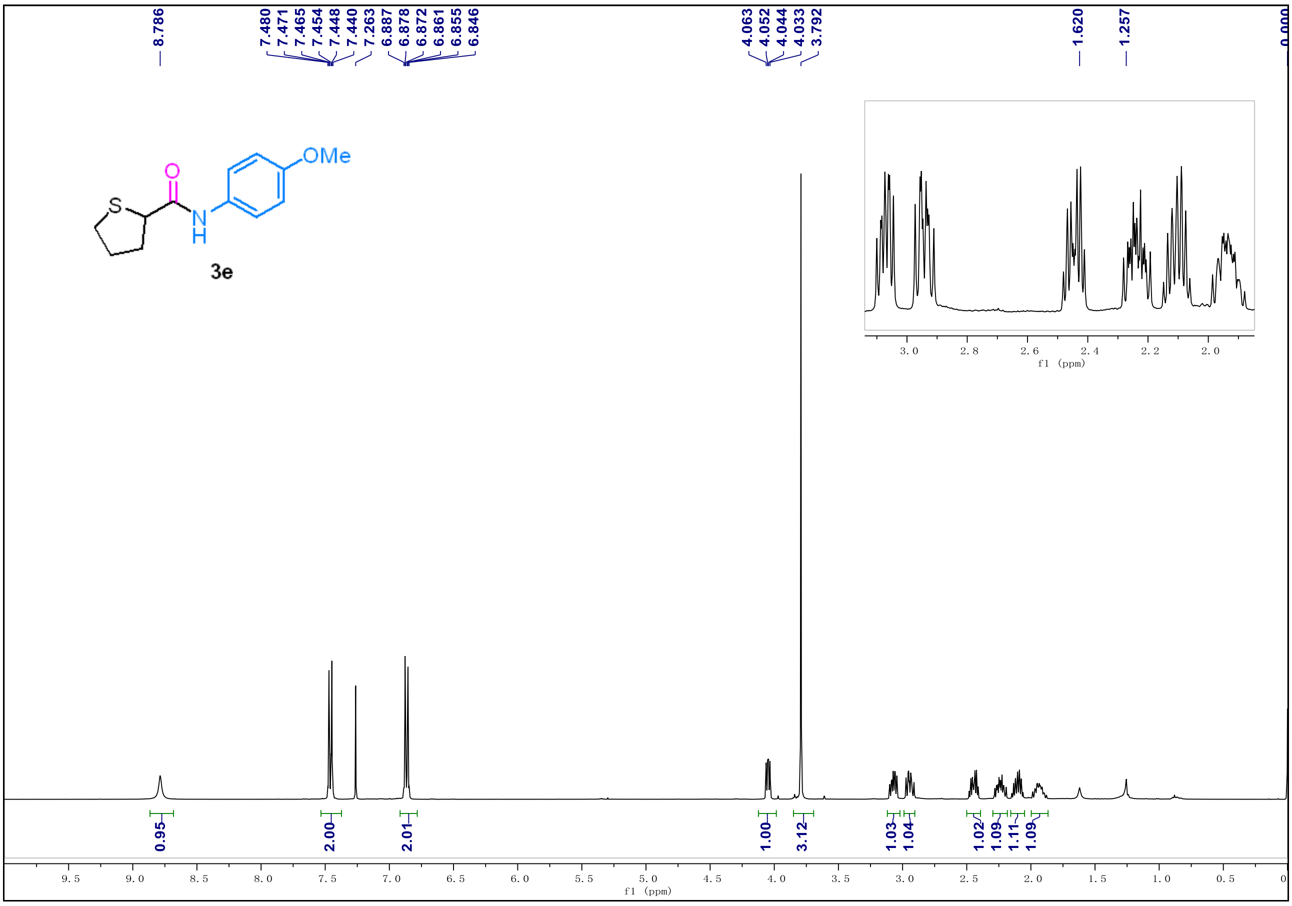

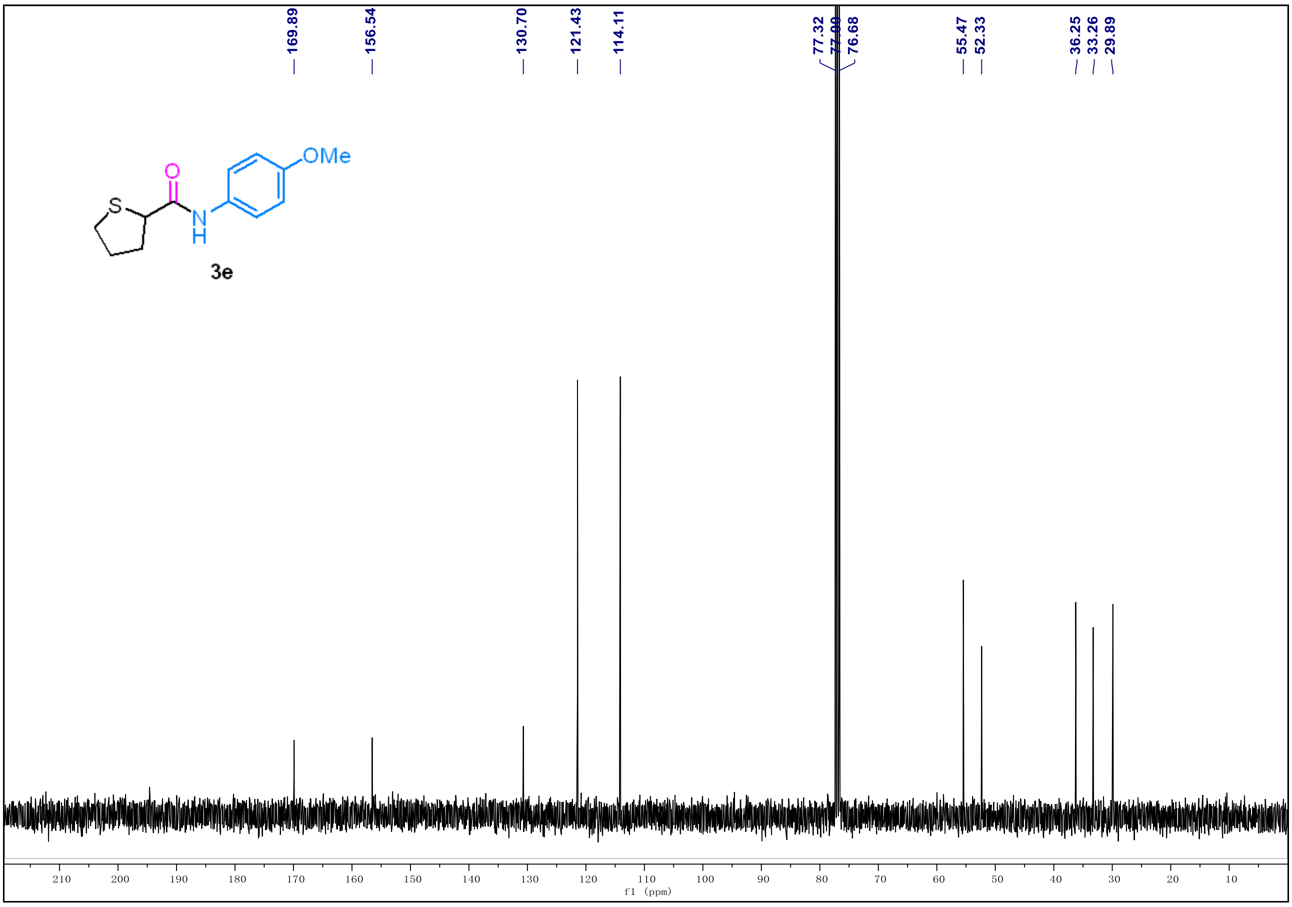

^{1}H NMR (400 MHz, CDCl3) and ^{13}C NMR (101 MHz, CDCl3) spectrum of 3f

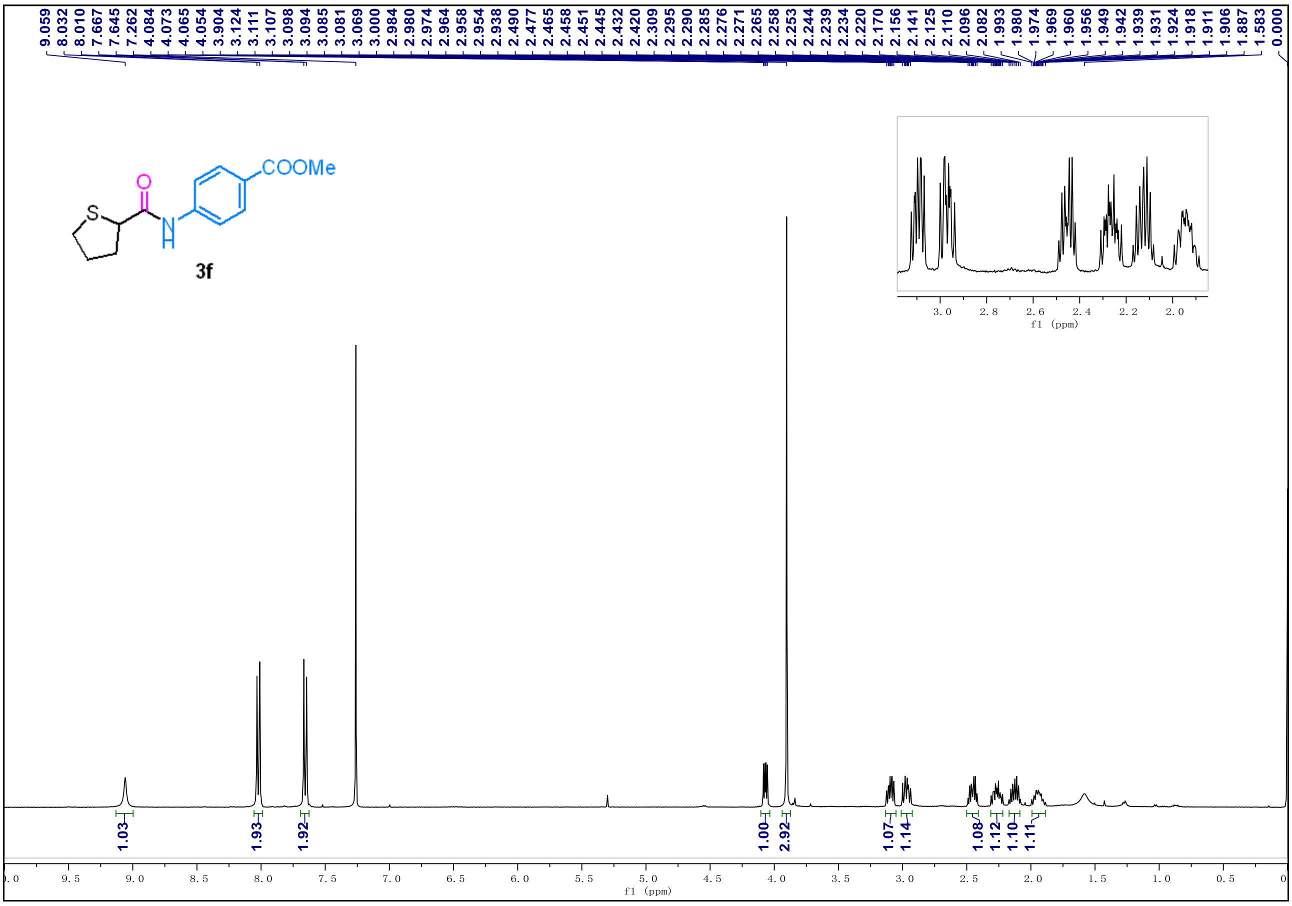

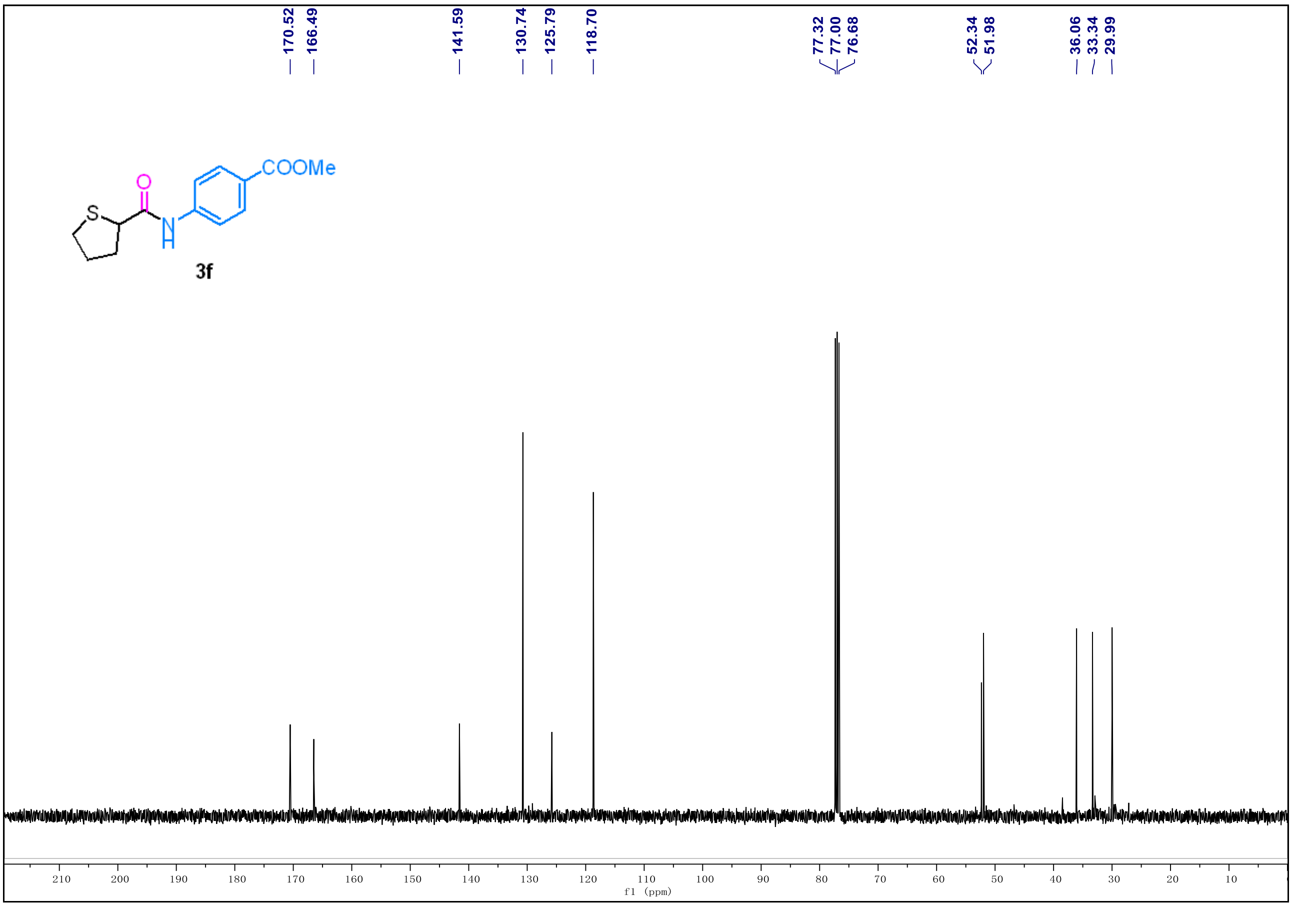

^{1}H NMR (400 MHz, $CDCl_3$) and ^{13}C NMR (101 MHz, $CDCl_3$) spectrum of 3g

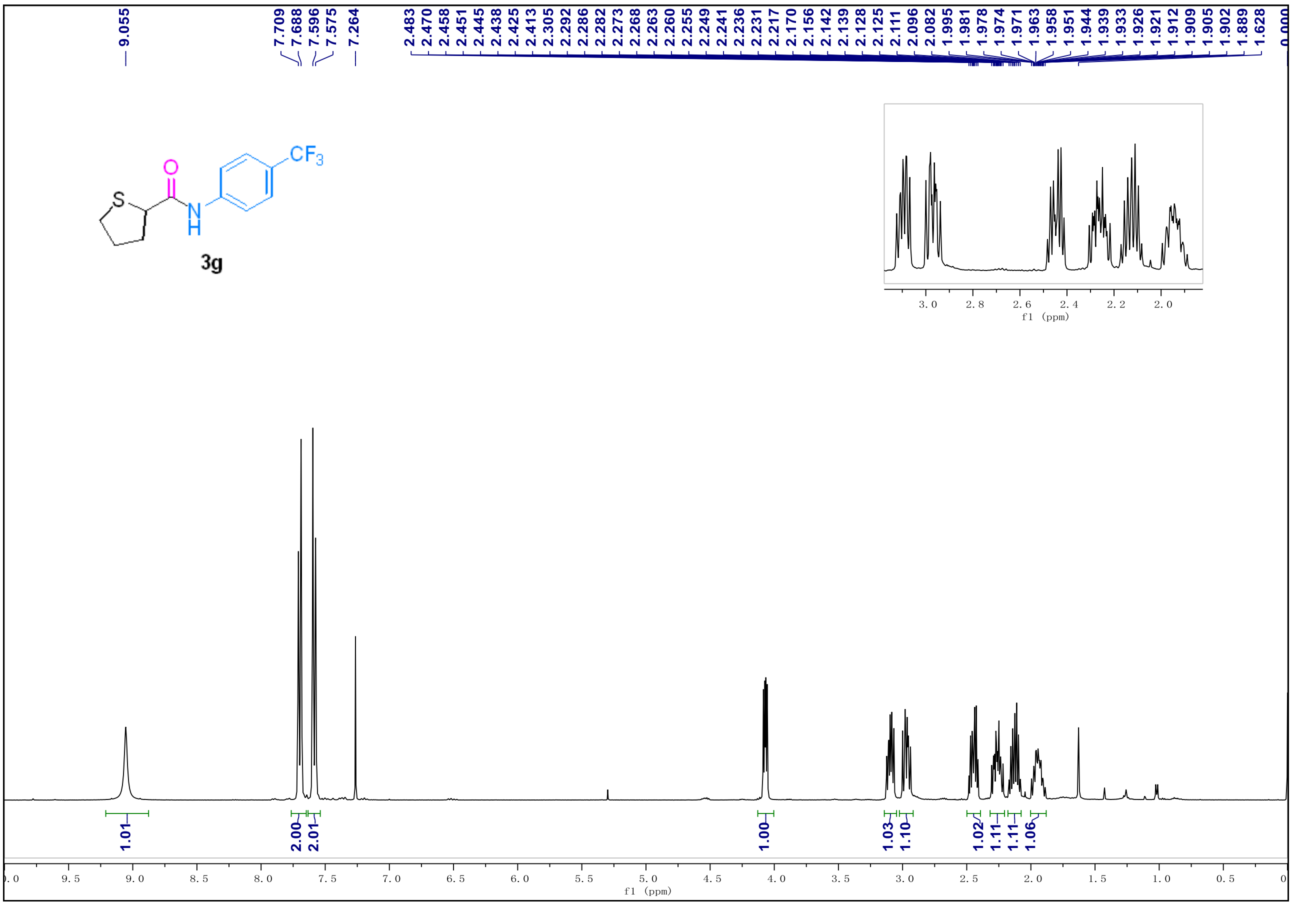

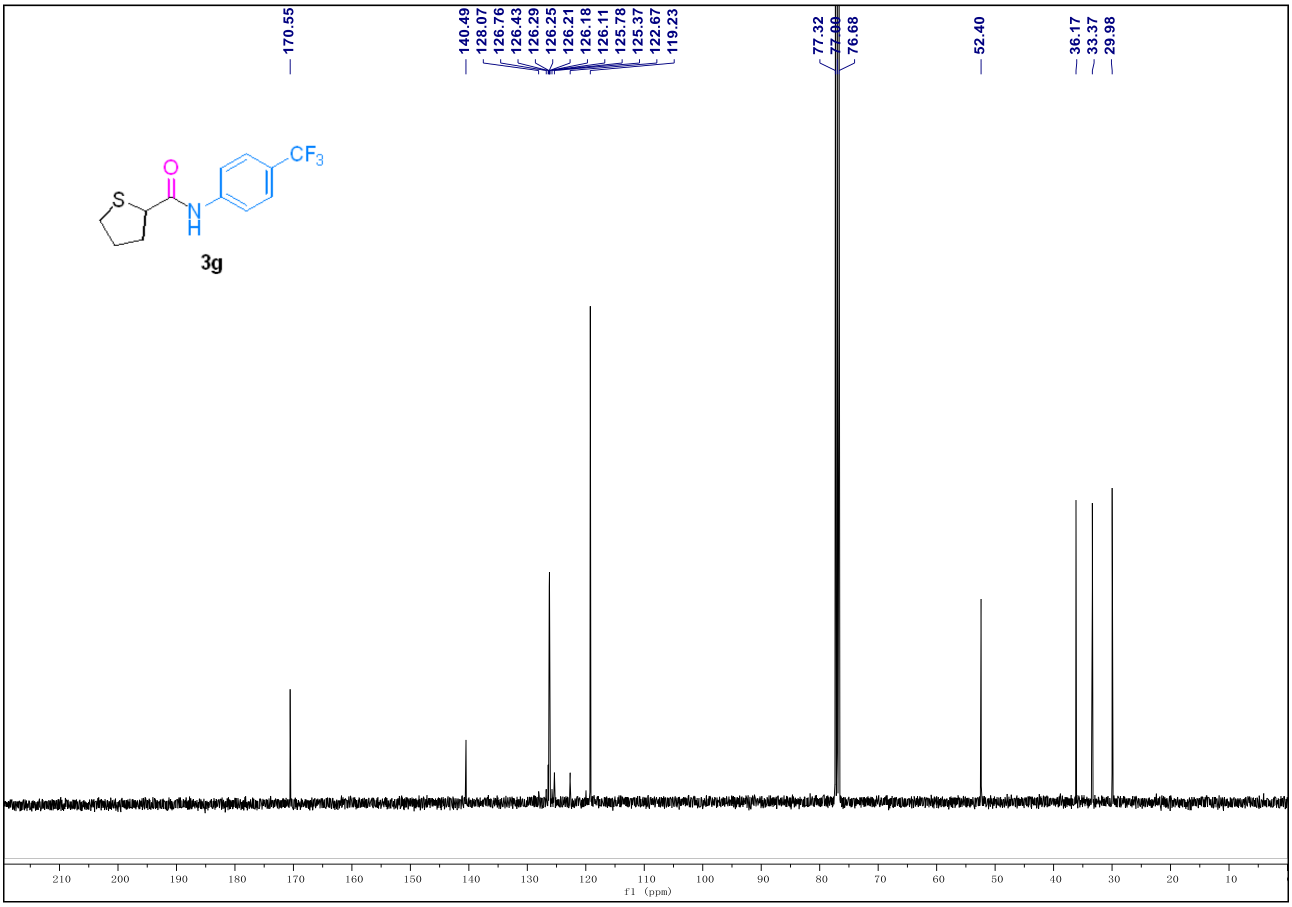

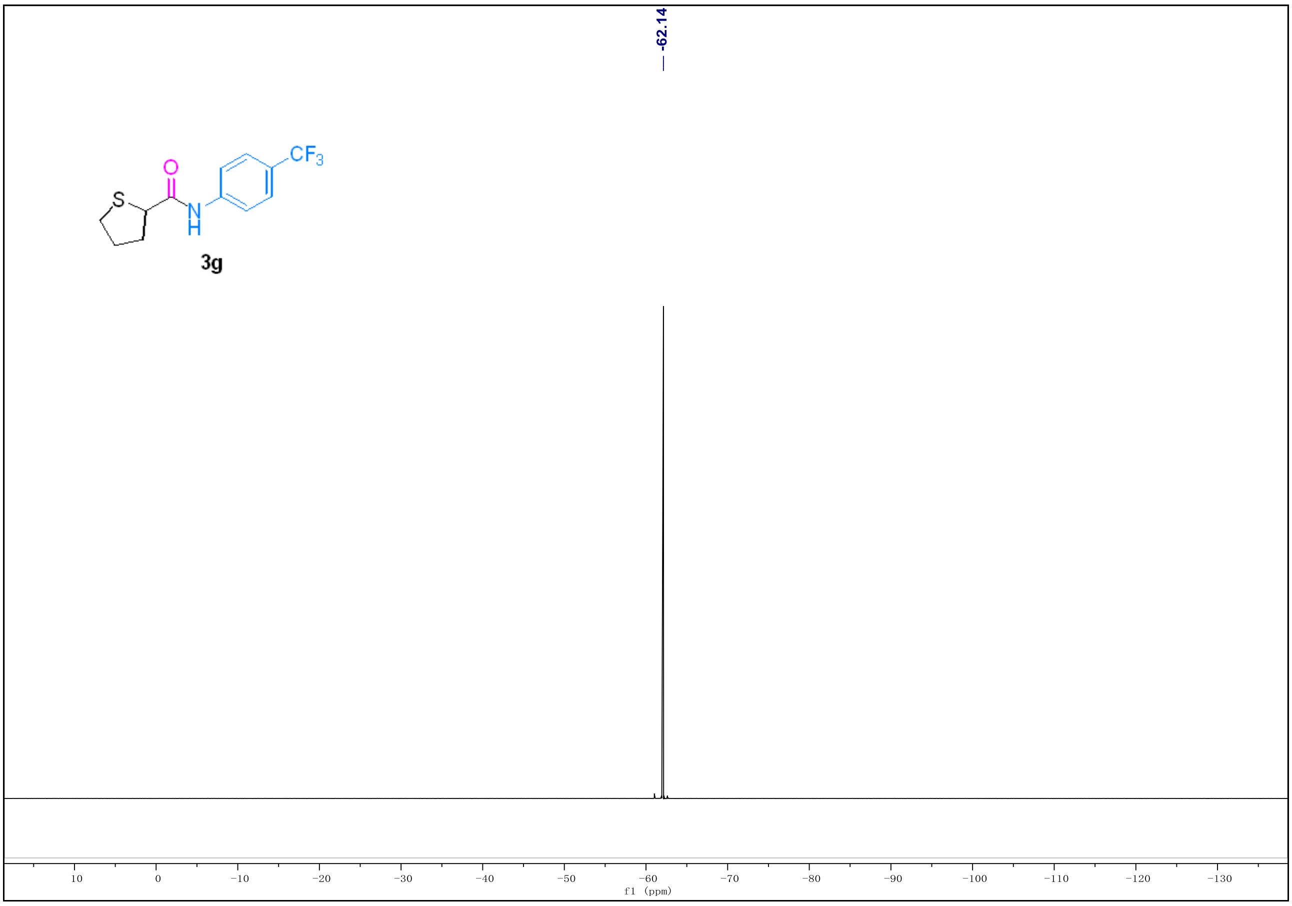
-62.14
S
O
N
H
CF3
3g
10
0
−10
−20
−30
−40
−50
−60
−70
−80
−90
−100
−110
−120
−130
f1 (ppm)

^{1}H NMR (400 MHz, CDCl$_3$) and ^{13}C NMR (101 MHz, CDCl$_3$) spectrum of 3h

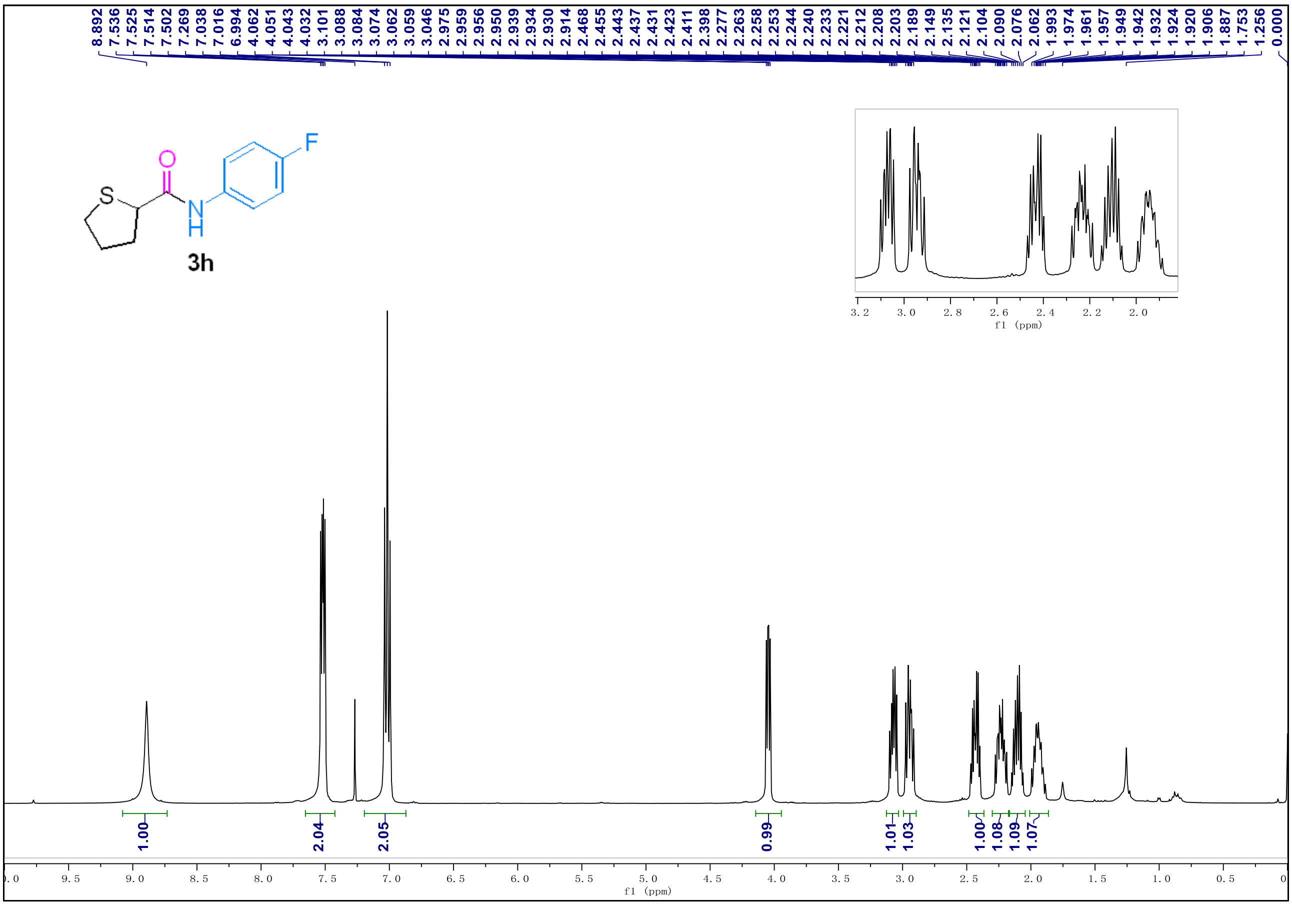

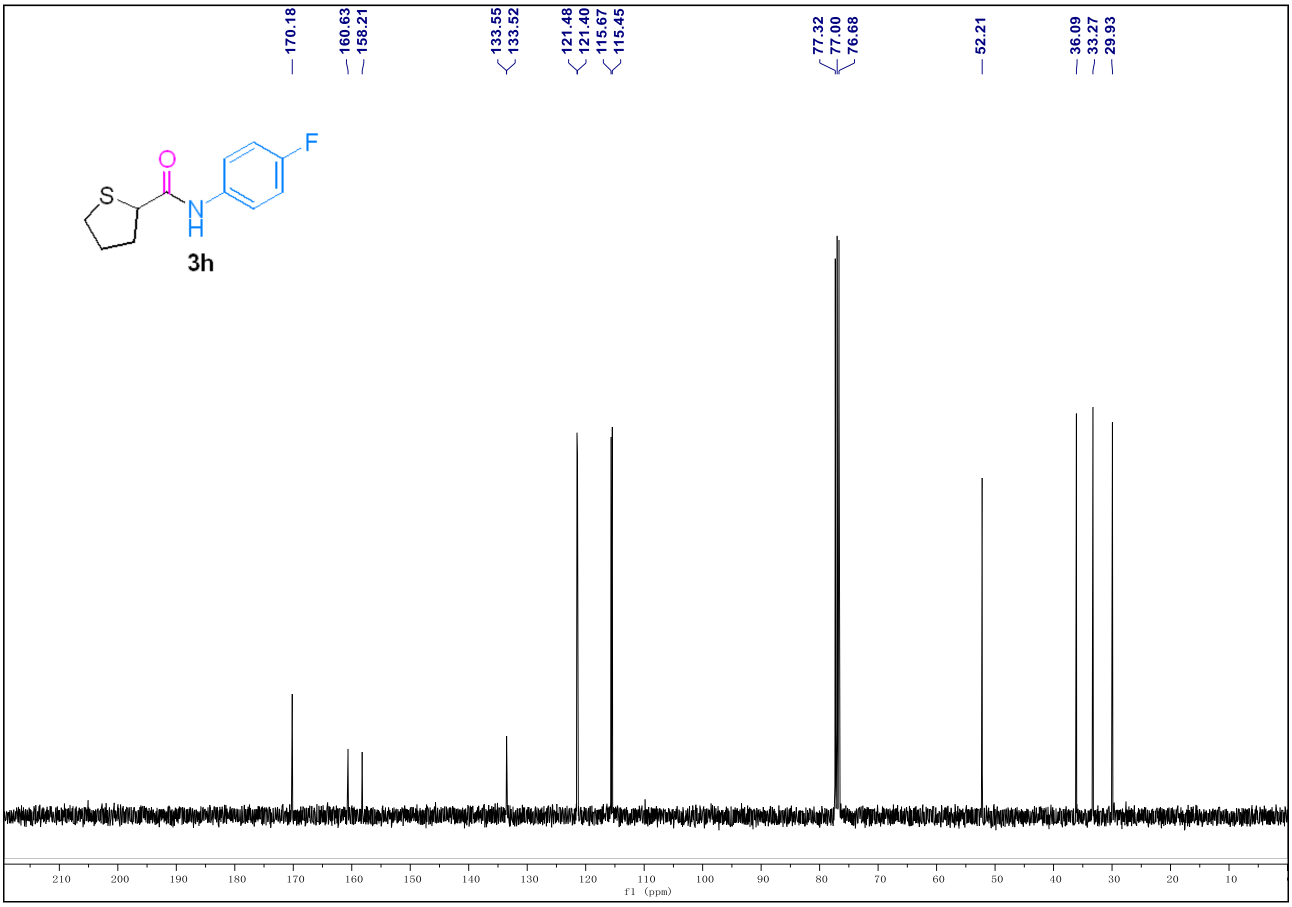

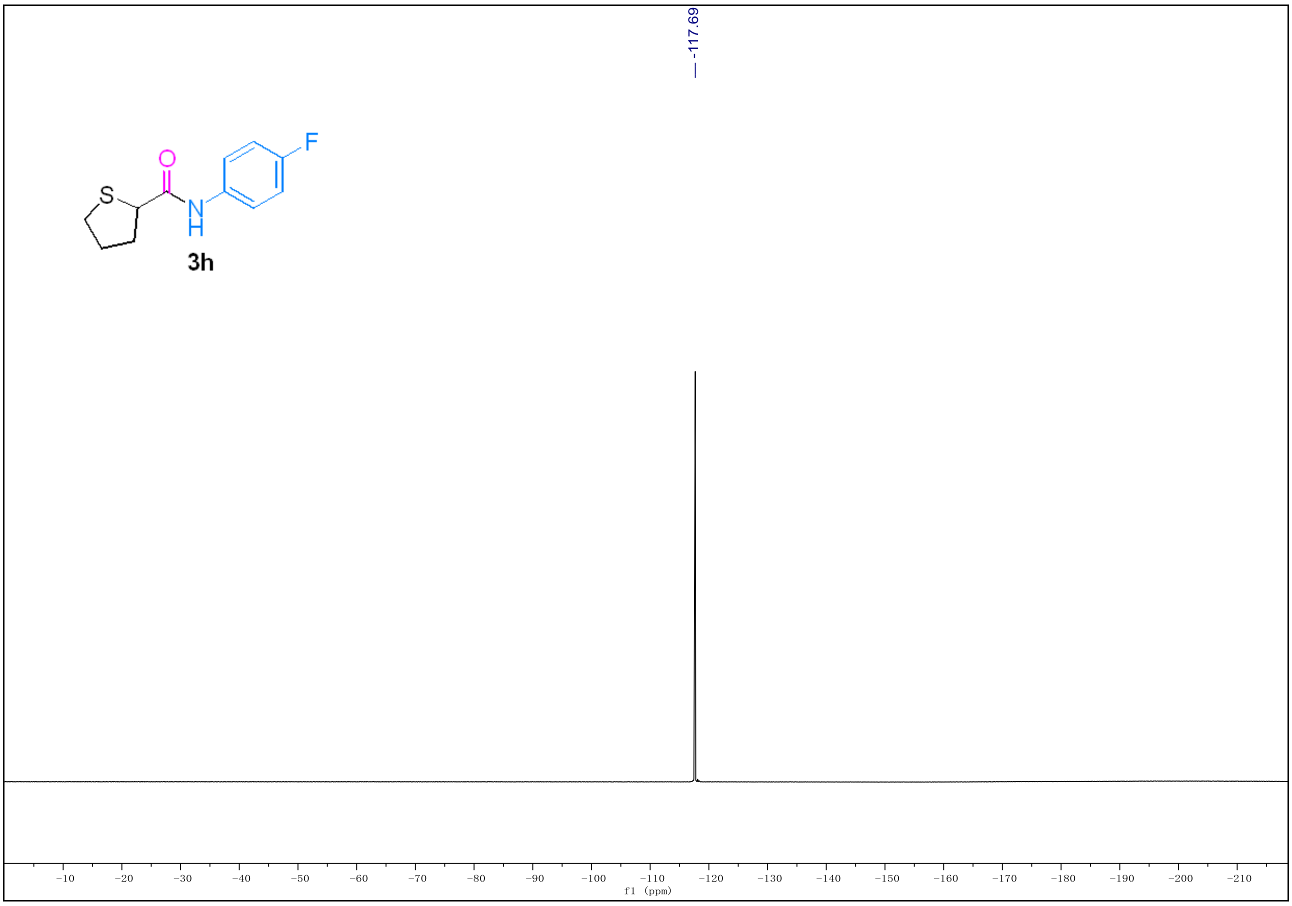

-117.69
3h
-10
-20
-30
-40
-50
-60
-70
-80
-90
-100
-110
-120
-130
-140
-150
-160
-170
-180
-190
-200
-210
f1 (ppm)

^{1}H NMR (400 MHz, $CDCl_3$) and ^{13}C NMR (101 MHz, $CDCl_3$) spectrum of 3i

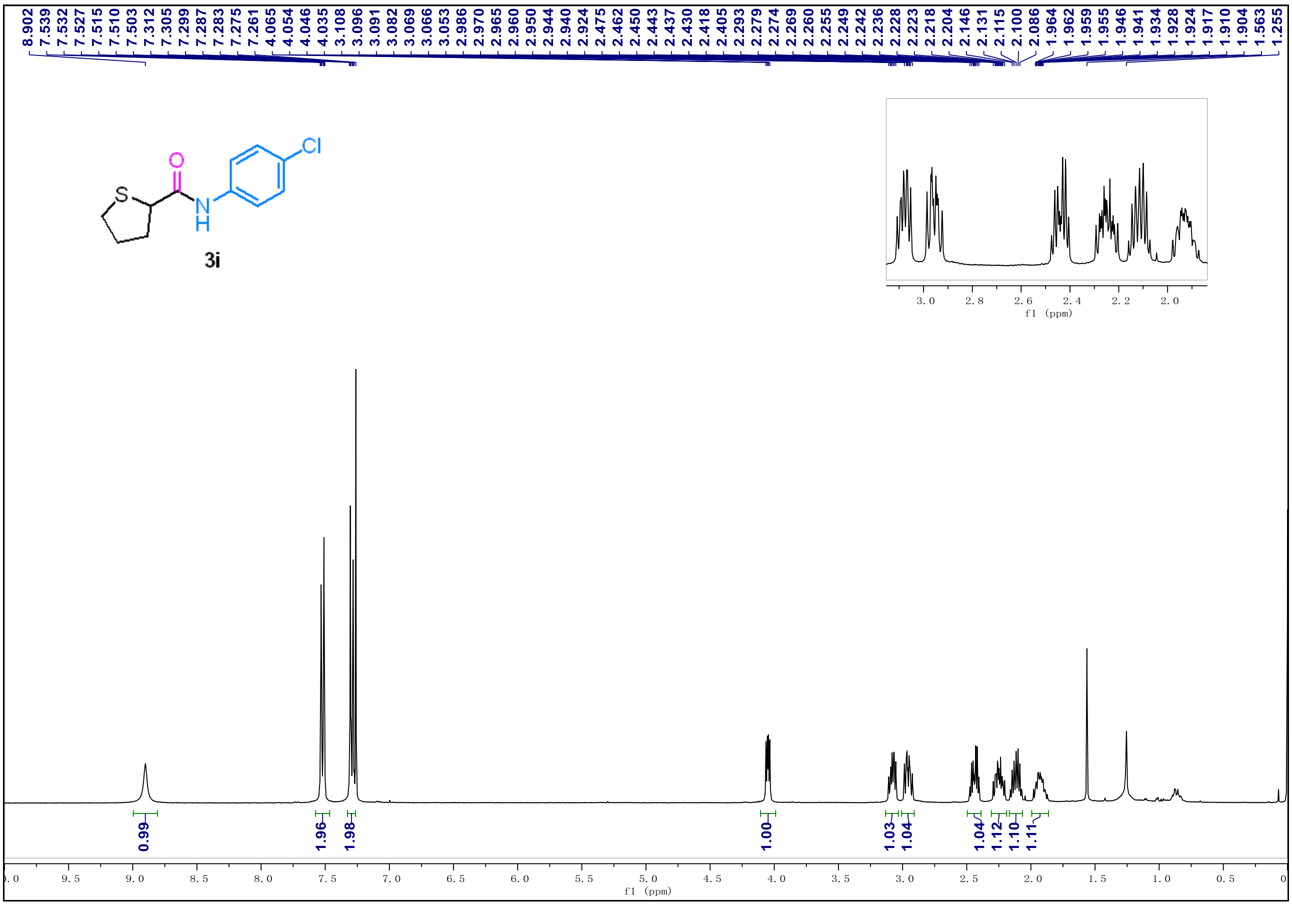

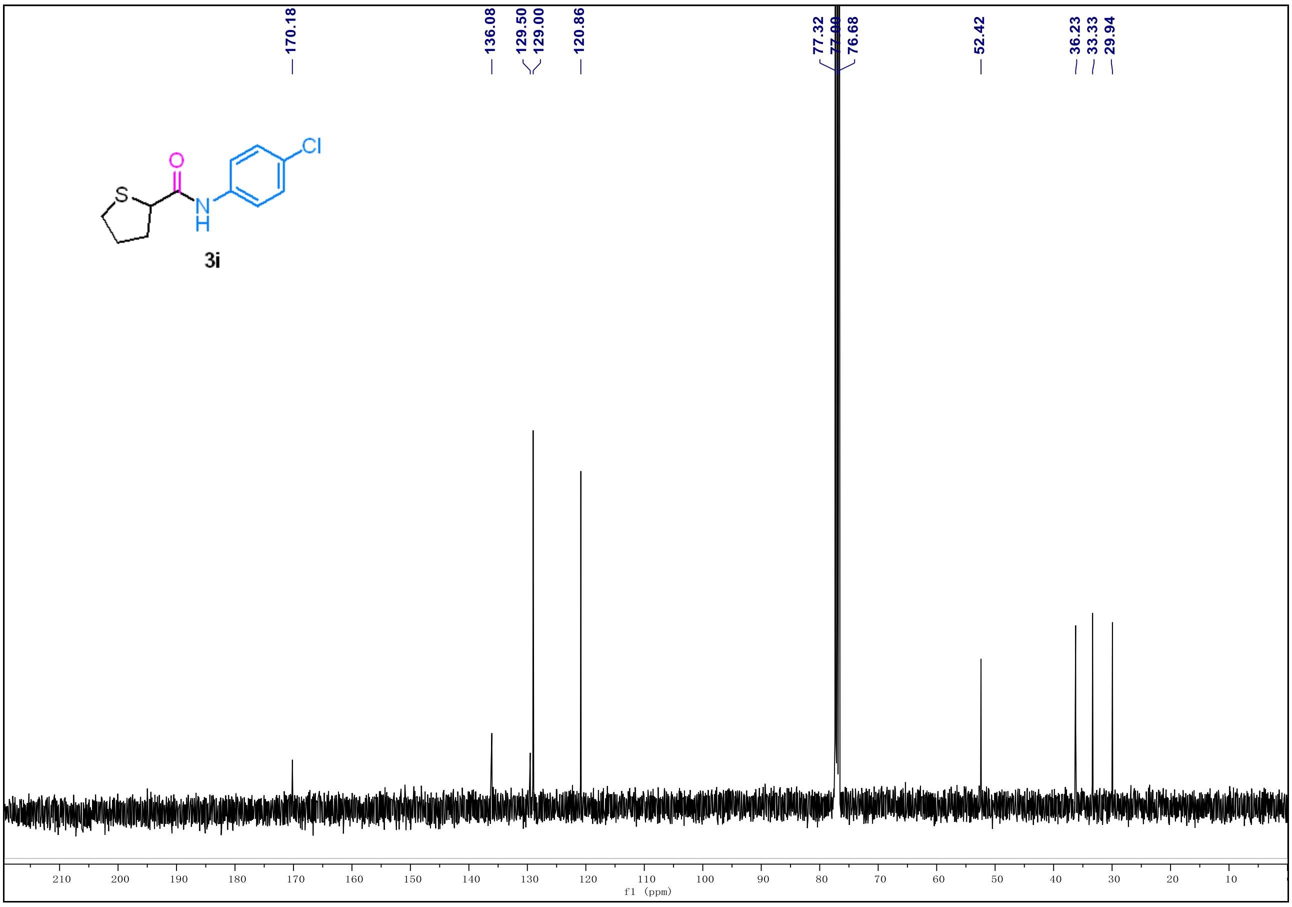

^{1}H NMR (400 MHz, CDCl$_3$) and ^{13}C NMR (101 MHz, CDCl$_3$) spectrum of 3j

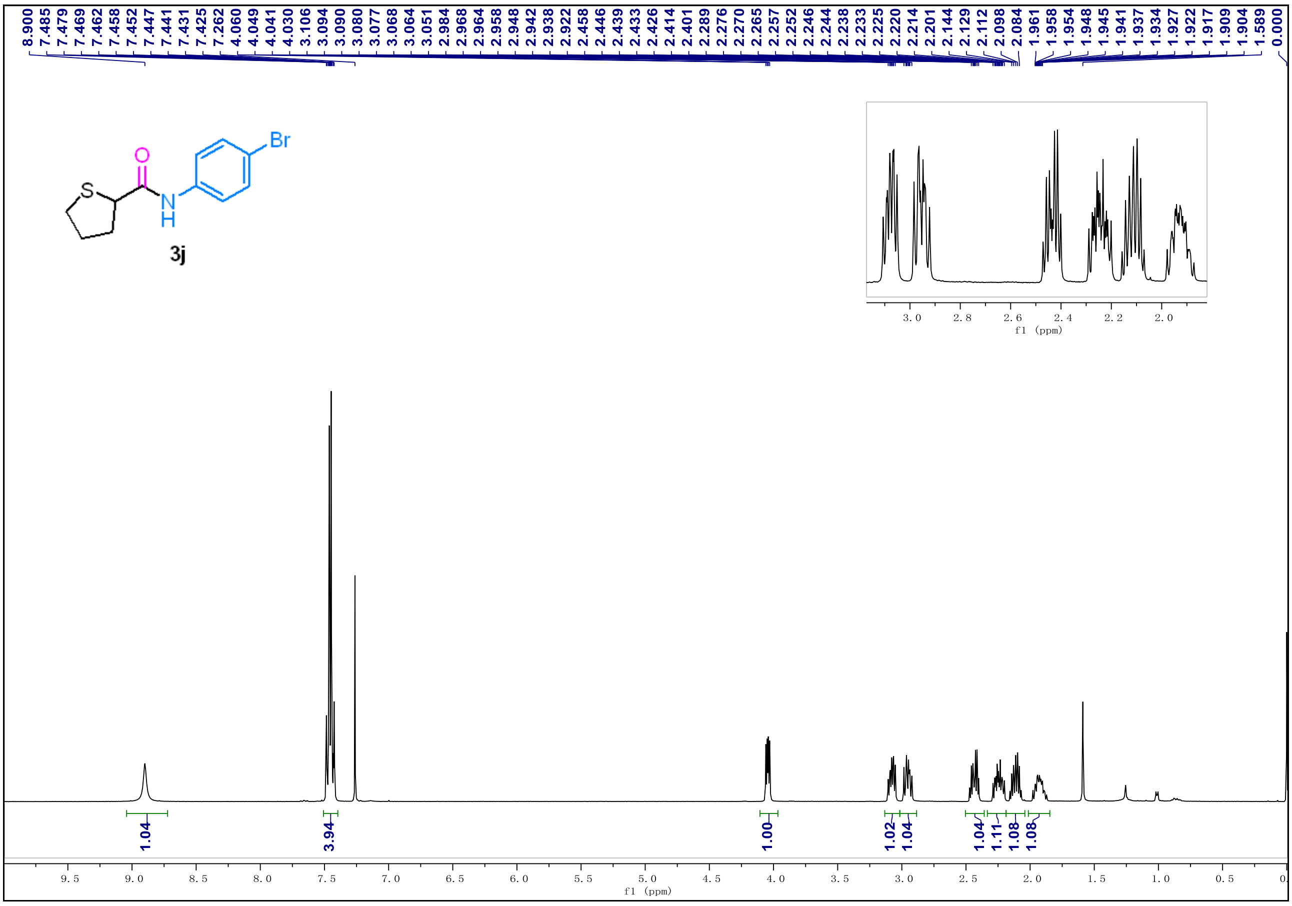

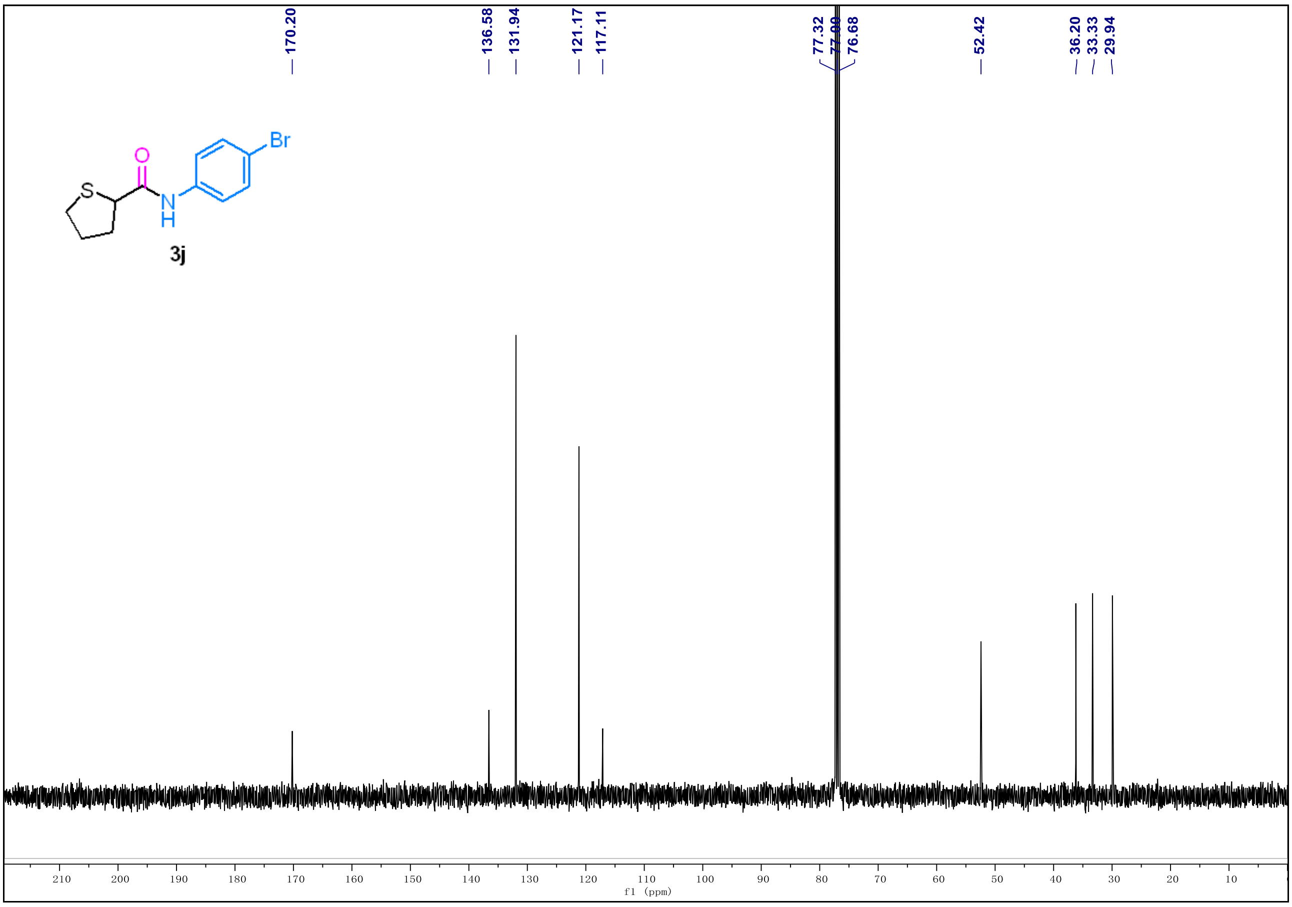

^{1}H NMR (400 MHz, $CDCl_3$) and ^{13}C NMR (101 MHz, $CDCl_3$) spectrum of 3k

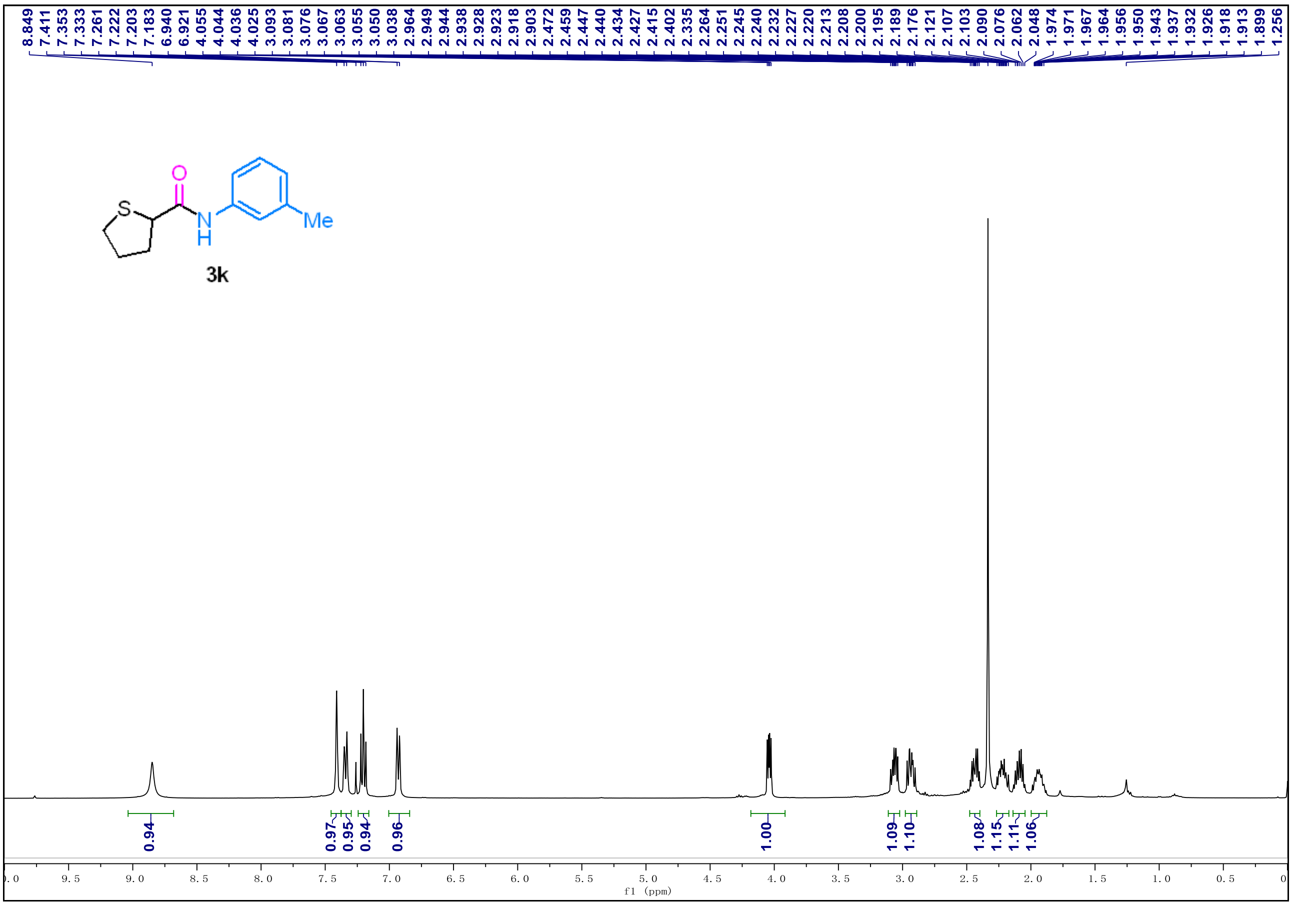

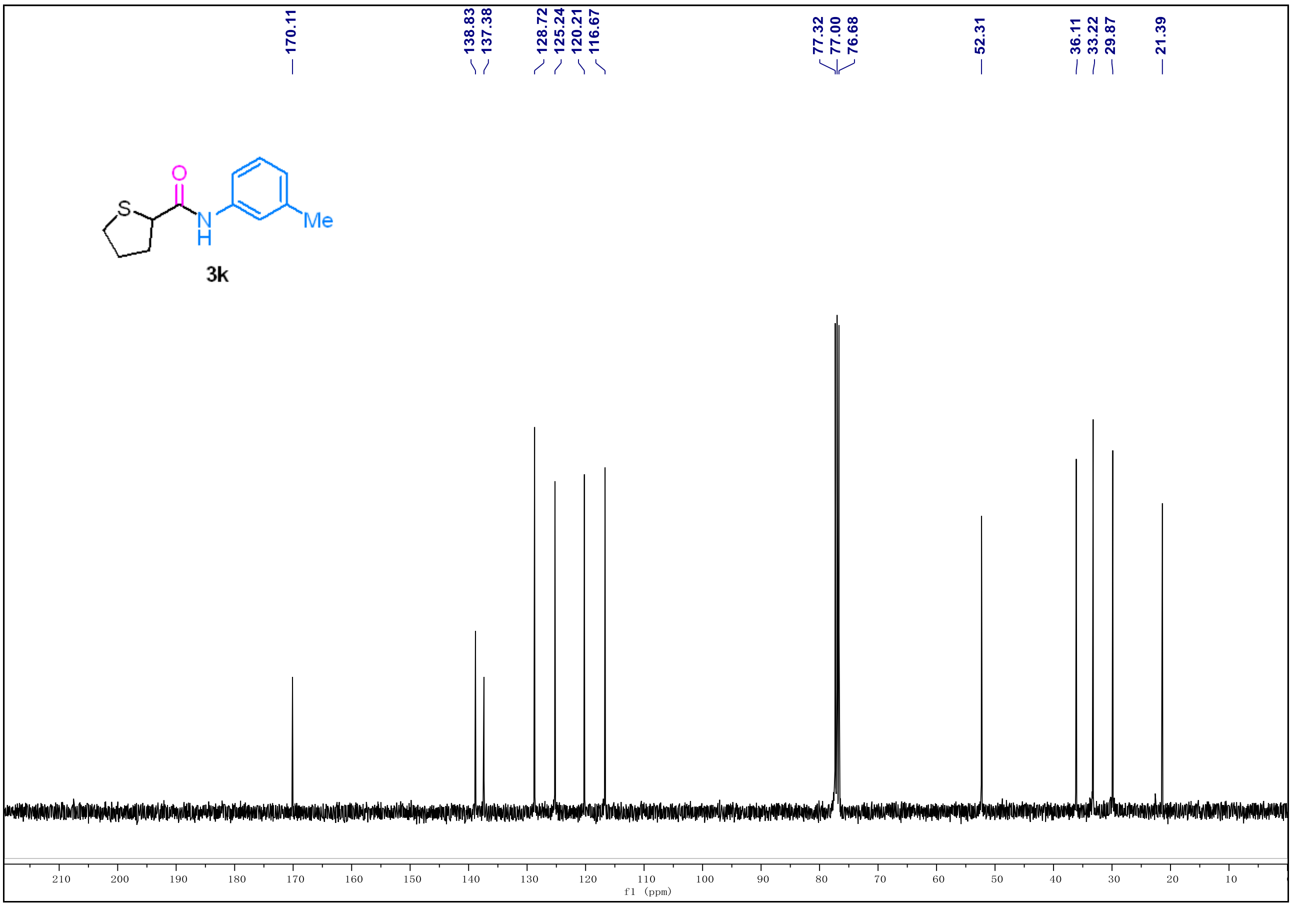

^{1}H NMR (400 MHz, CDCl$_3$) and ^{13}C NMR (101 MHz, CDCl$_3$) spectrum of 3l

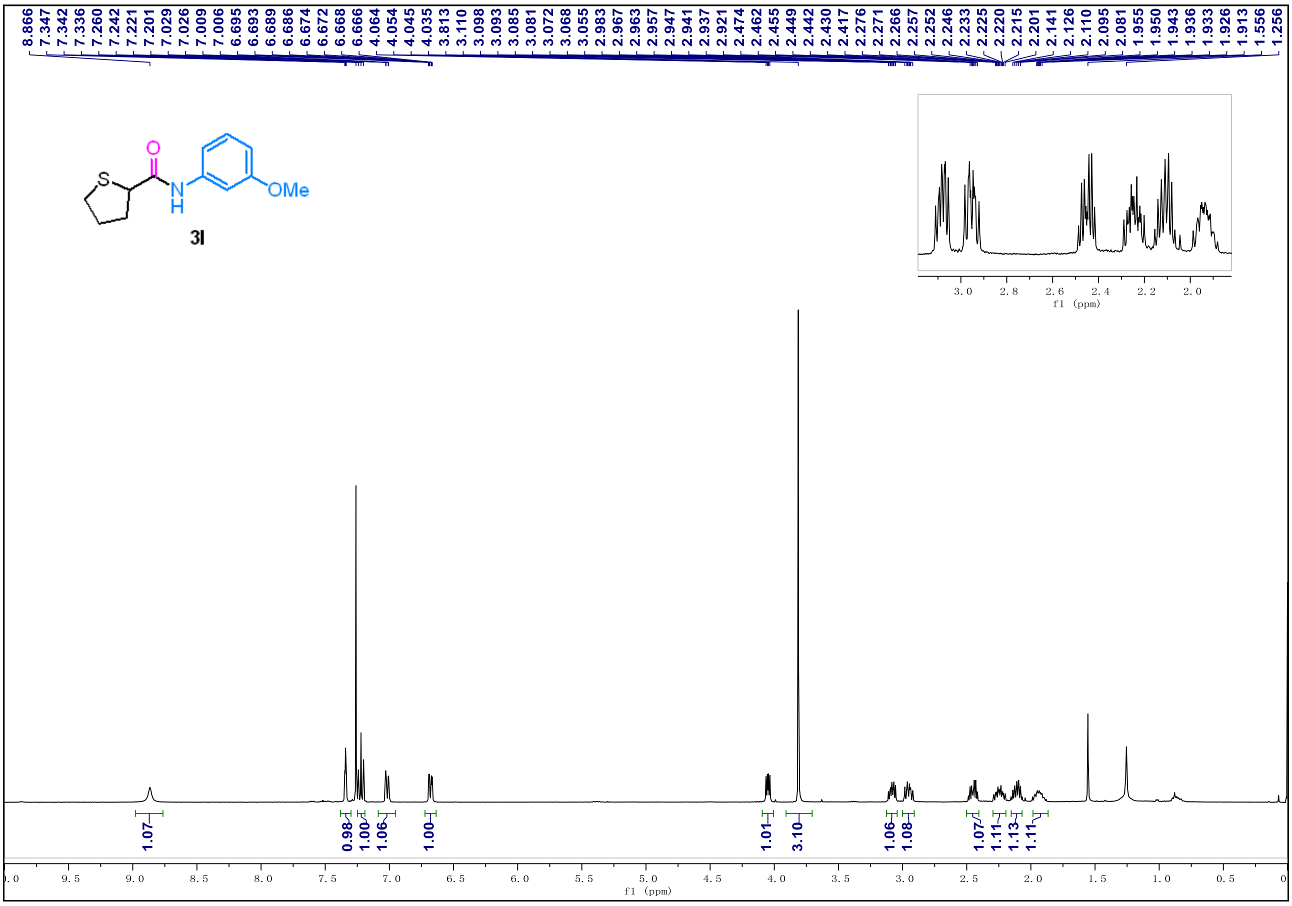

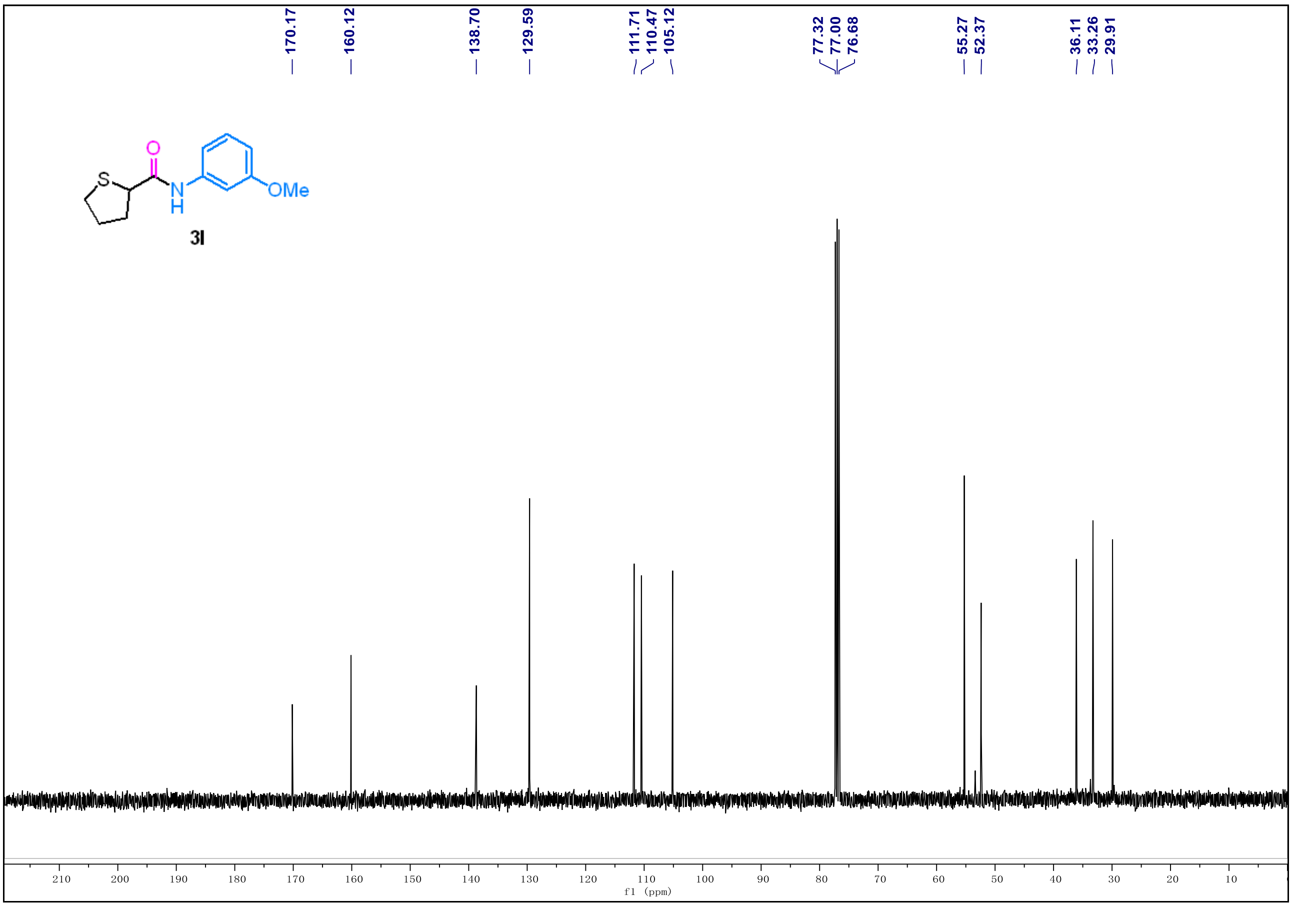

^{1}H NMR (400 MHz, CDCl3) and ^{13}C NMR (101 MHz, CDCl3) spectrum of 3m

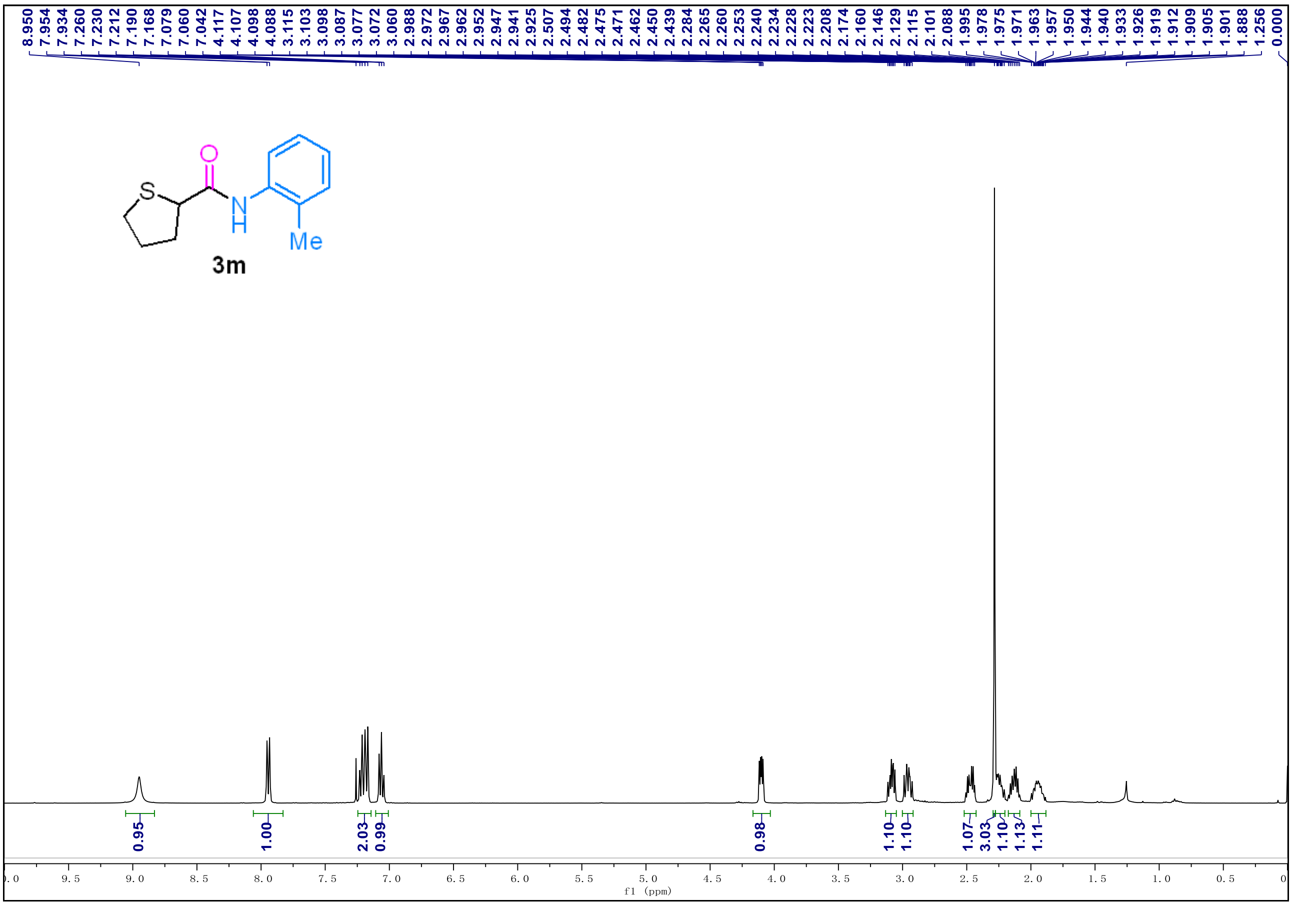

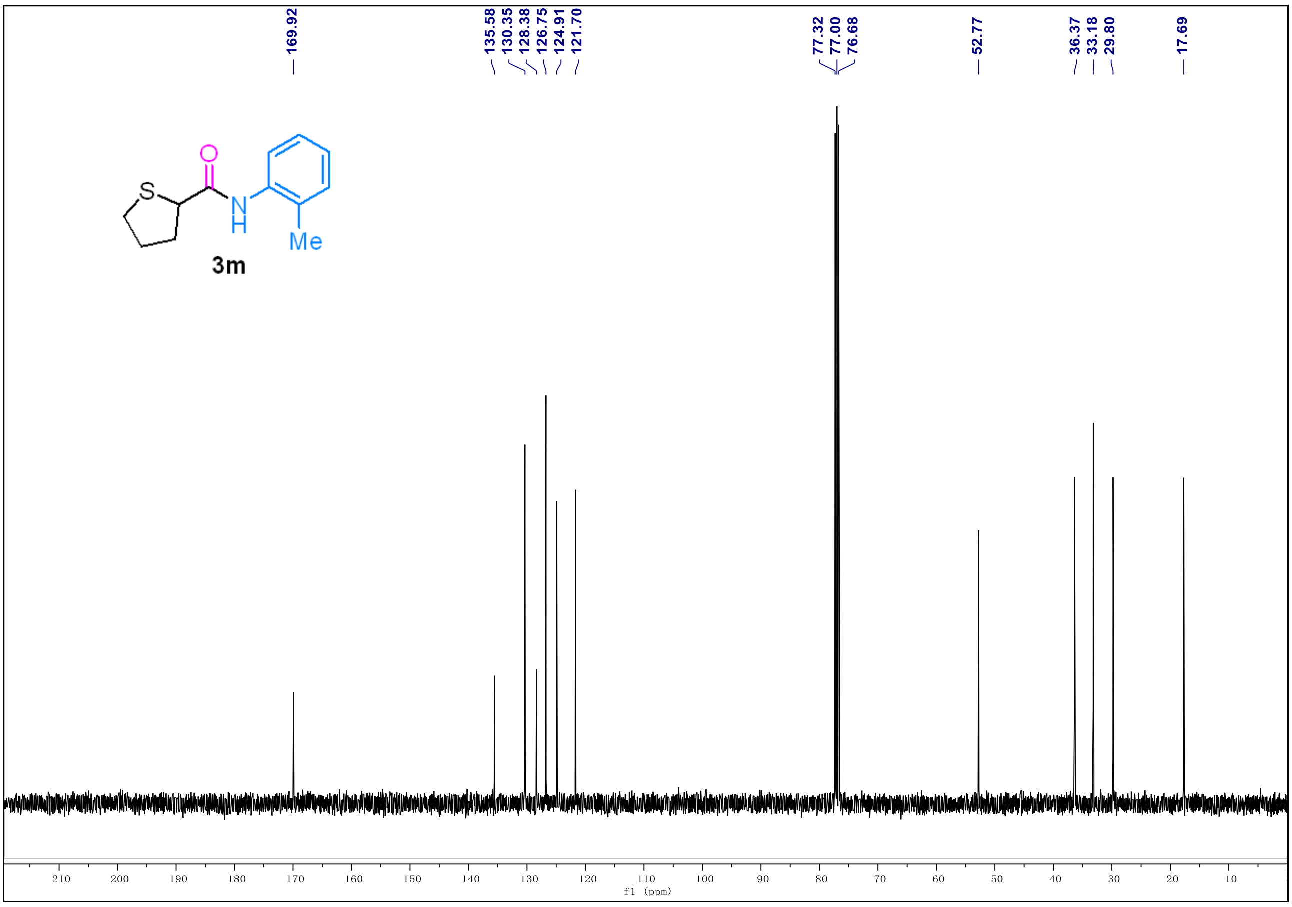

^{1}H NMR (400 MHz, CDCl$_3$) and ^{13}C NMR (101 MHz, CDCl$_3$) spectrum of 3n

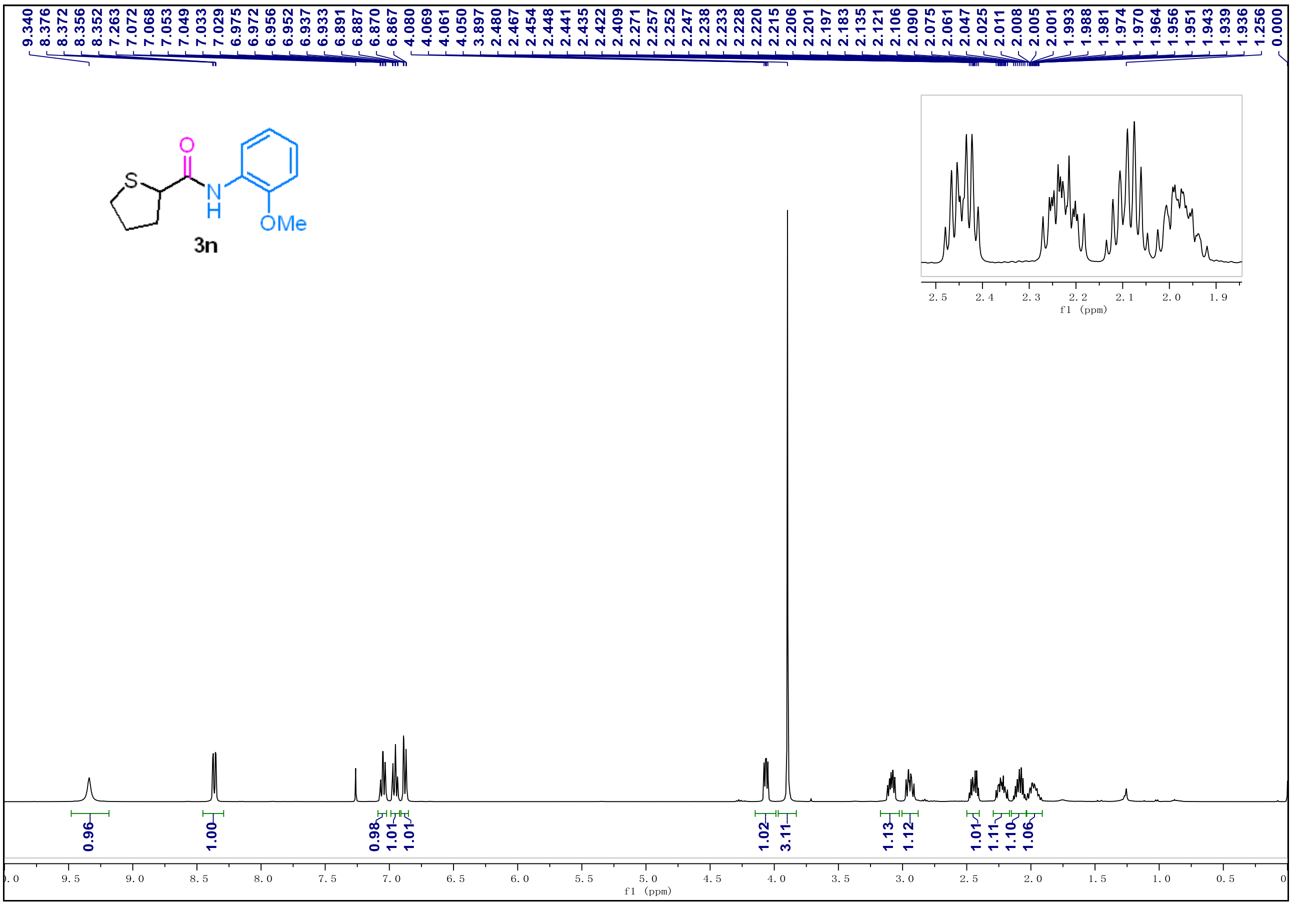

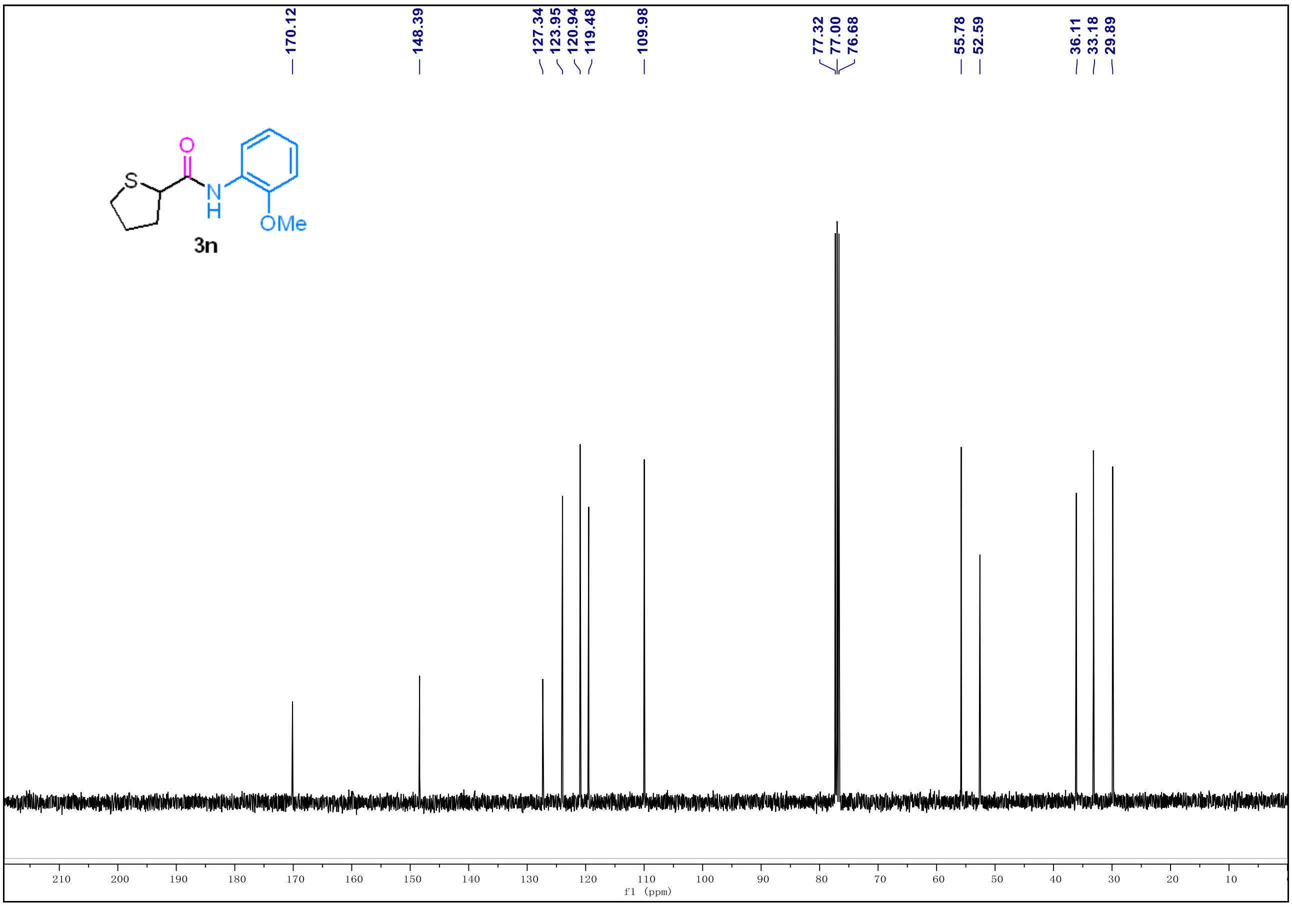

^{1}H NMR (400 MHz, CDCl3) and ^{13}C NMR (101 MHz, CDCl3) spectrum of 3o

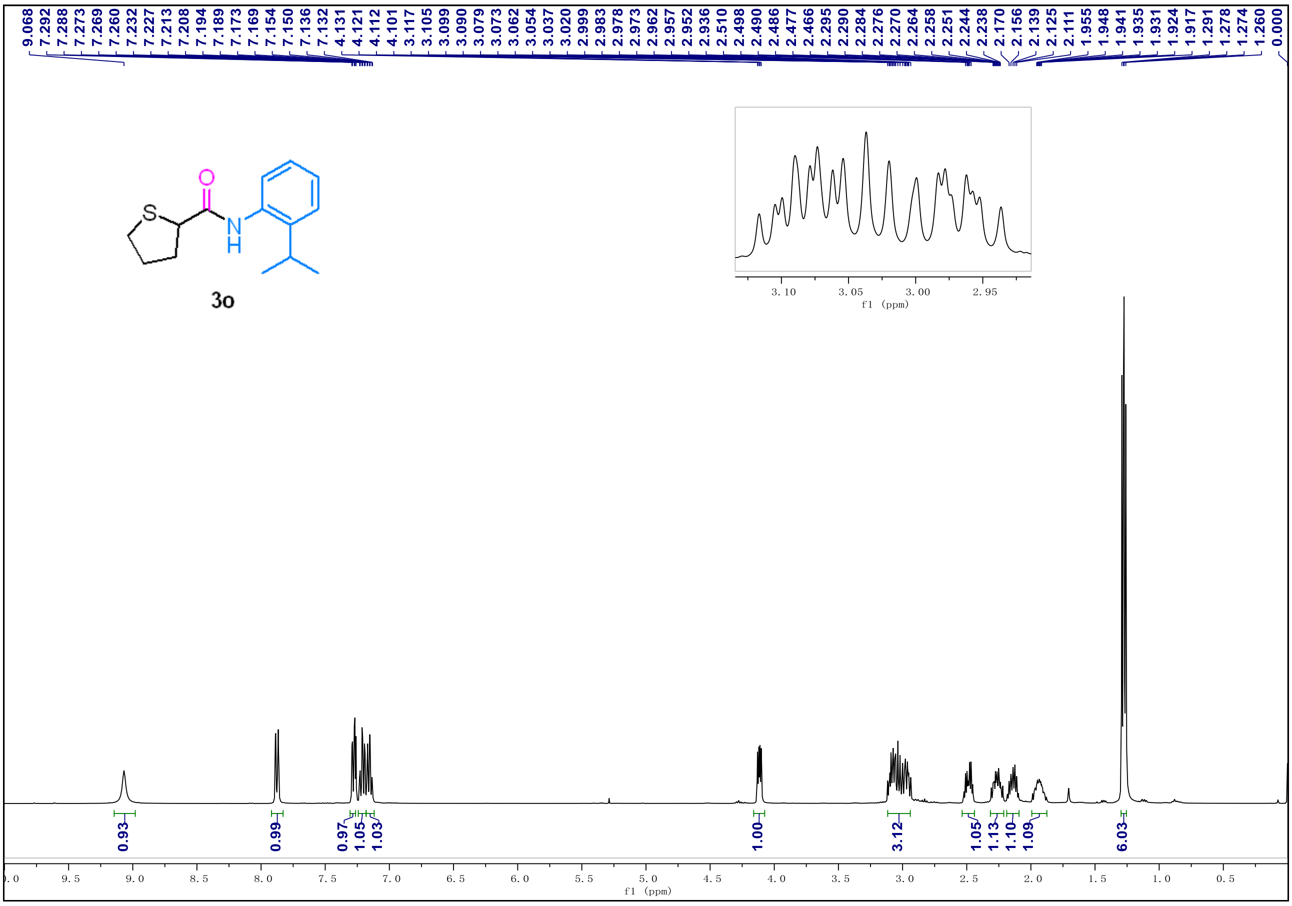

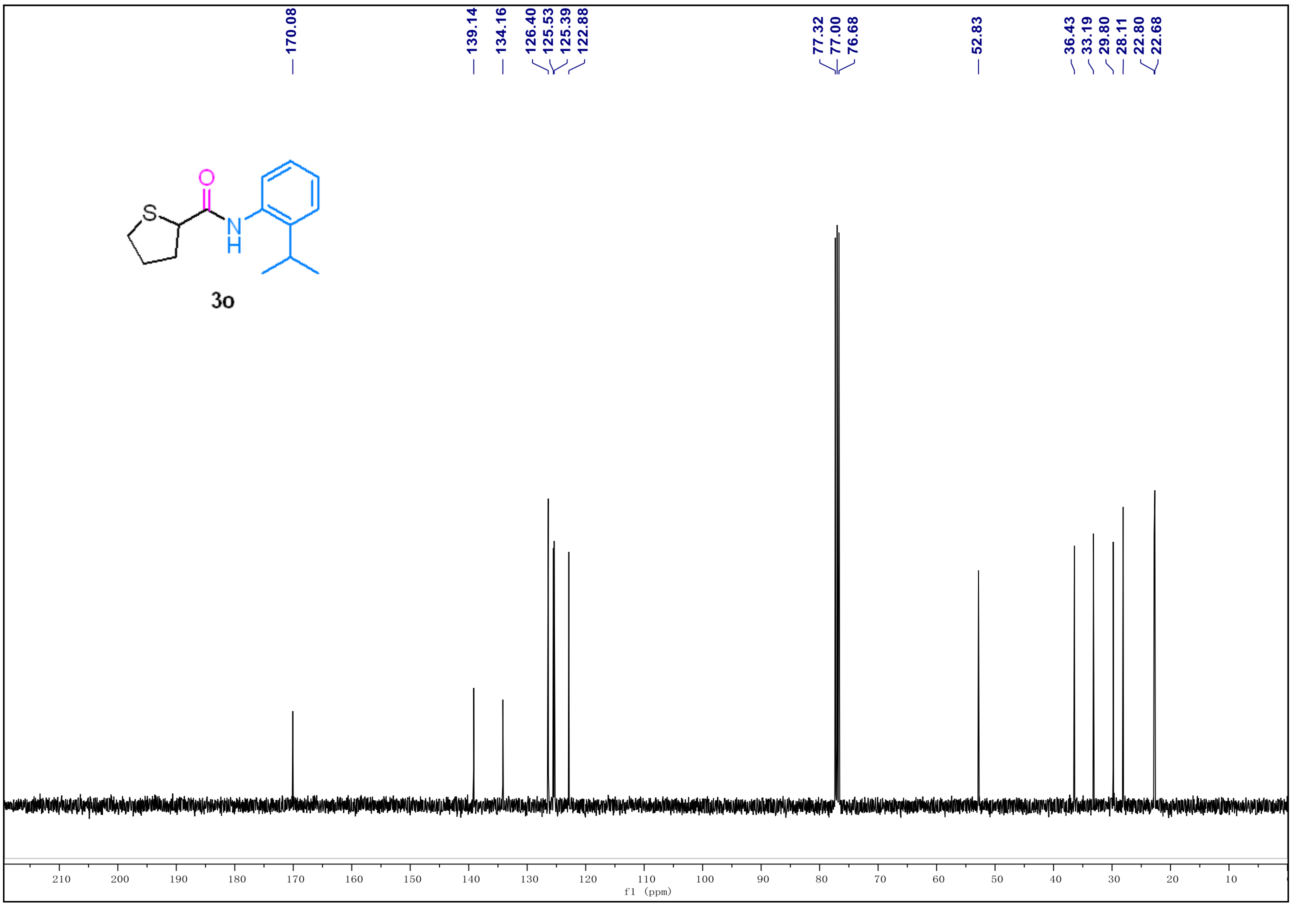

^{1}H NMR (400 MHz, CDCl3) and ^{13}C NMR (101 MHz, CDCl3) spectrum of 3p

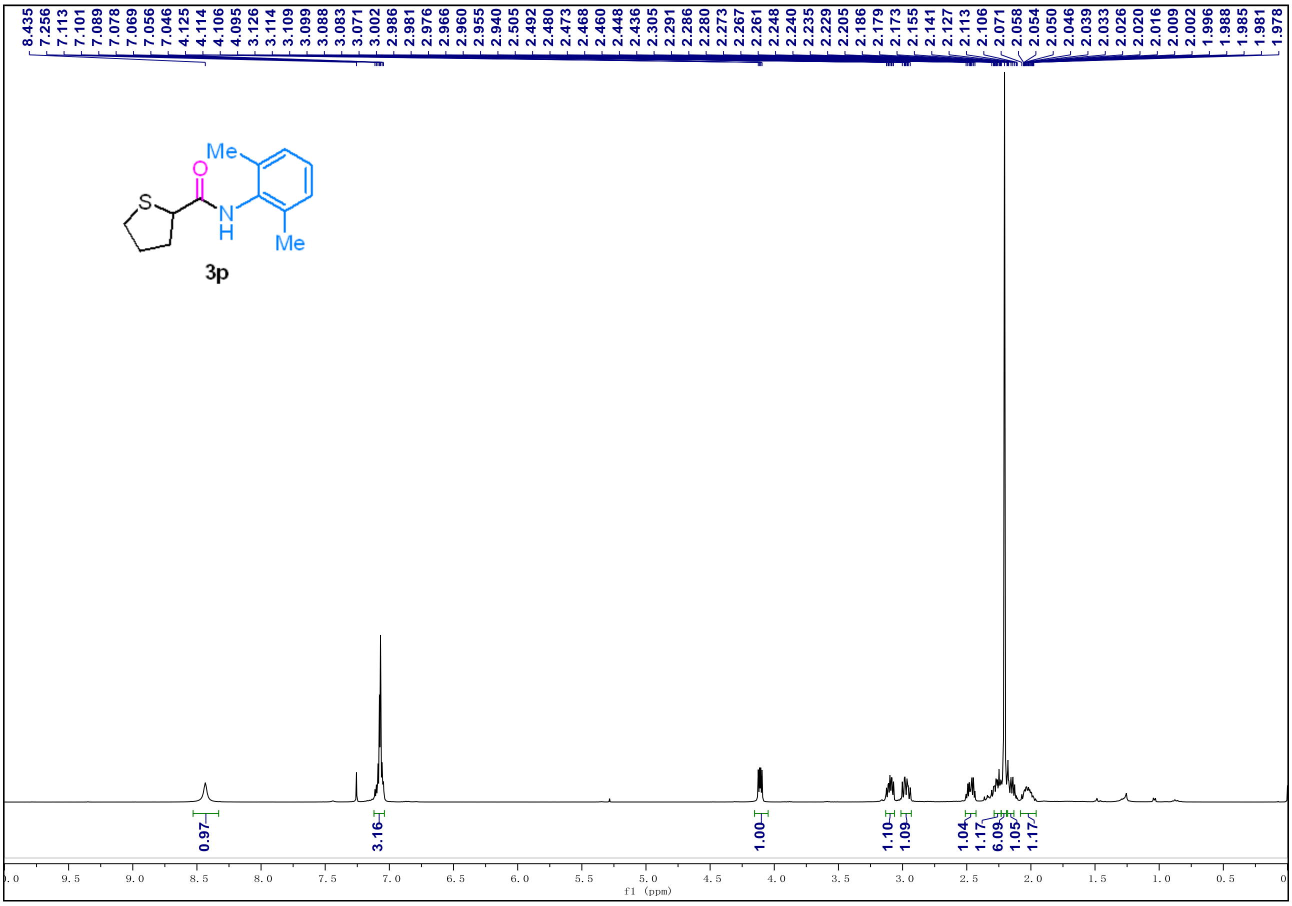

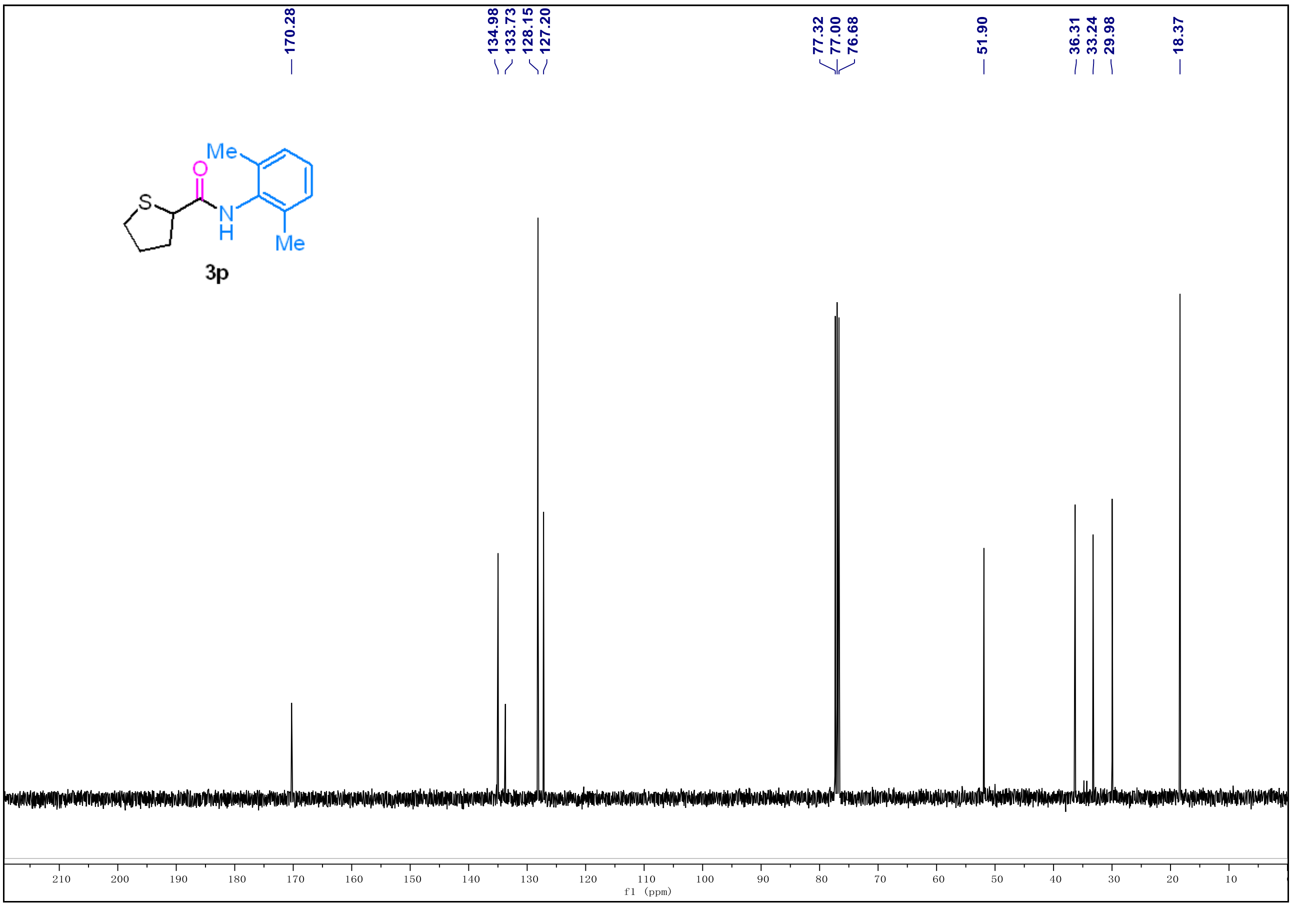

^{1}H NMR (400 MHz, $CDCl_3$) and ^{13}C NMR (101 MHz, $CDCl_3$) spectrum of 3q

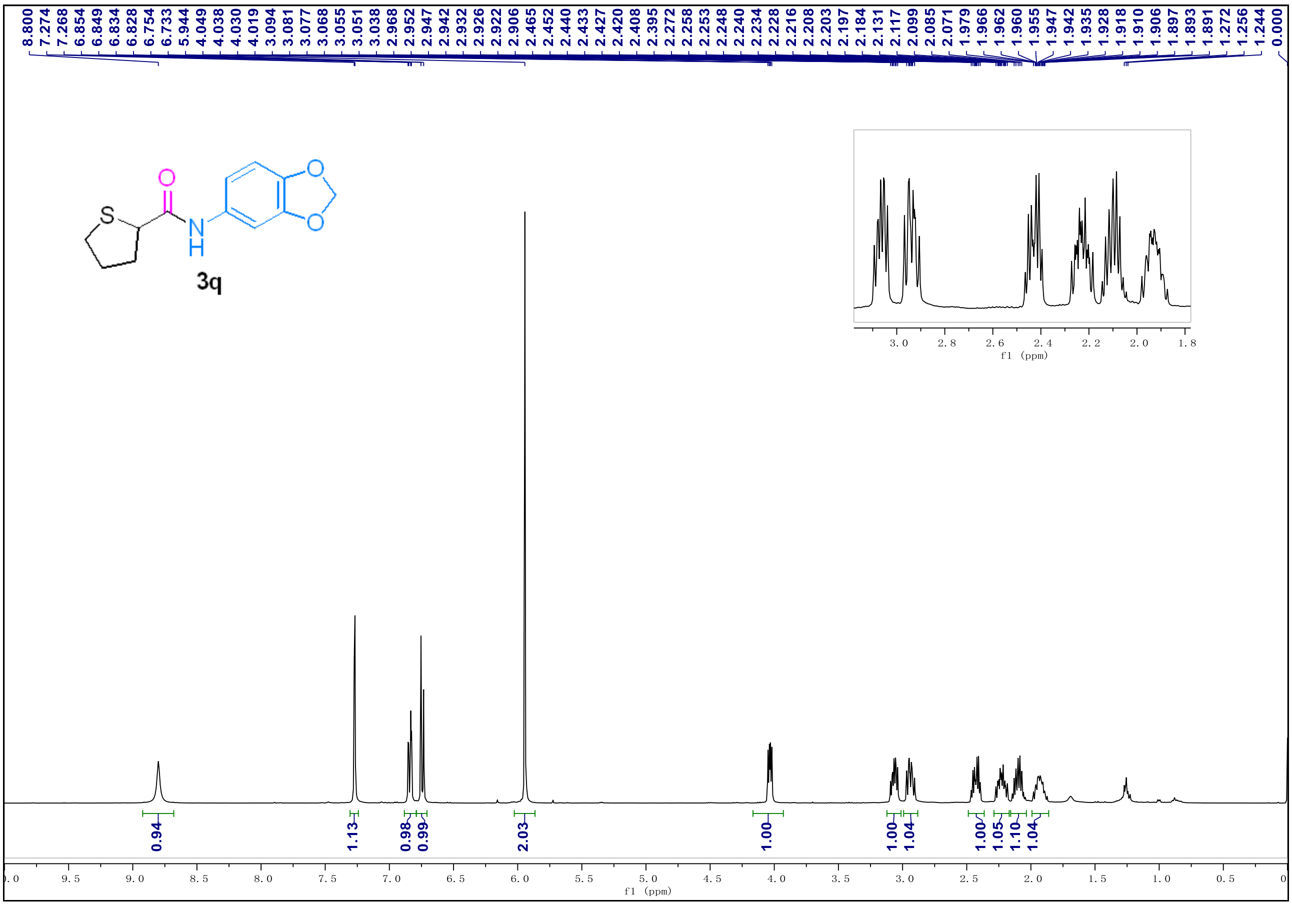

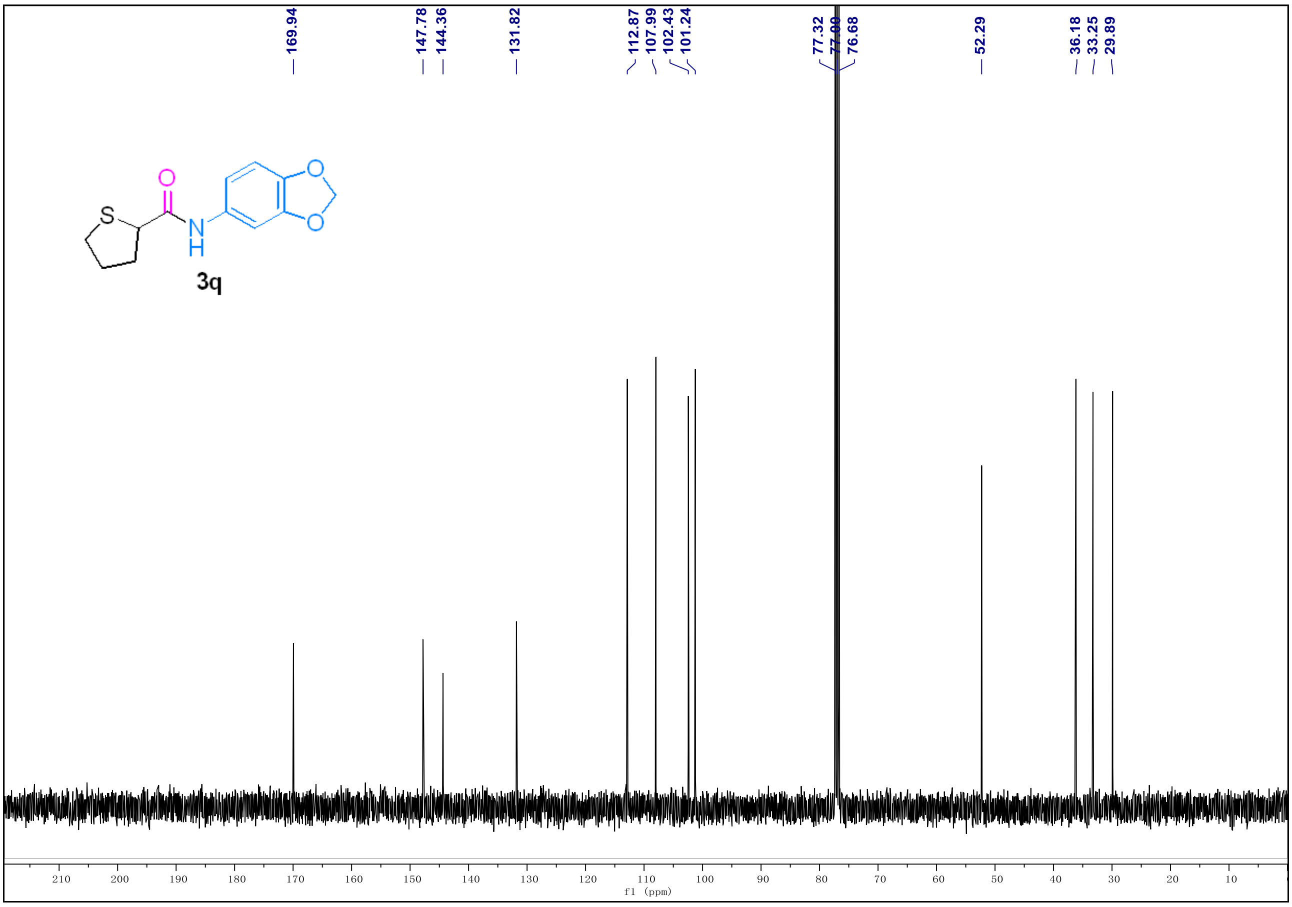

^{1}H NMR (400 MHz, $CDCl_3$) and ^{13}C NMR (101 MHz, $CDCl_3$) spectrum of 3r

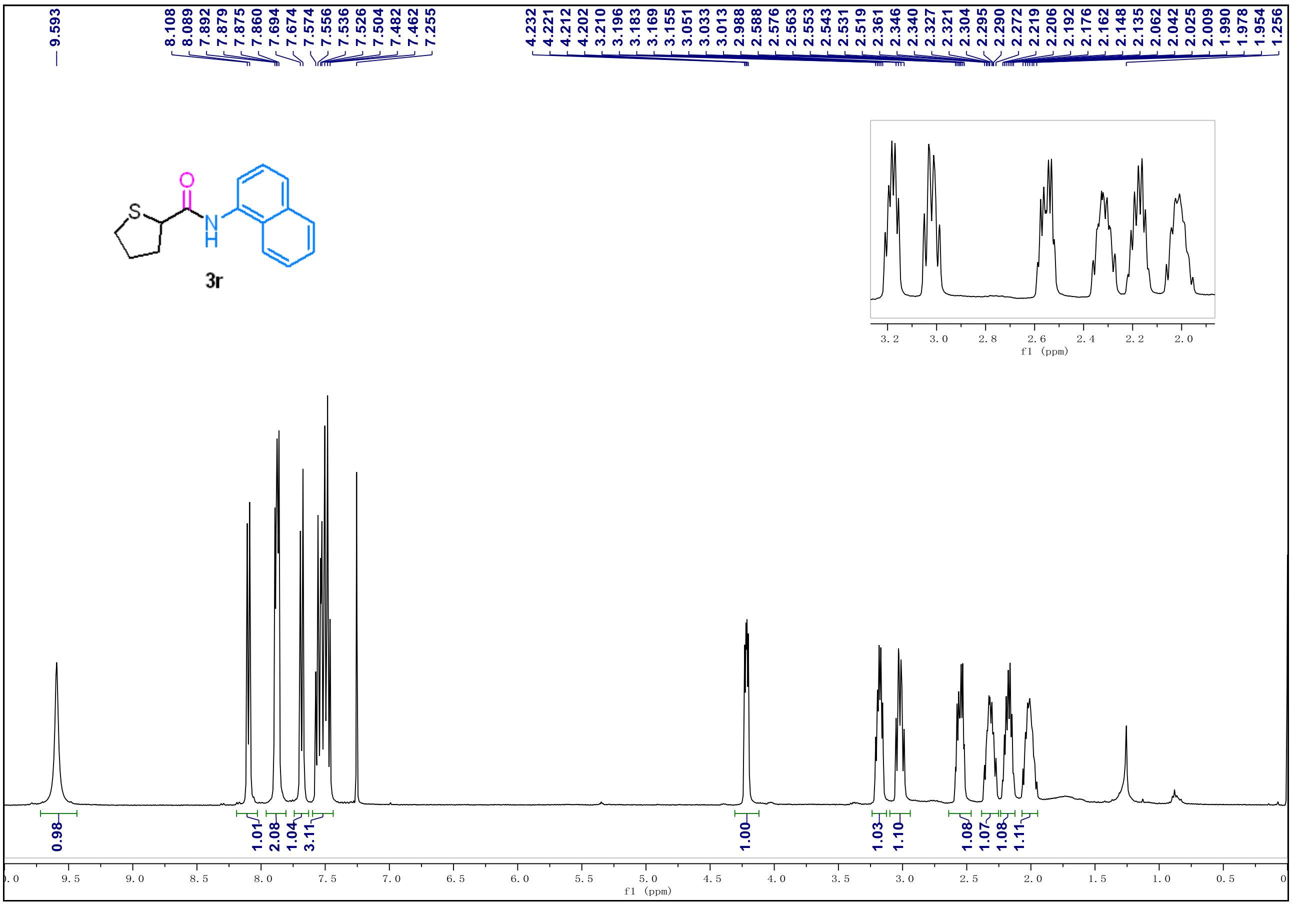

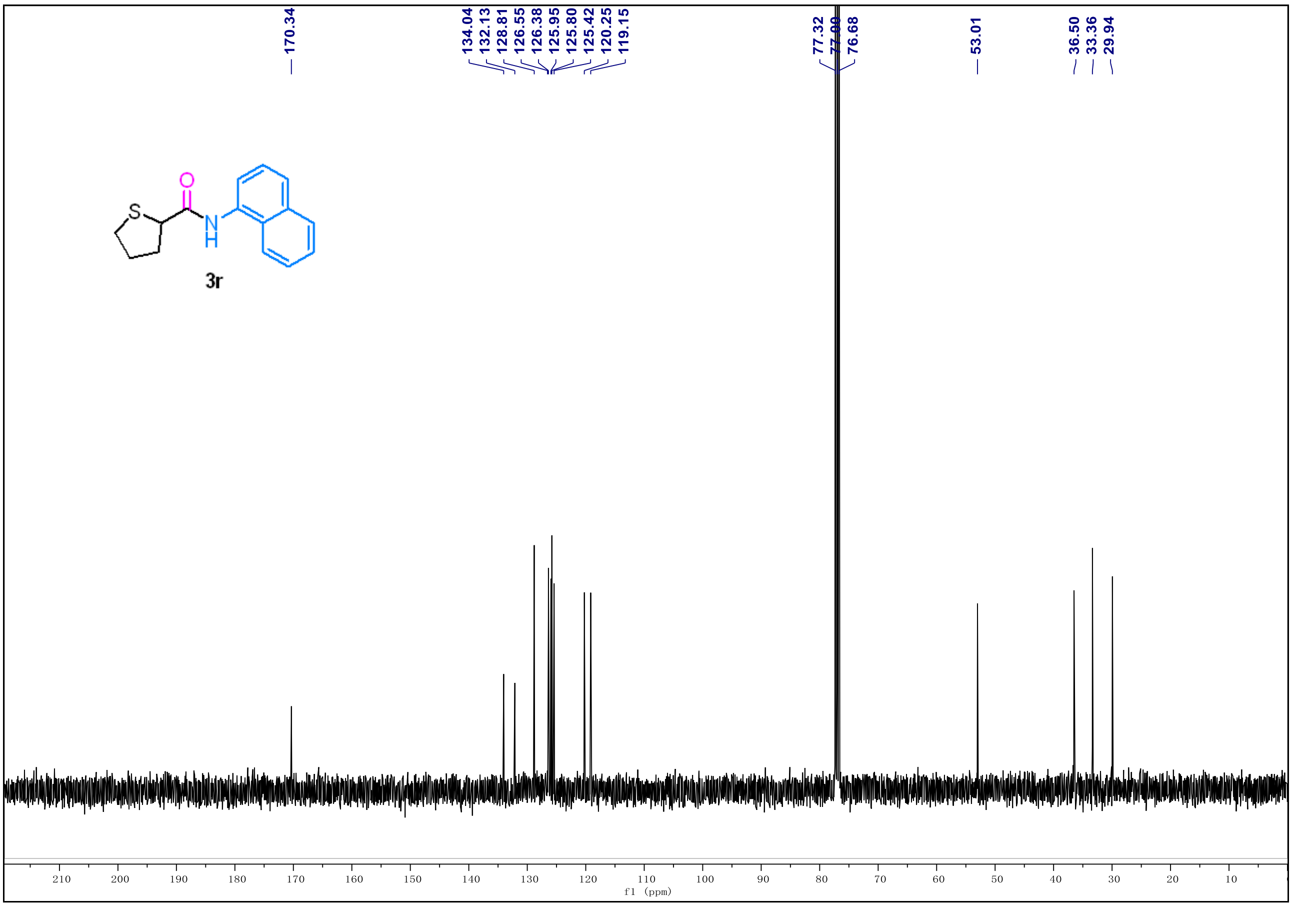

^{1}H NMR (400 MHz, CDCl$_3$) and ^{13}C NMR (101 MHz, CDCl$_3$) spectrum of 3s

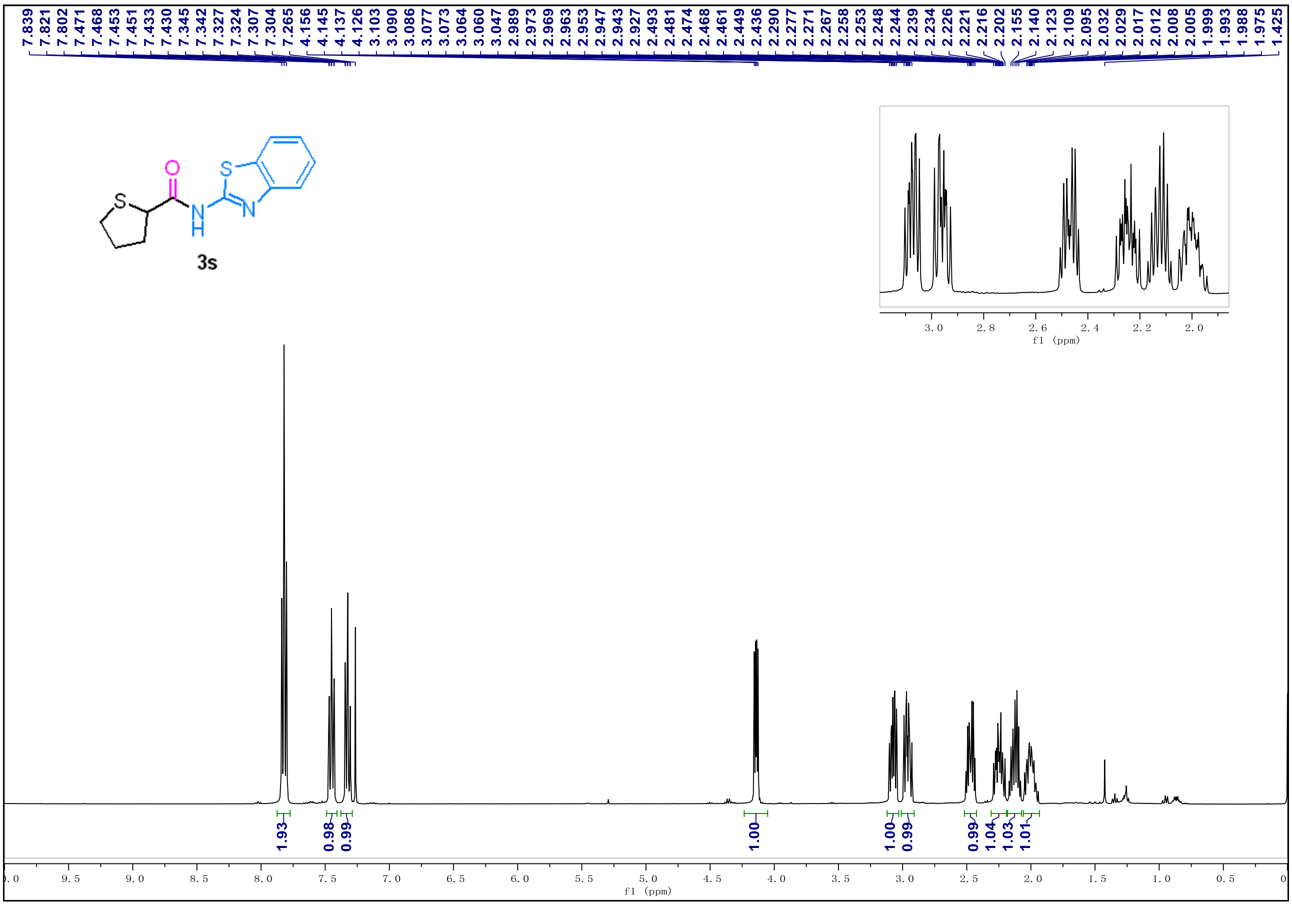

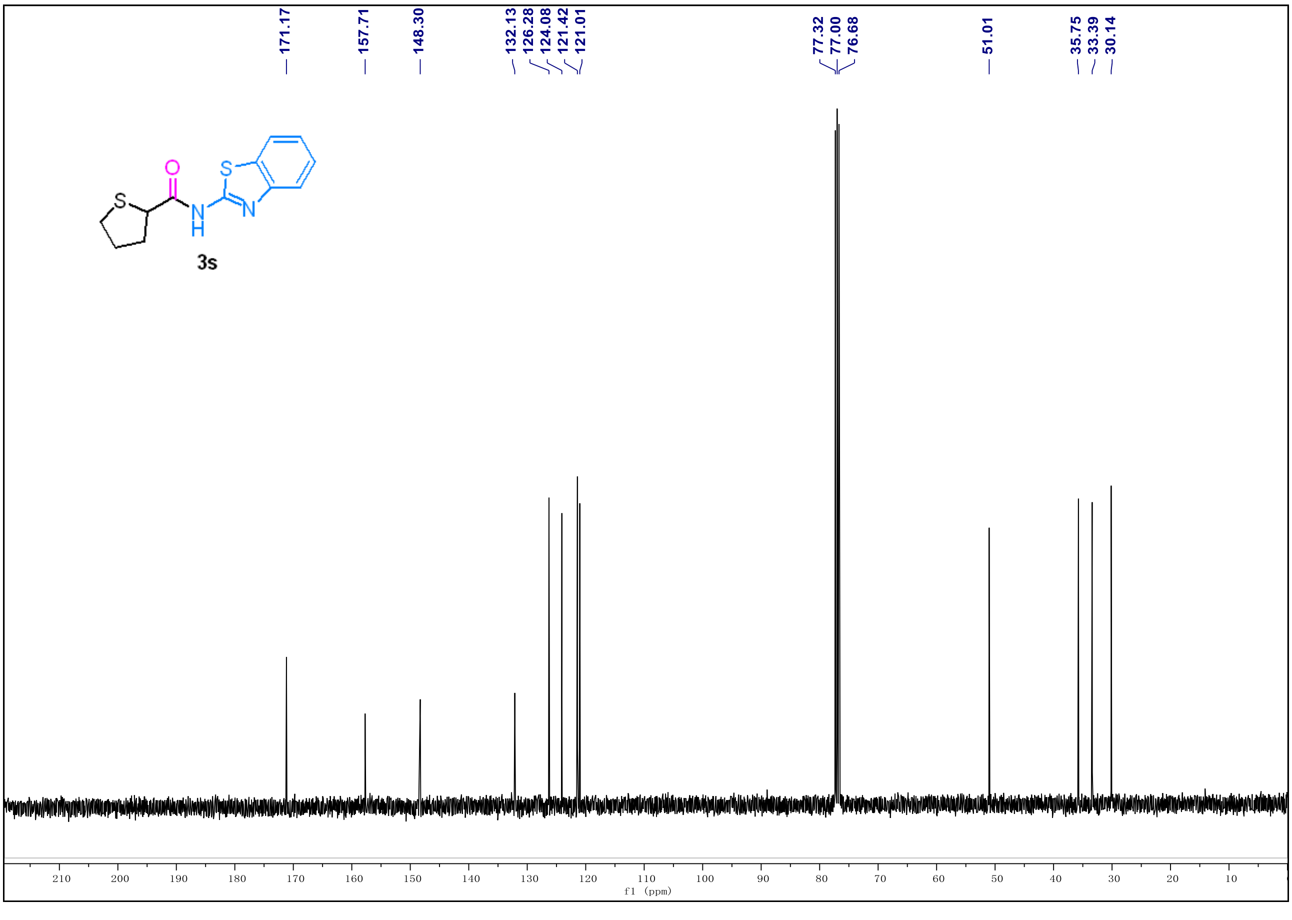

^{1}H NMR (400 MHz, CDCl3) and ^{13}C NMR (101 MHz, CDCl3) spectrum of 3t

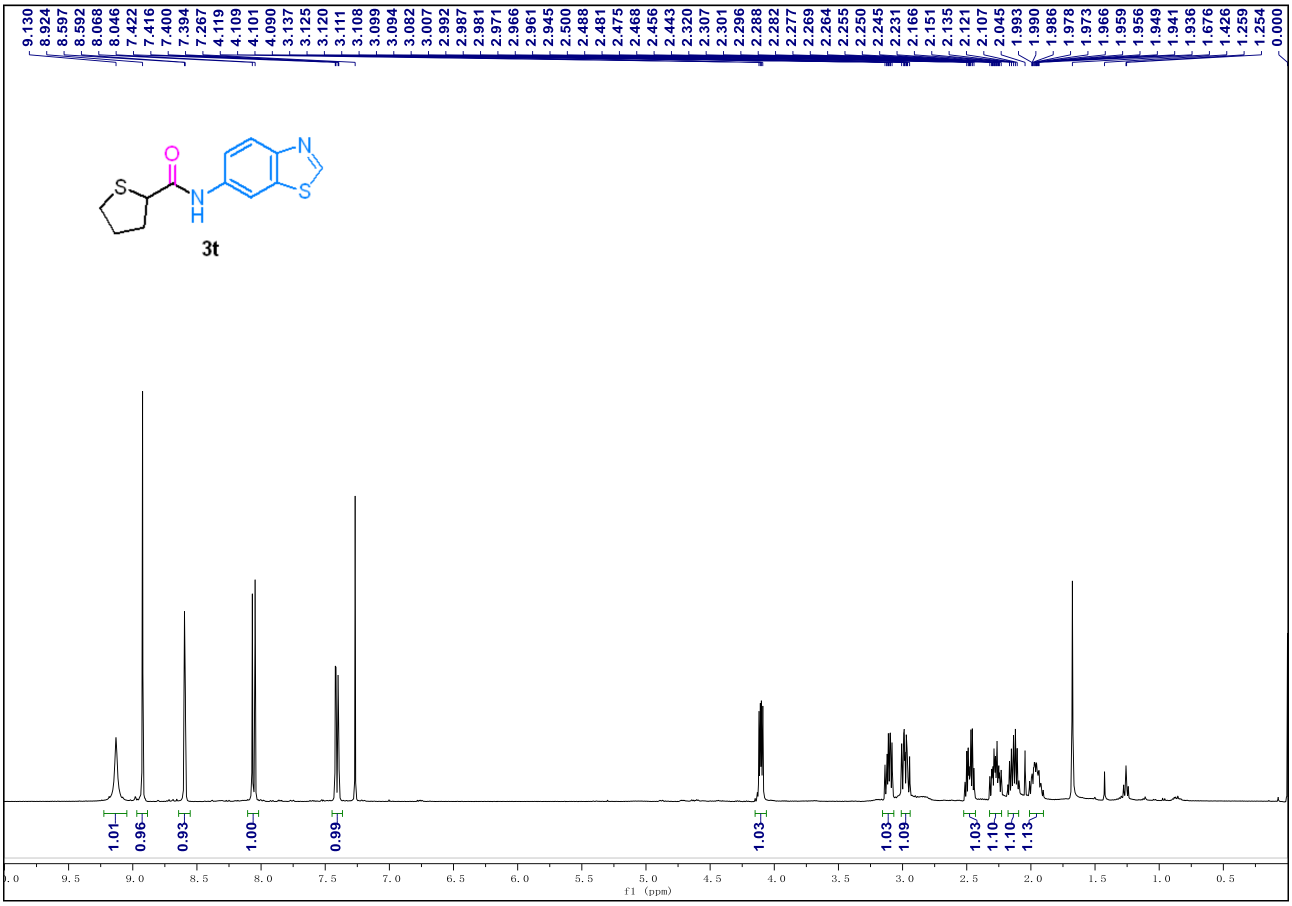

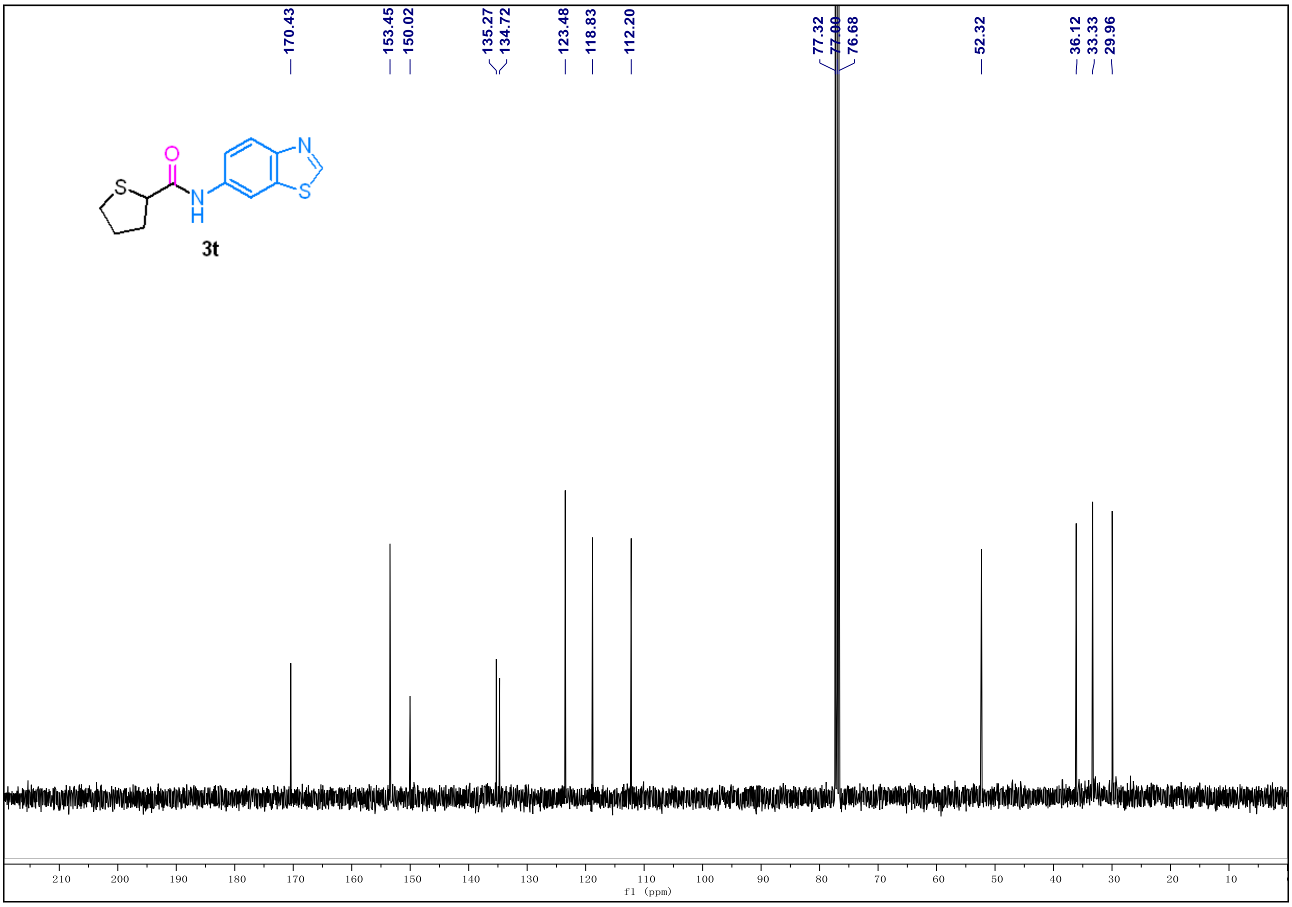

^{1}H NMR (400 MHz, $CDCl_3$) and ^{13}C NMR (101 MHz, $CDCl_3$) spectrum of 3u

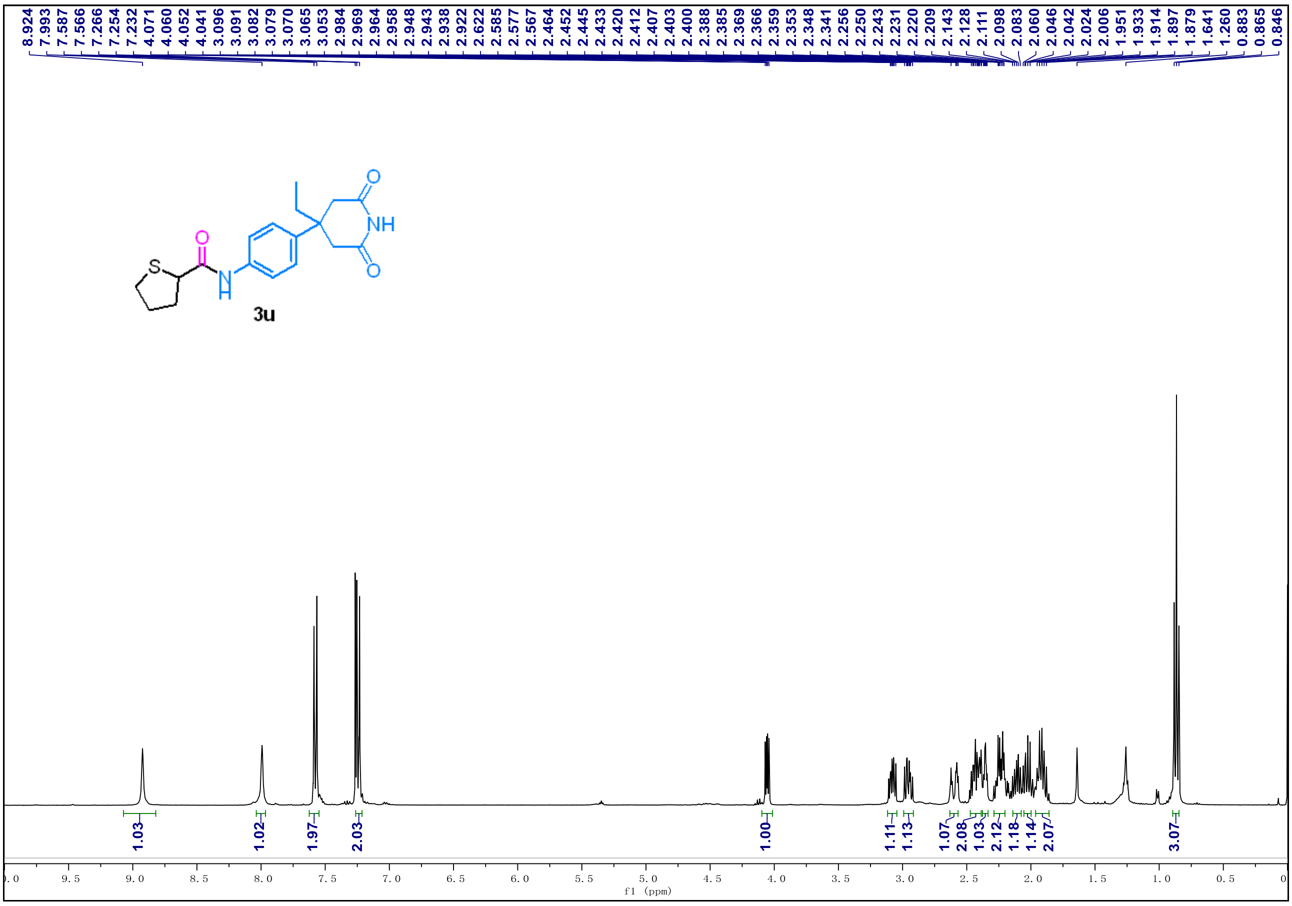

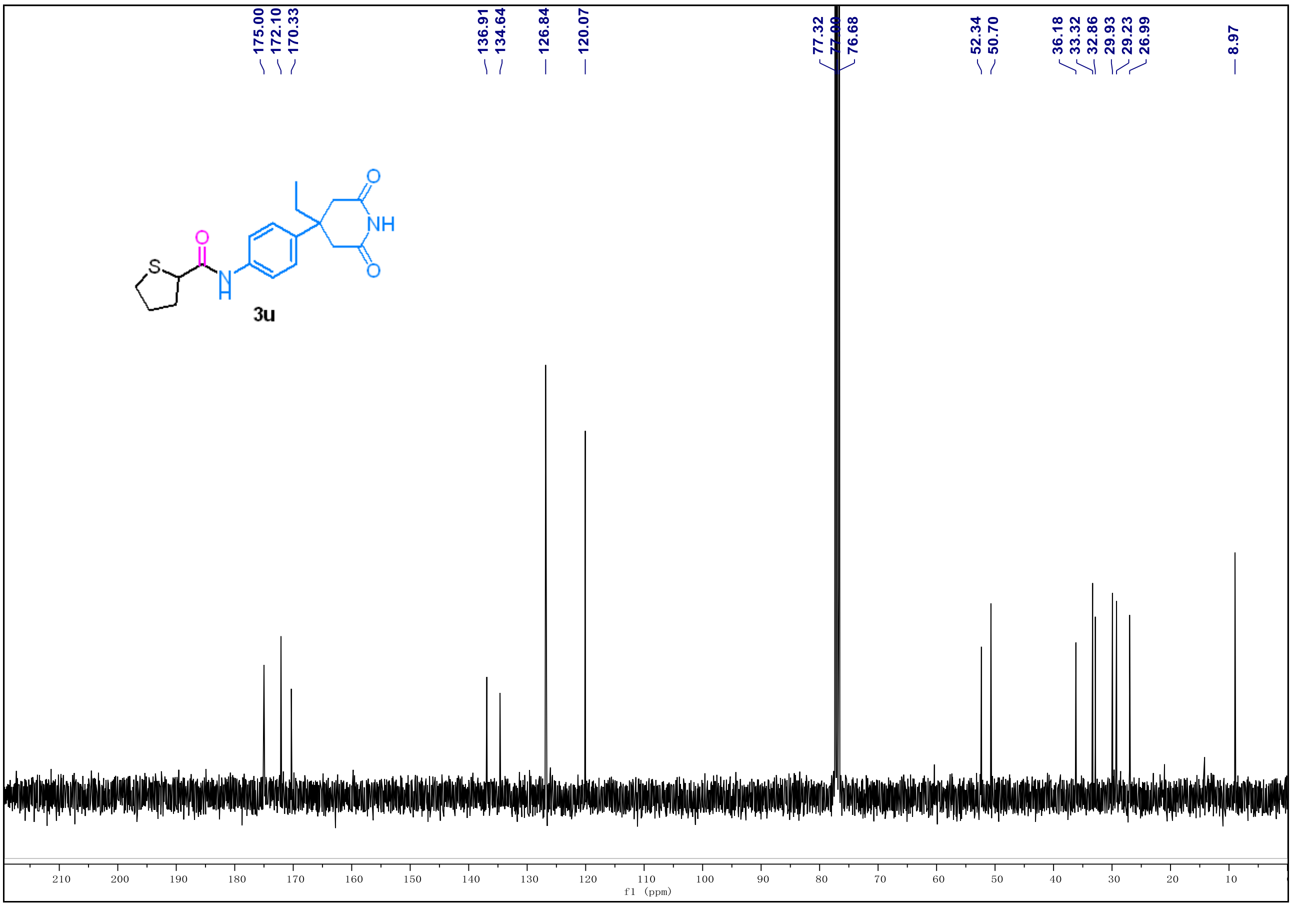

^{1}H NMR (400 MHz, $CDCl_3$) and ^{13}C NMR (101 MHz, $CDCl_3$) spectrum of 3v

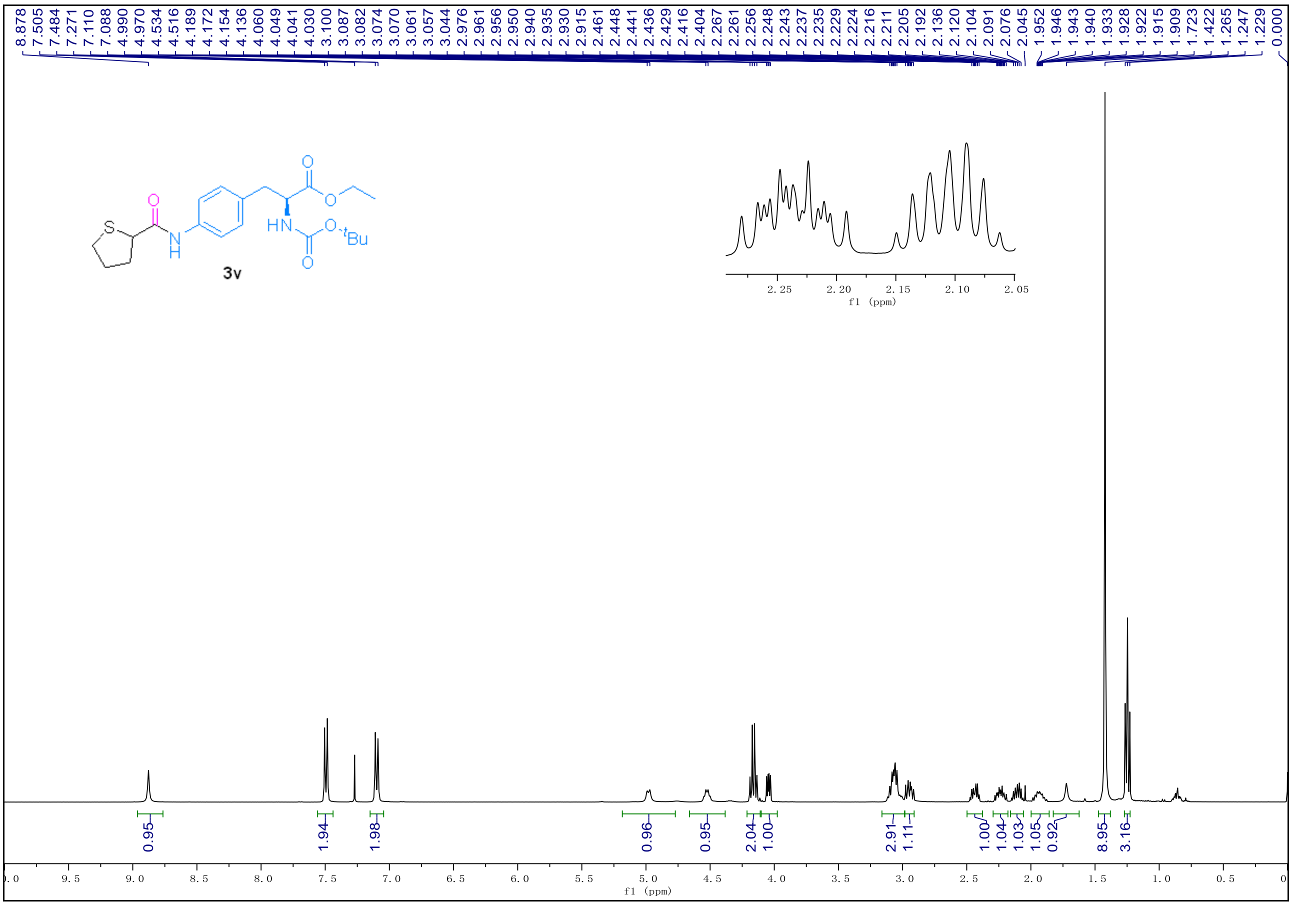

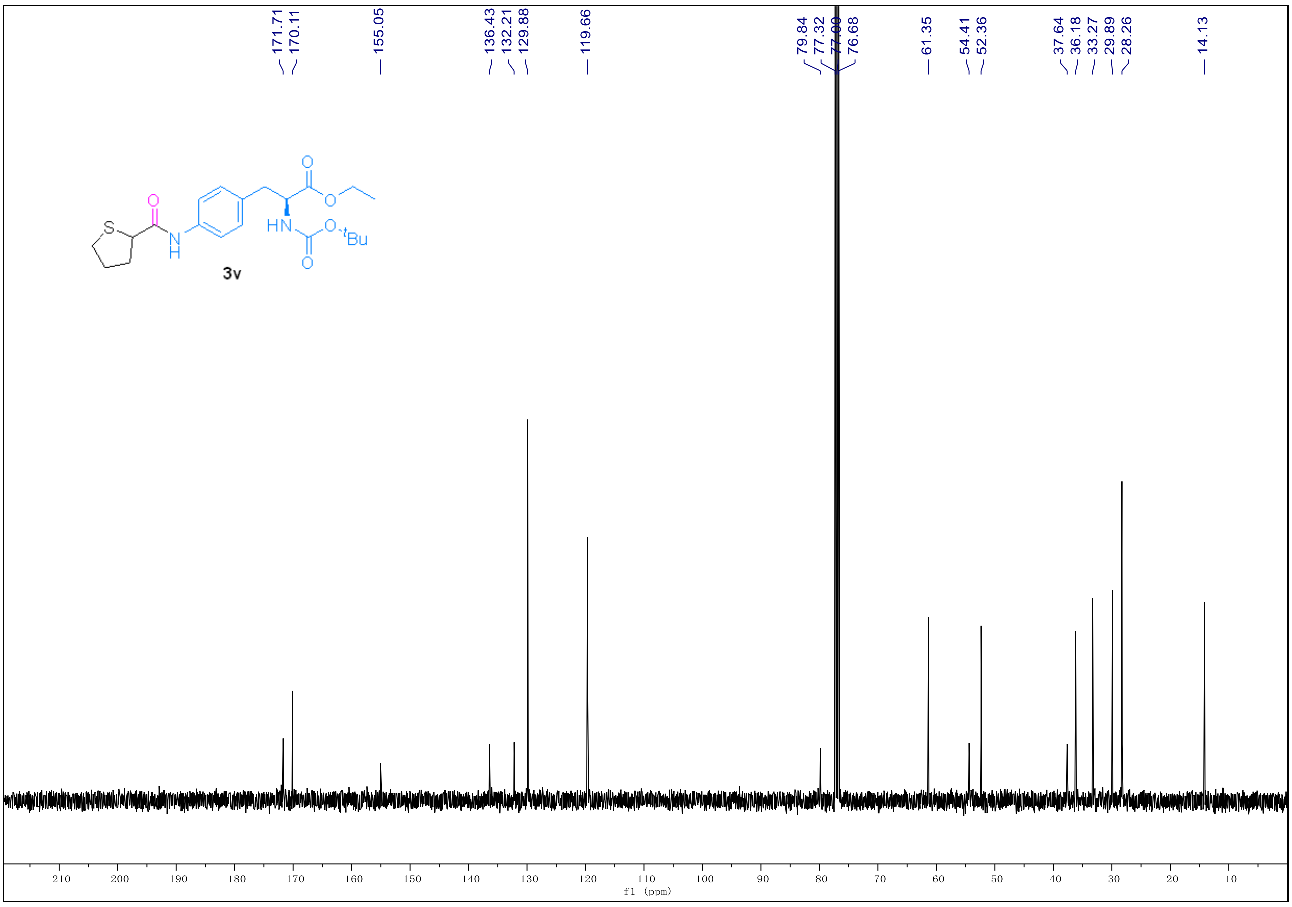

^{1}H NMR (400 MHz, $CDCl_3$) and ^{13}C NMR (101 MHz, $CDCl_3$) spectrum of 3w

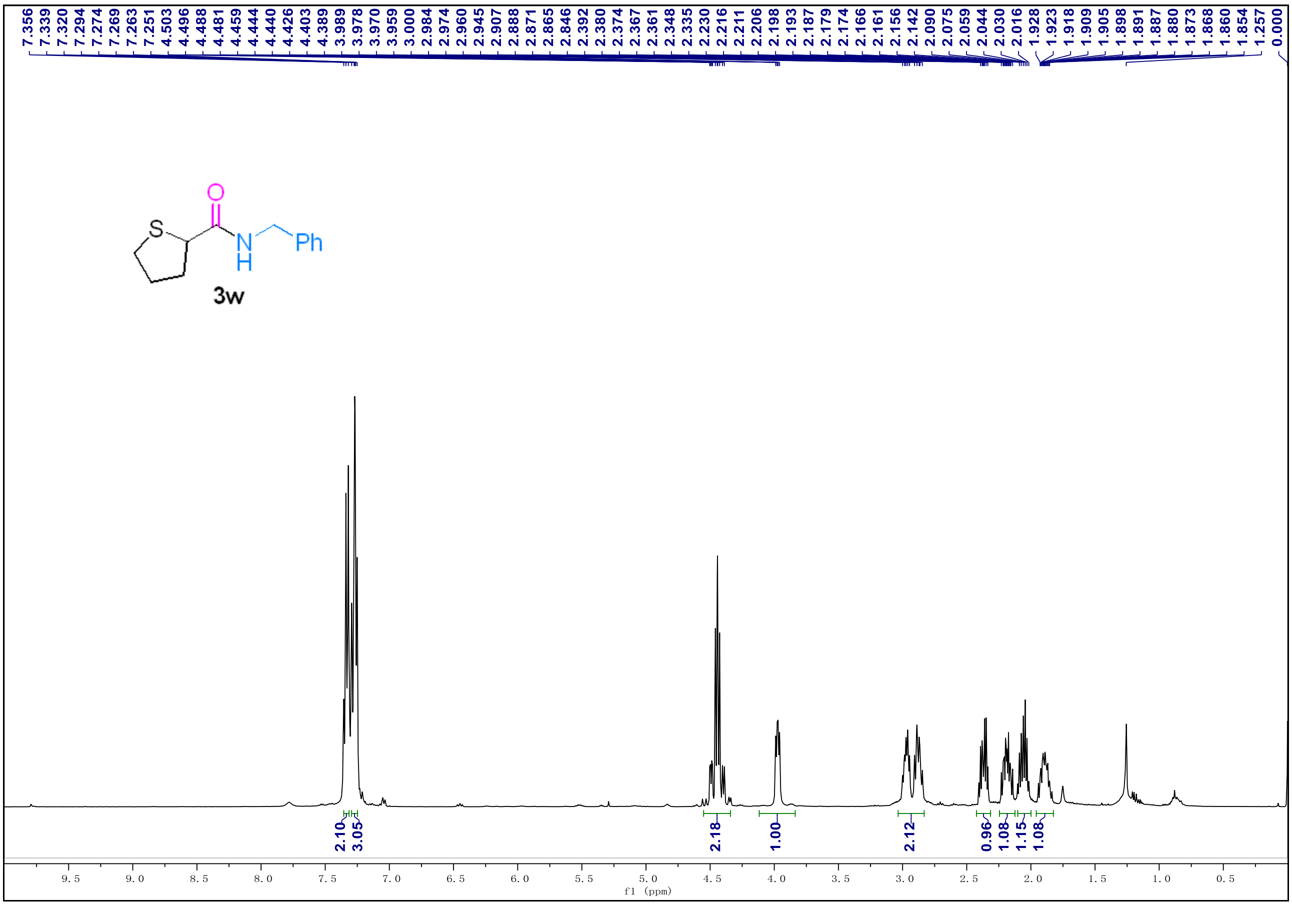

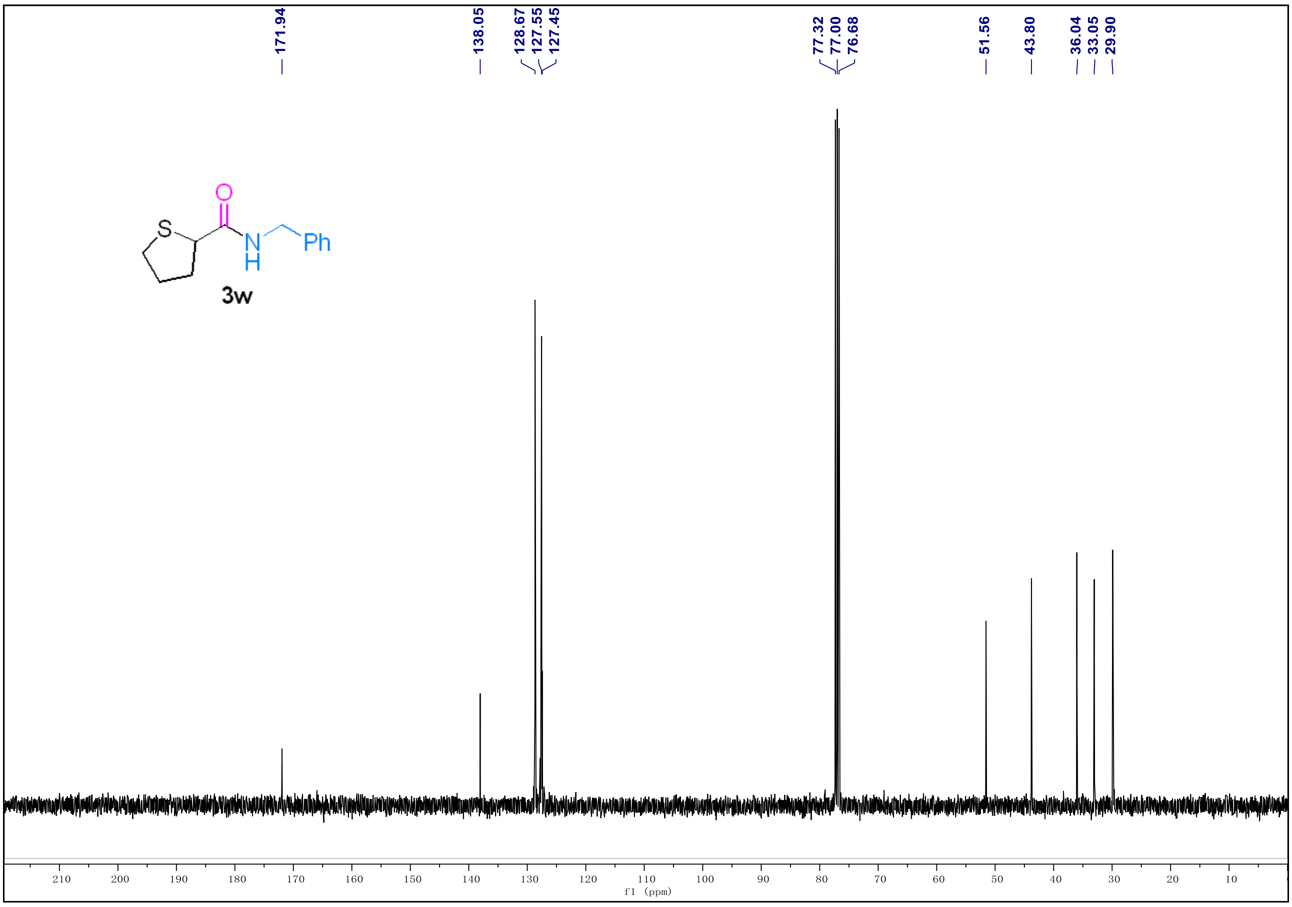

^{1}H NMR (400 MHz, $CDCl_3$) and ^{13}C NMR (101 MHz, $CDCl_3$) spectrum of 3x

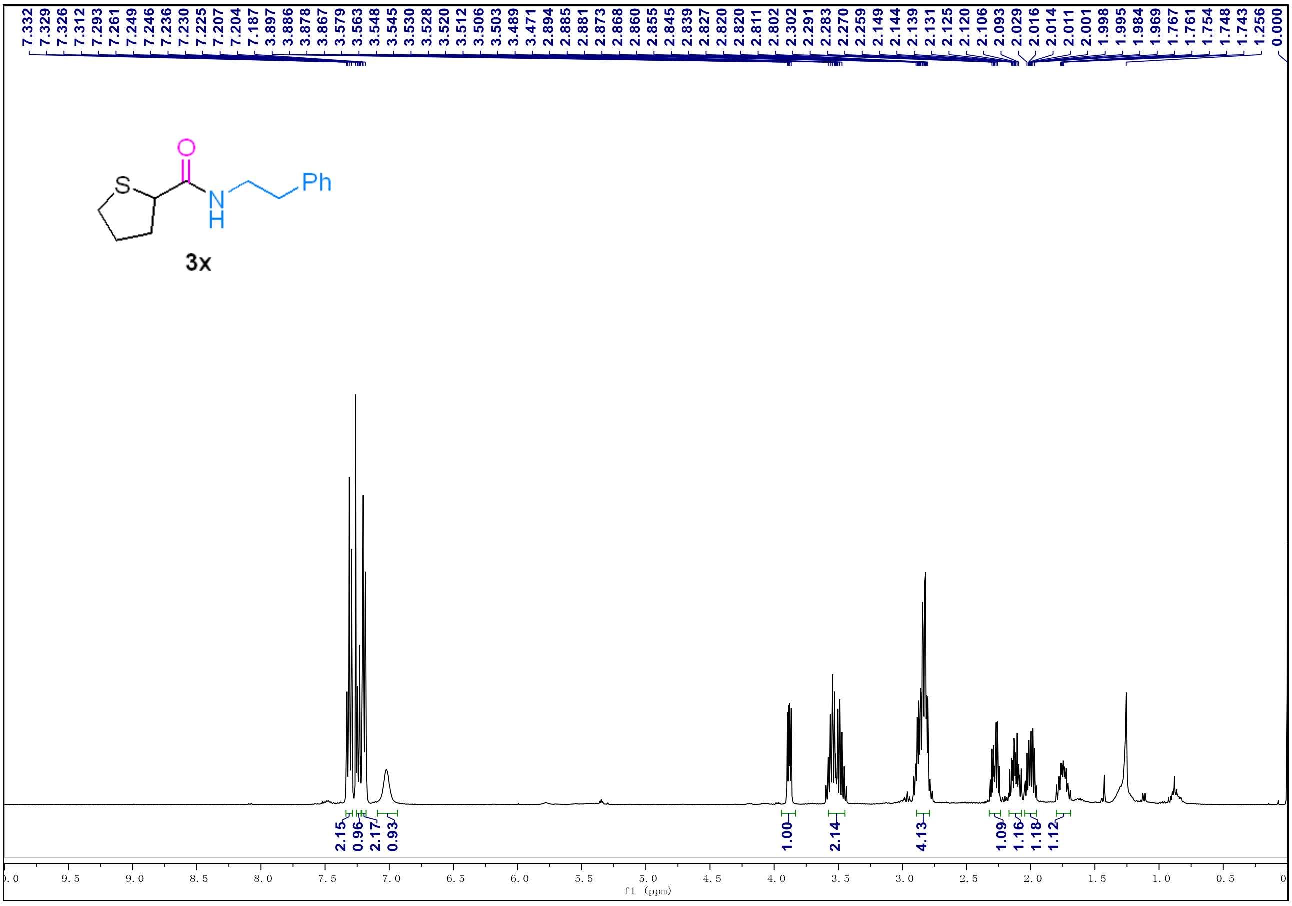

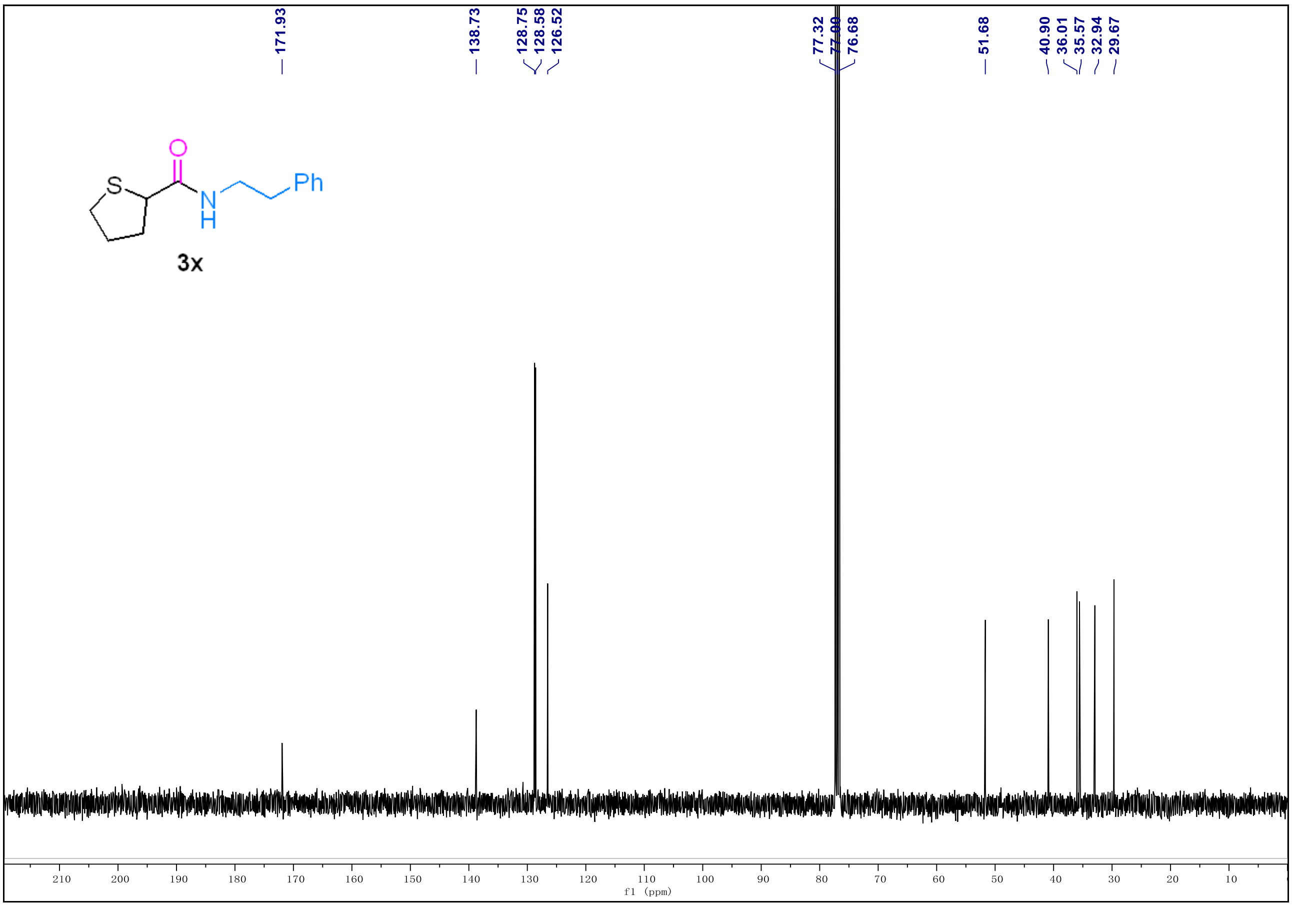

^{1}H NMR (400 MHz, $CDCl_3$) and ^{13}C NMR (101 MHz, $CDCl_3$) spectrum of 3y

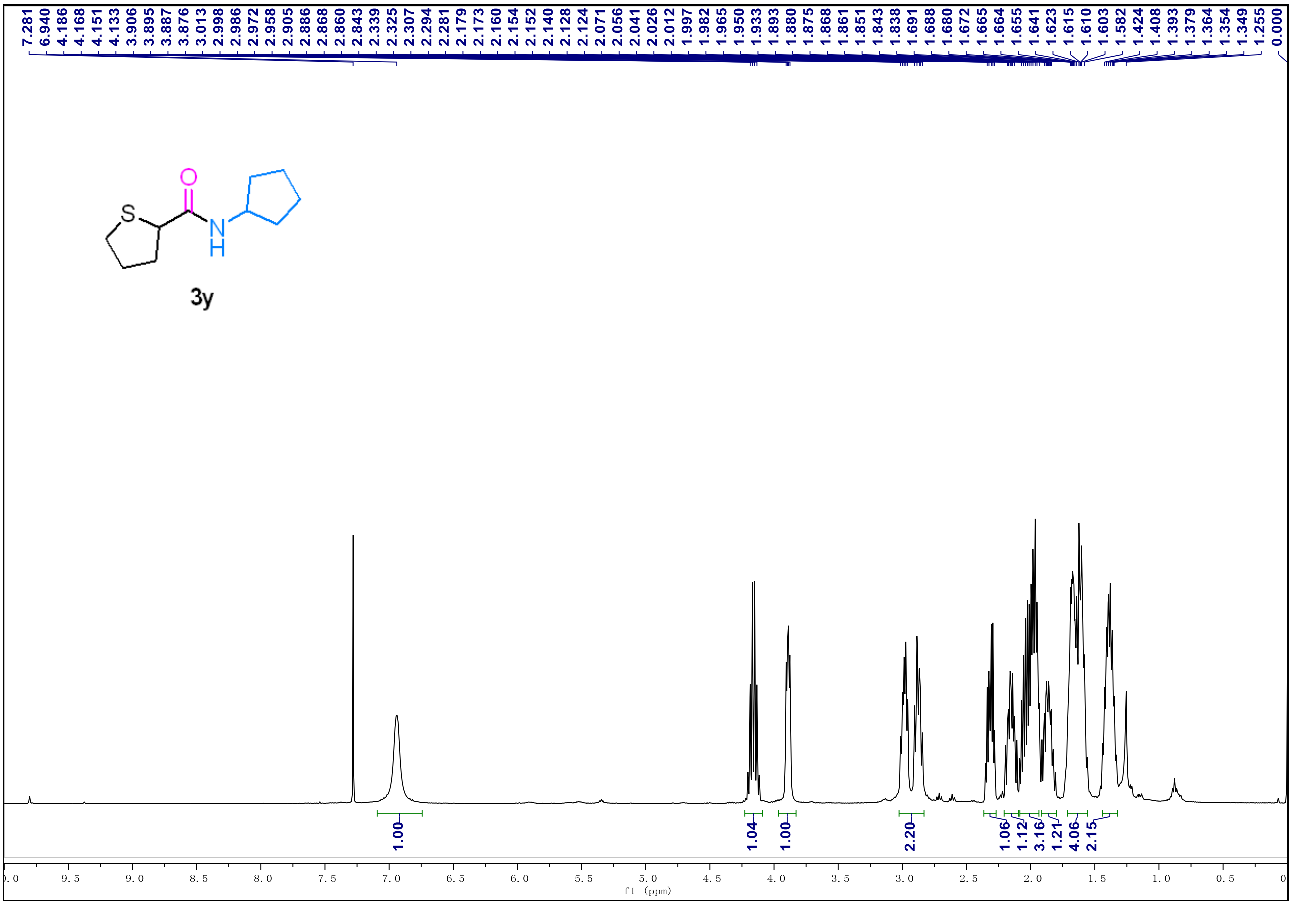

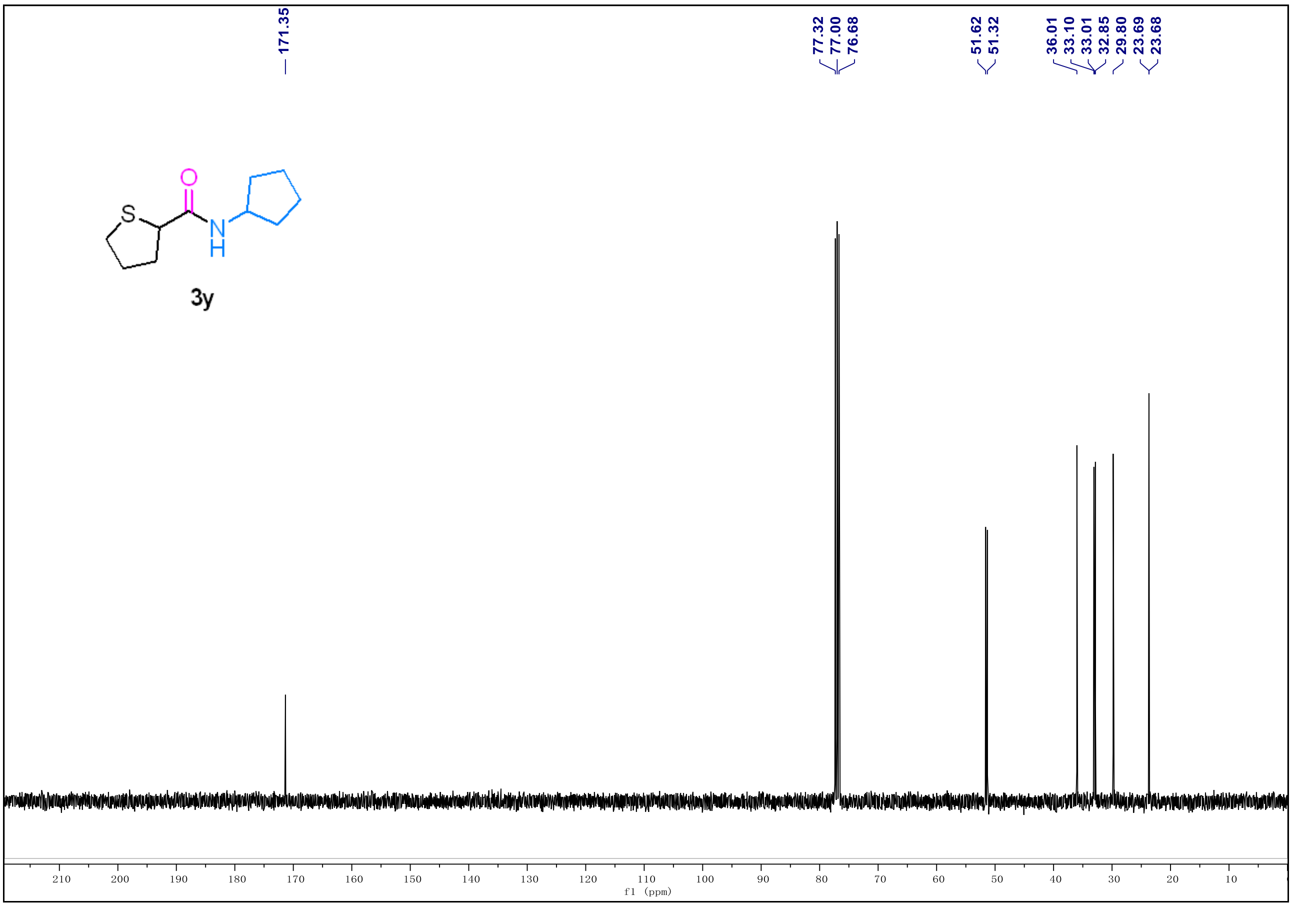

^{1}H NMR (400 MHz, $CDCl_3$) and ^{13}C NMR (101 MHz, $CDCl_3$) spectrum of 3z

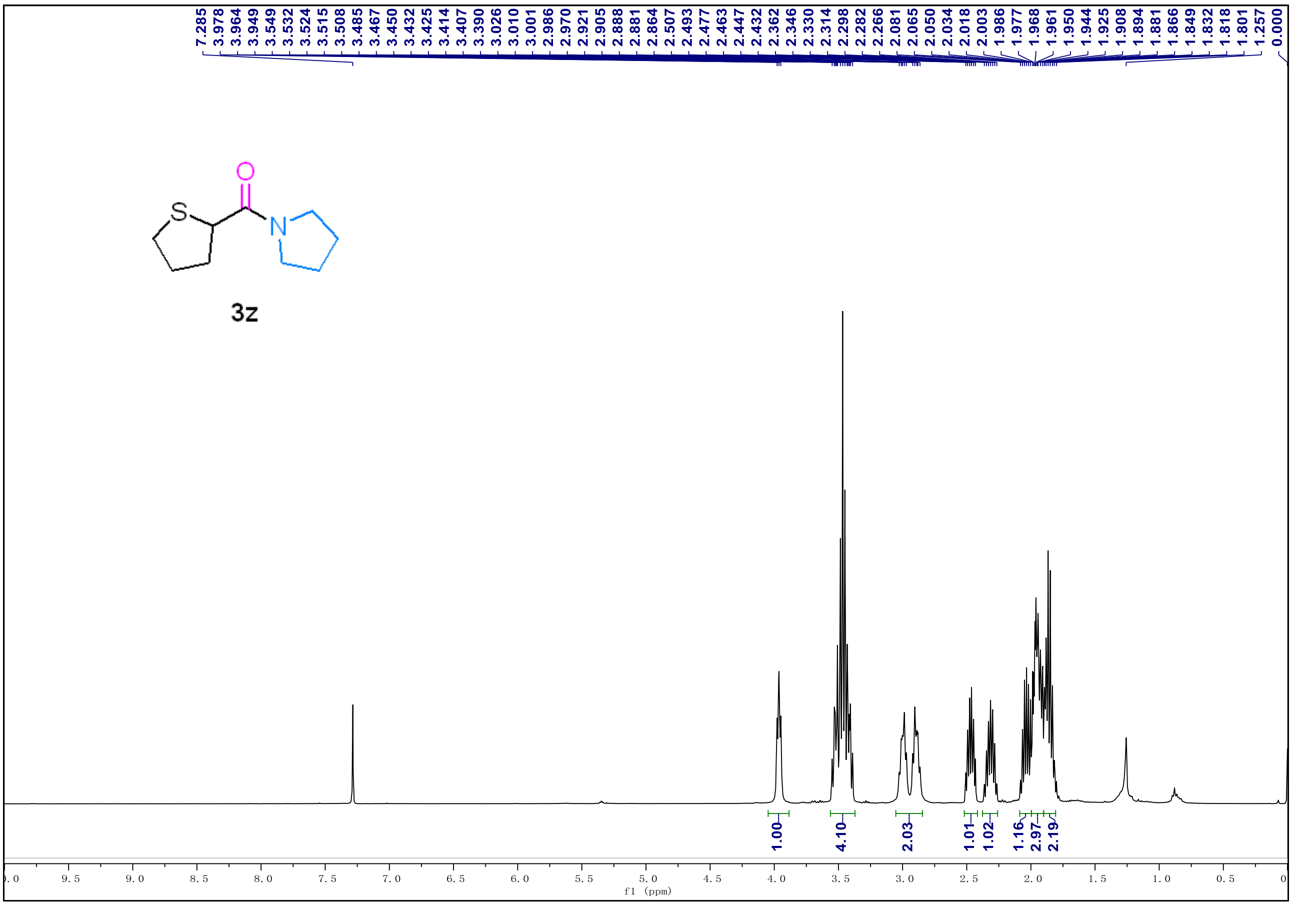

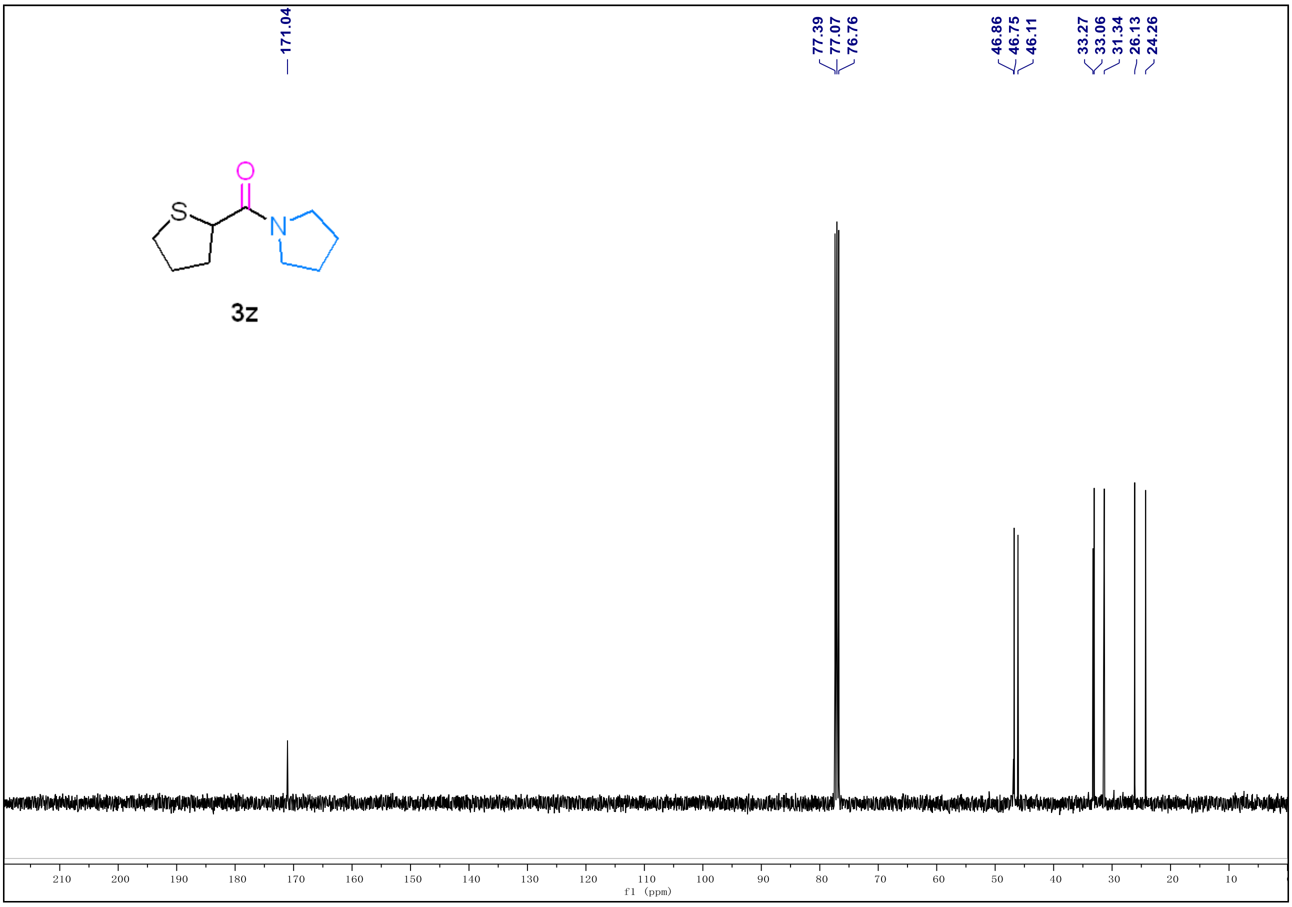

^{1}H NMR (400 MHz, $CDCl_3$) and ^{13}C NMR (101 MHz, $CDCl_3$) spectrum of 3aa

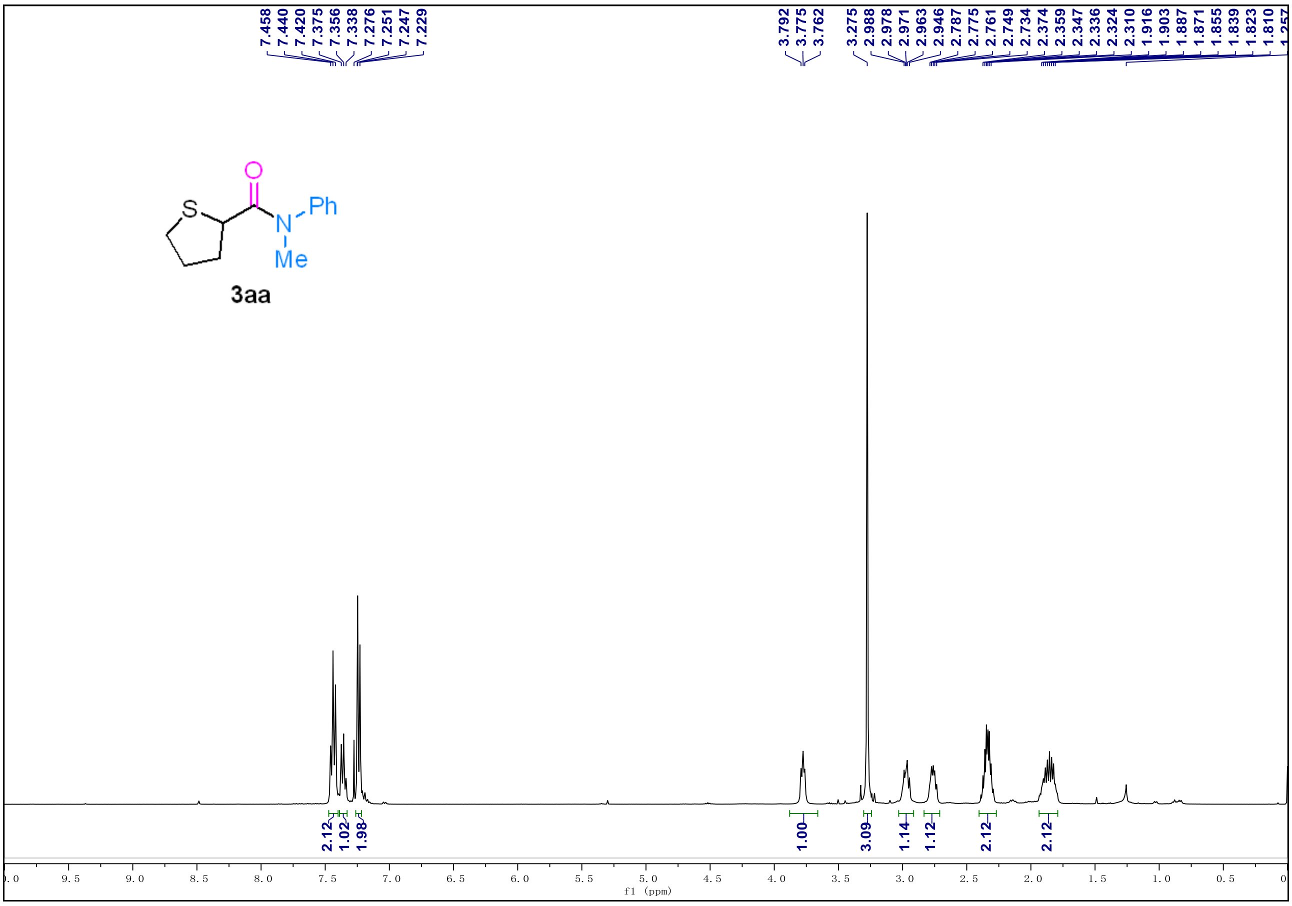

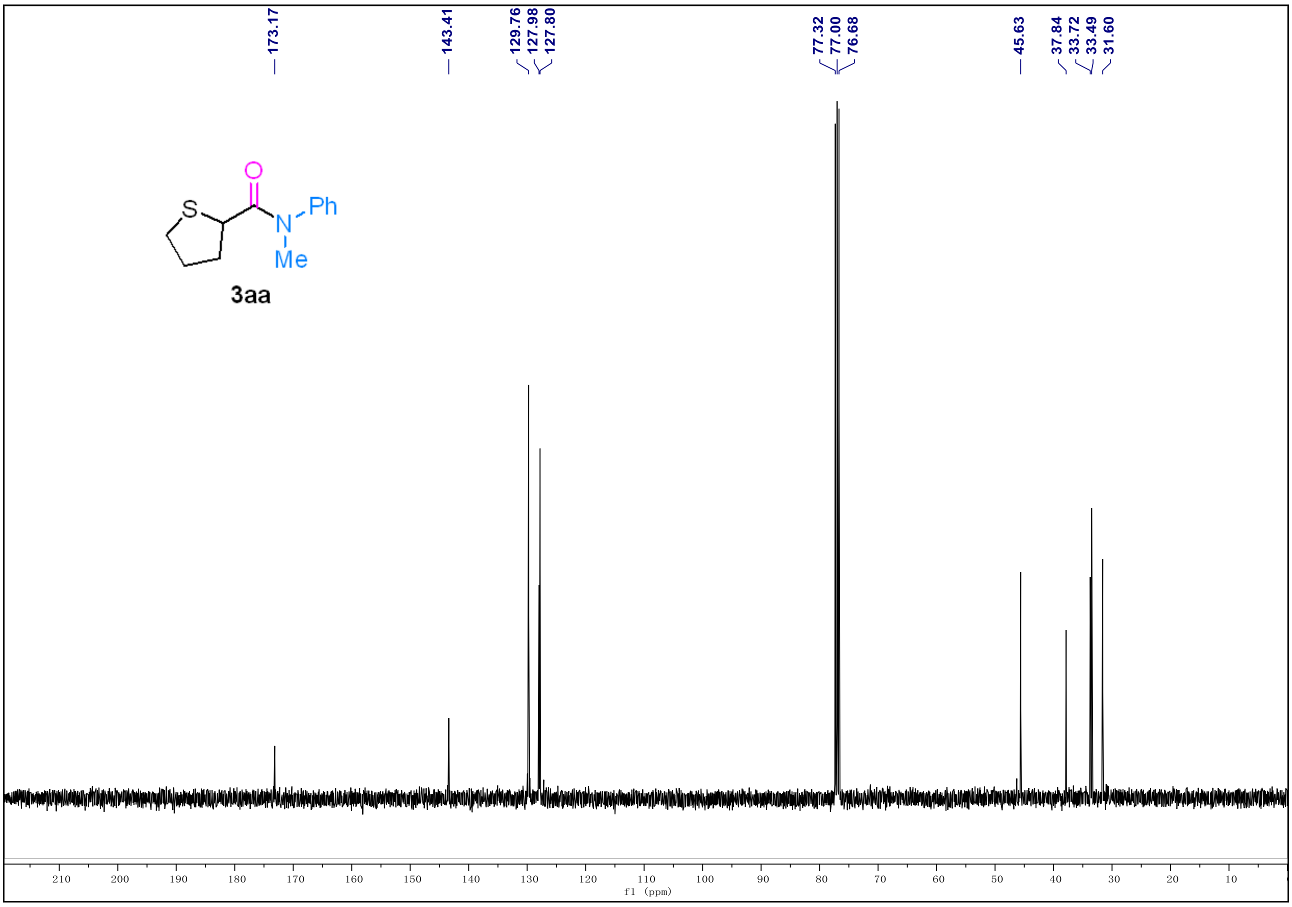

^{1}H NMR (400 MHz, $CDCl_3$) and ^{13}C NMR (101 MHz, $CDCl_3$) spectrum of 5a

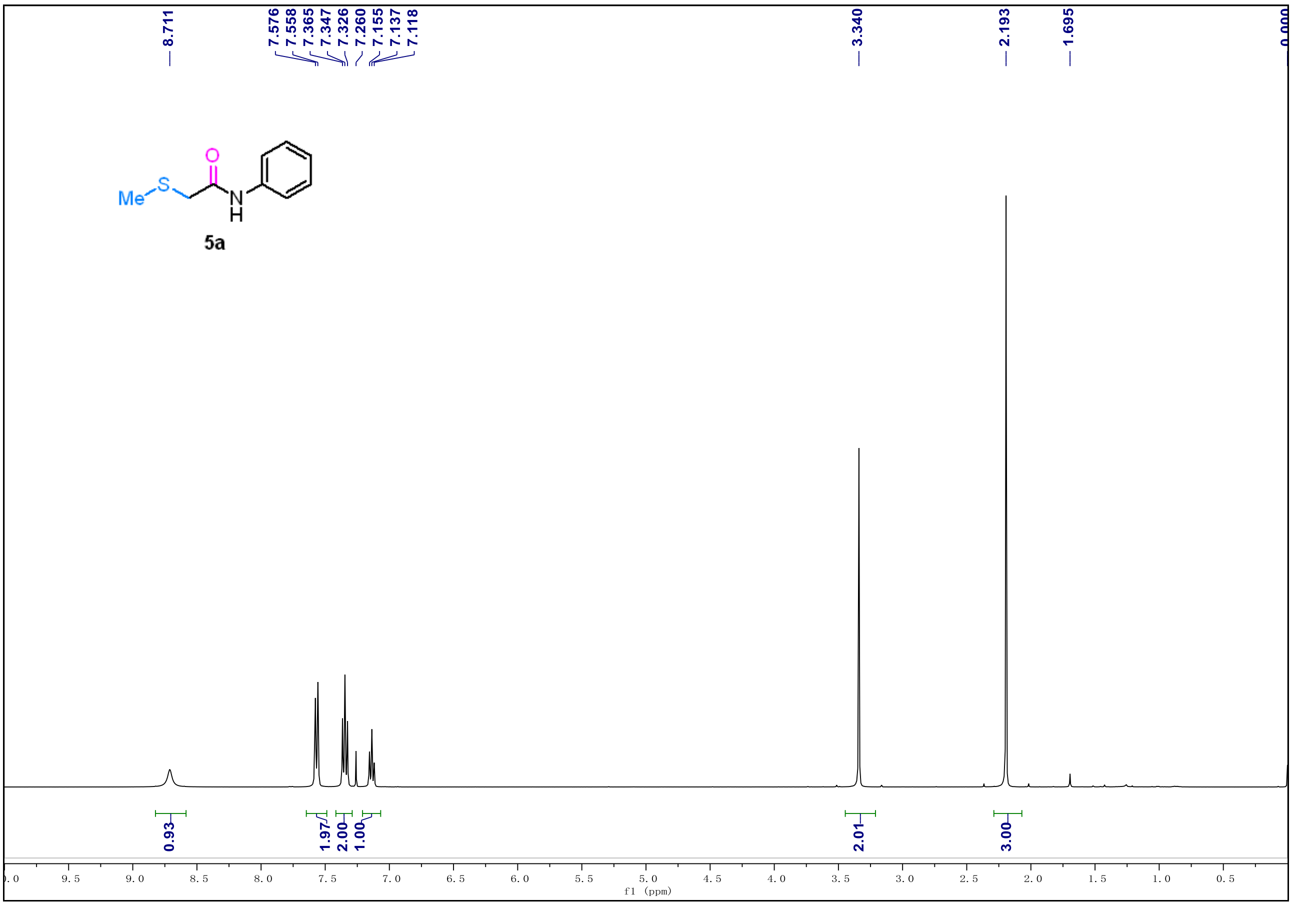

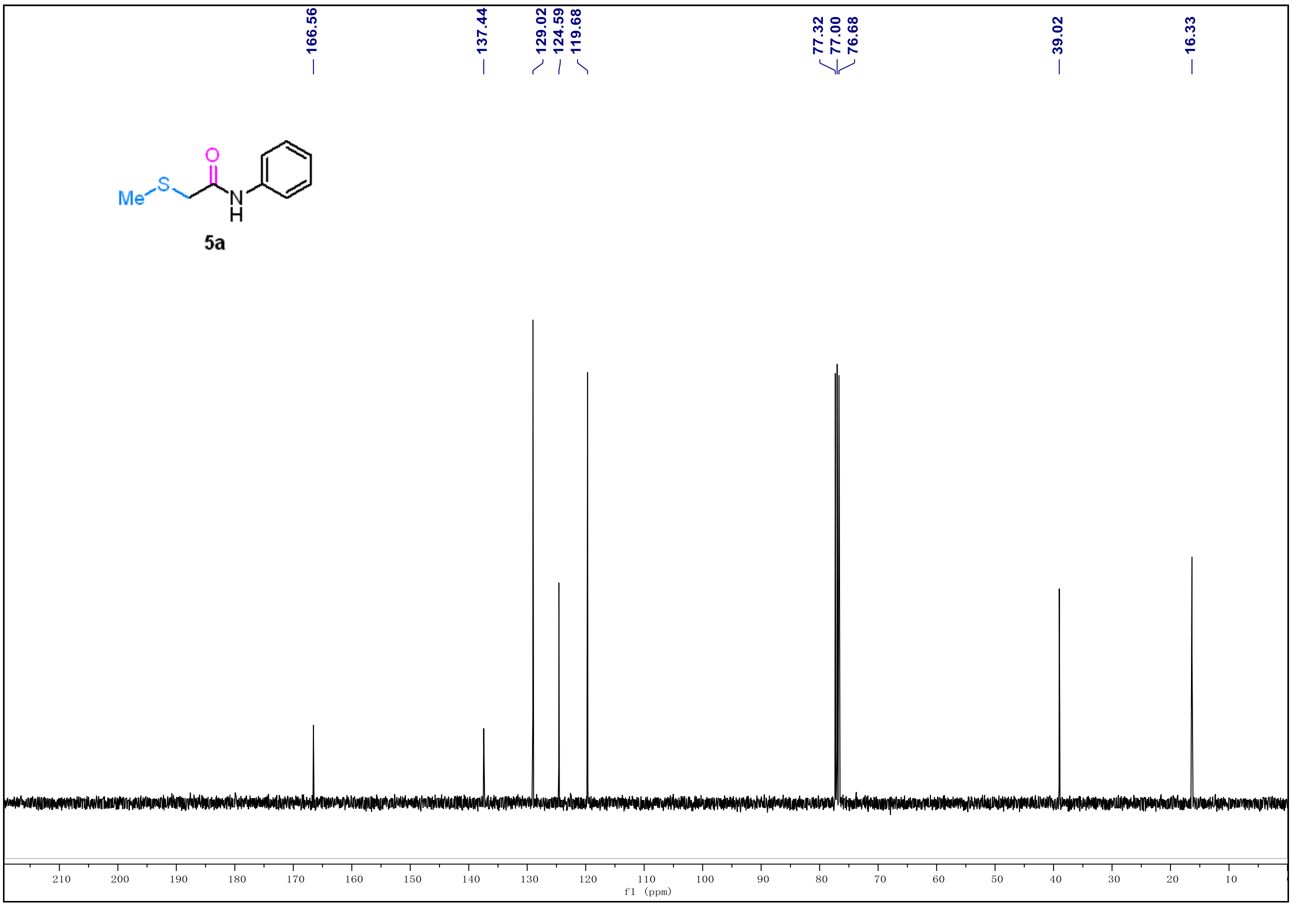

^{1}H NMR (400 MHz, CDCl3) and ^{13}C NMR (101 MHz, CDCl3) spectrum of 5b

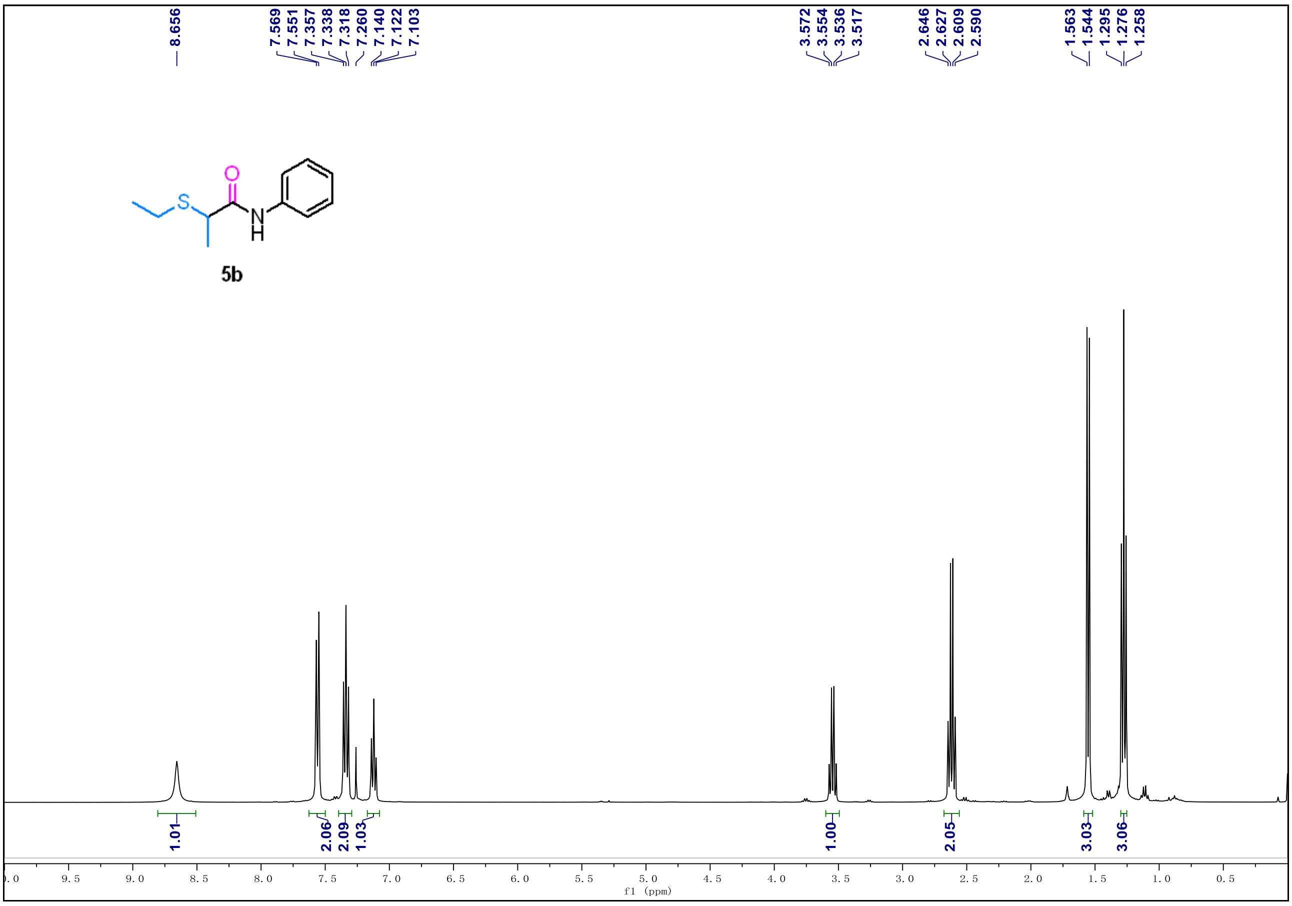

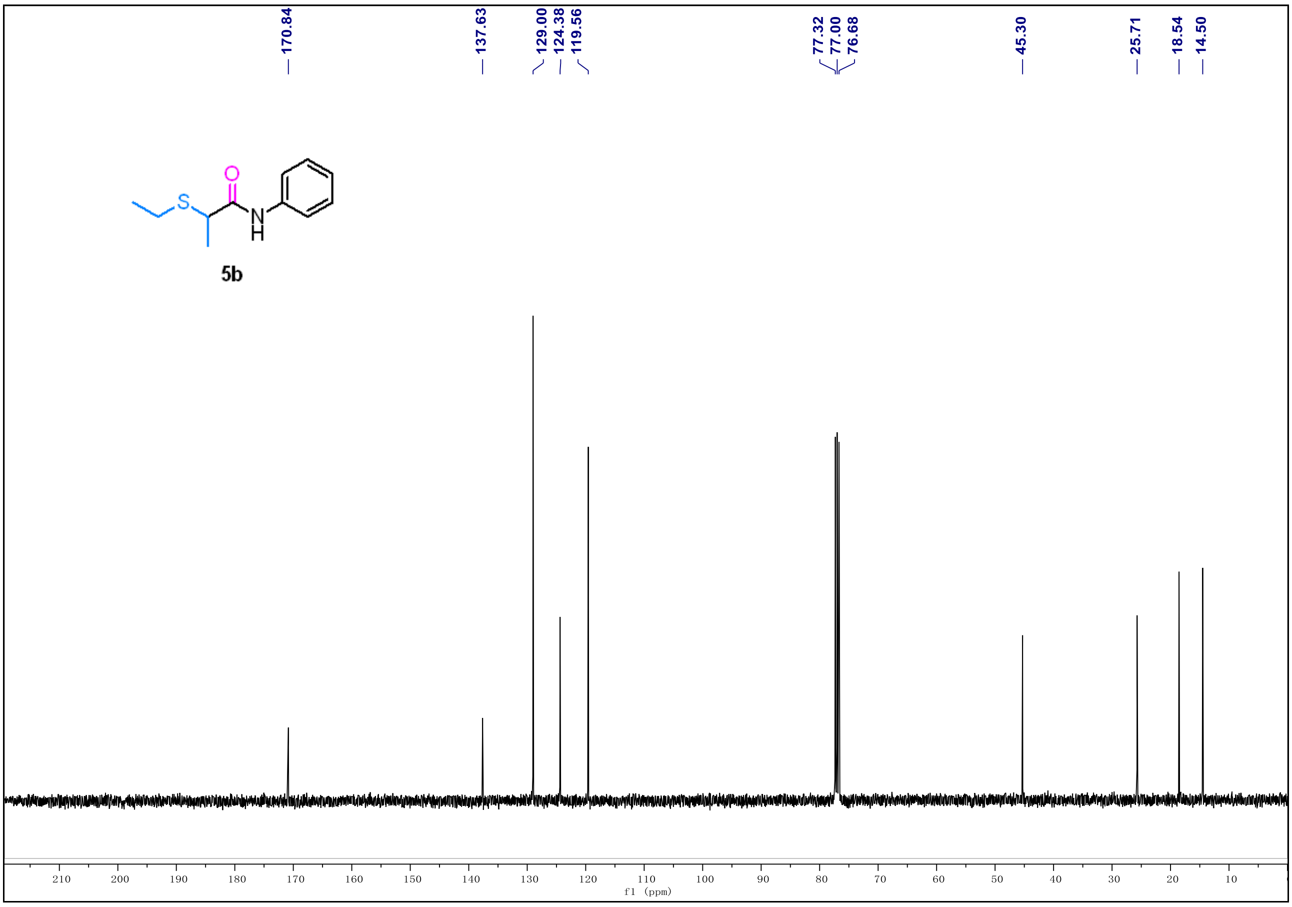

^{1}H NMR (400 MHz, CDCl3) and ^{13}C NMR (101 MHz, CDCl3) spectrum of 5c

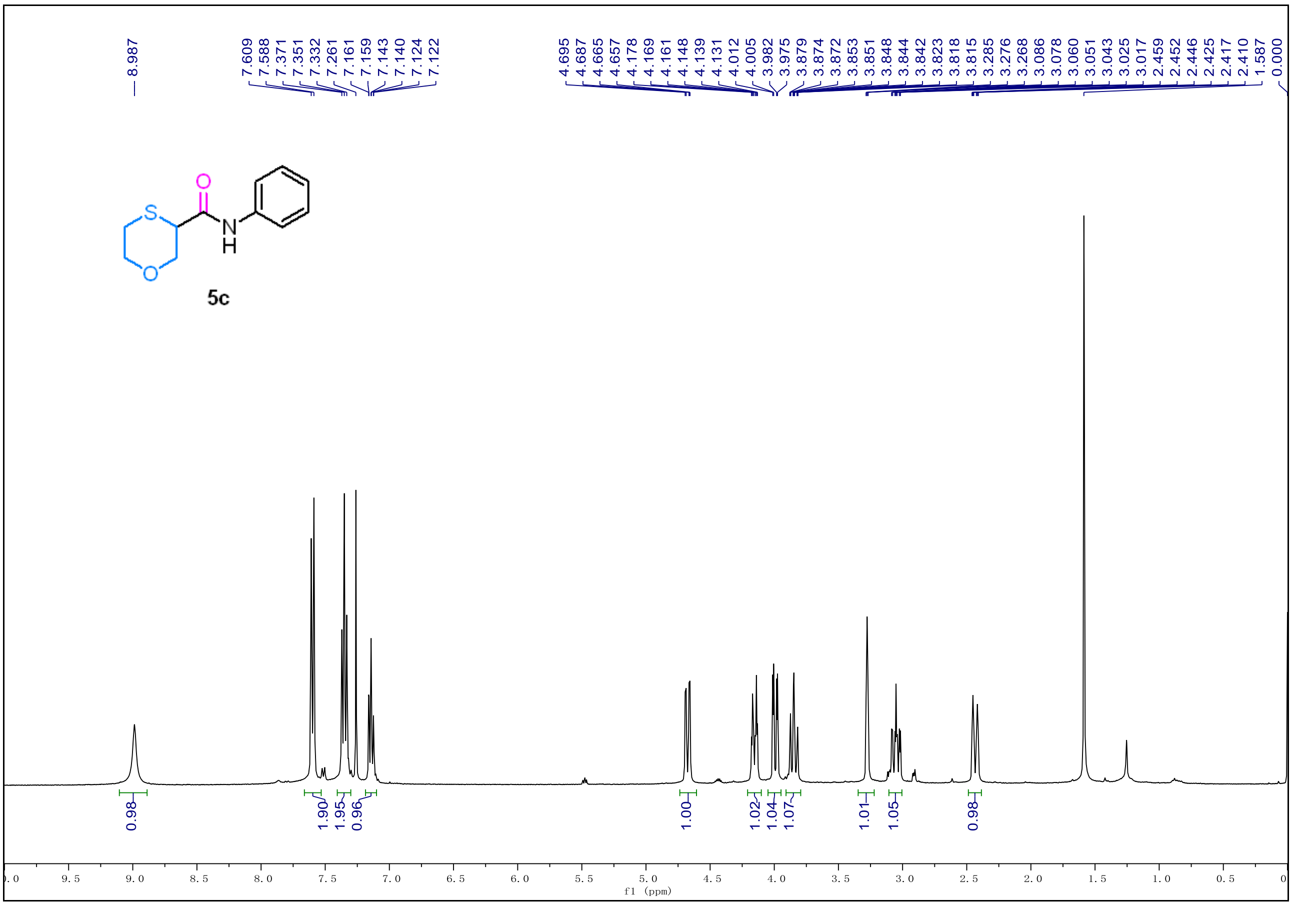

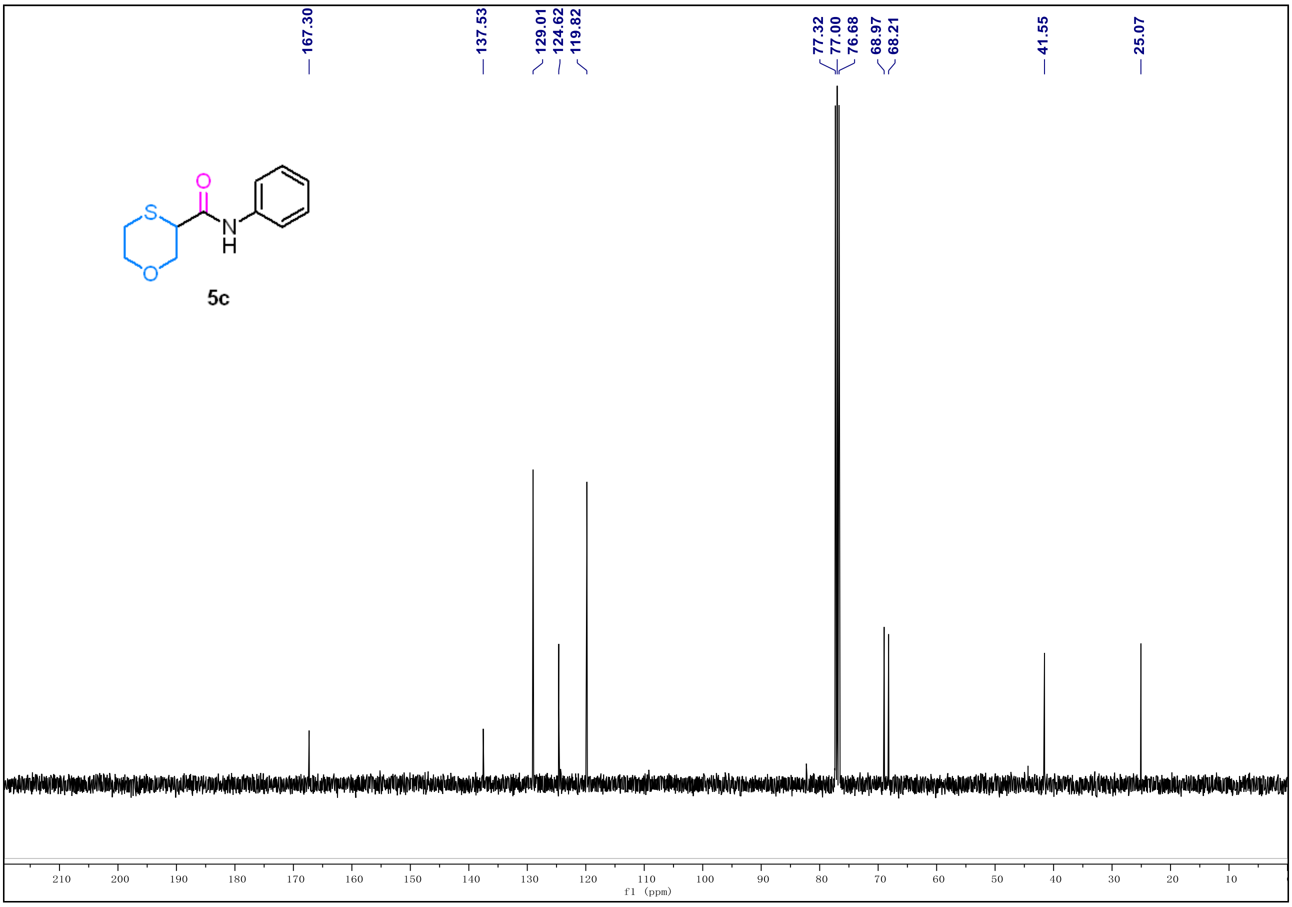

^{1}H NMR (400 MHz, CDCl$_3$) and ^{13}C NMR (101 MHz, CDCl$_3$) spectrum of 5d

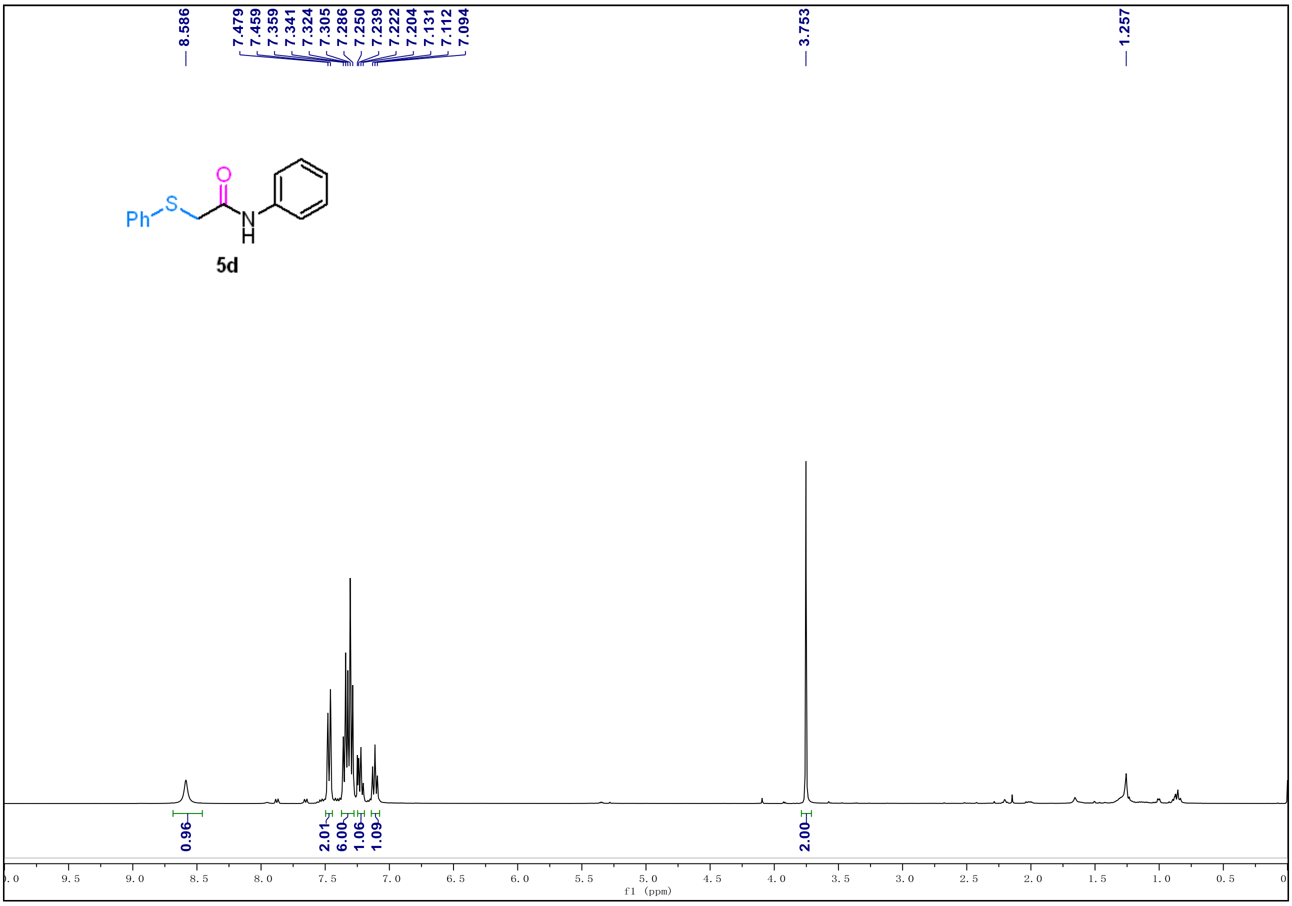

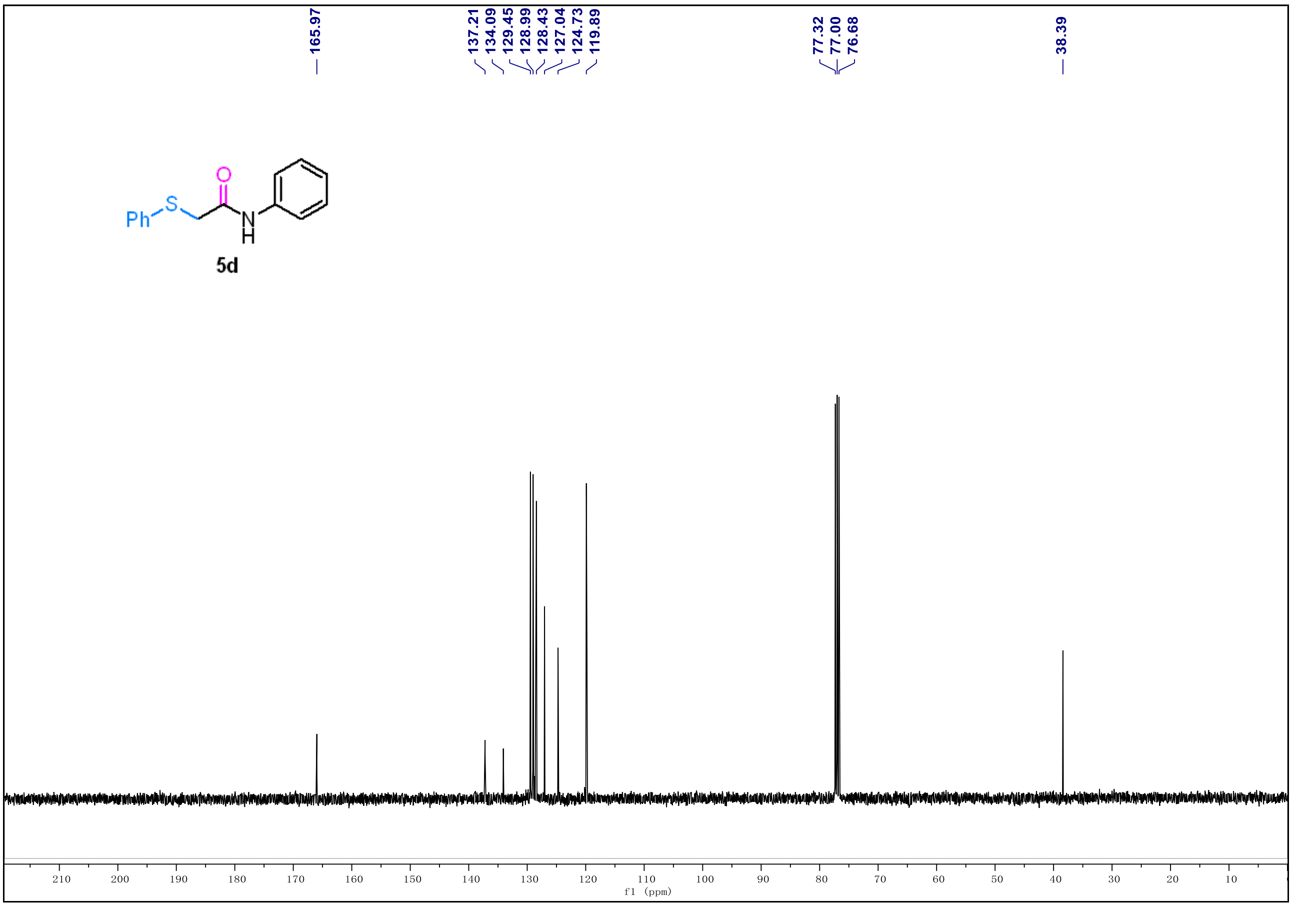

^{1}H NMR (400 MHz, $CDCl_3$) and ^{13}C NMR (101 MHz, $CDCl_3$) spectrum of 5e

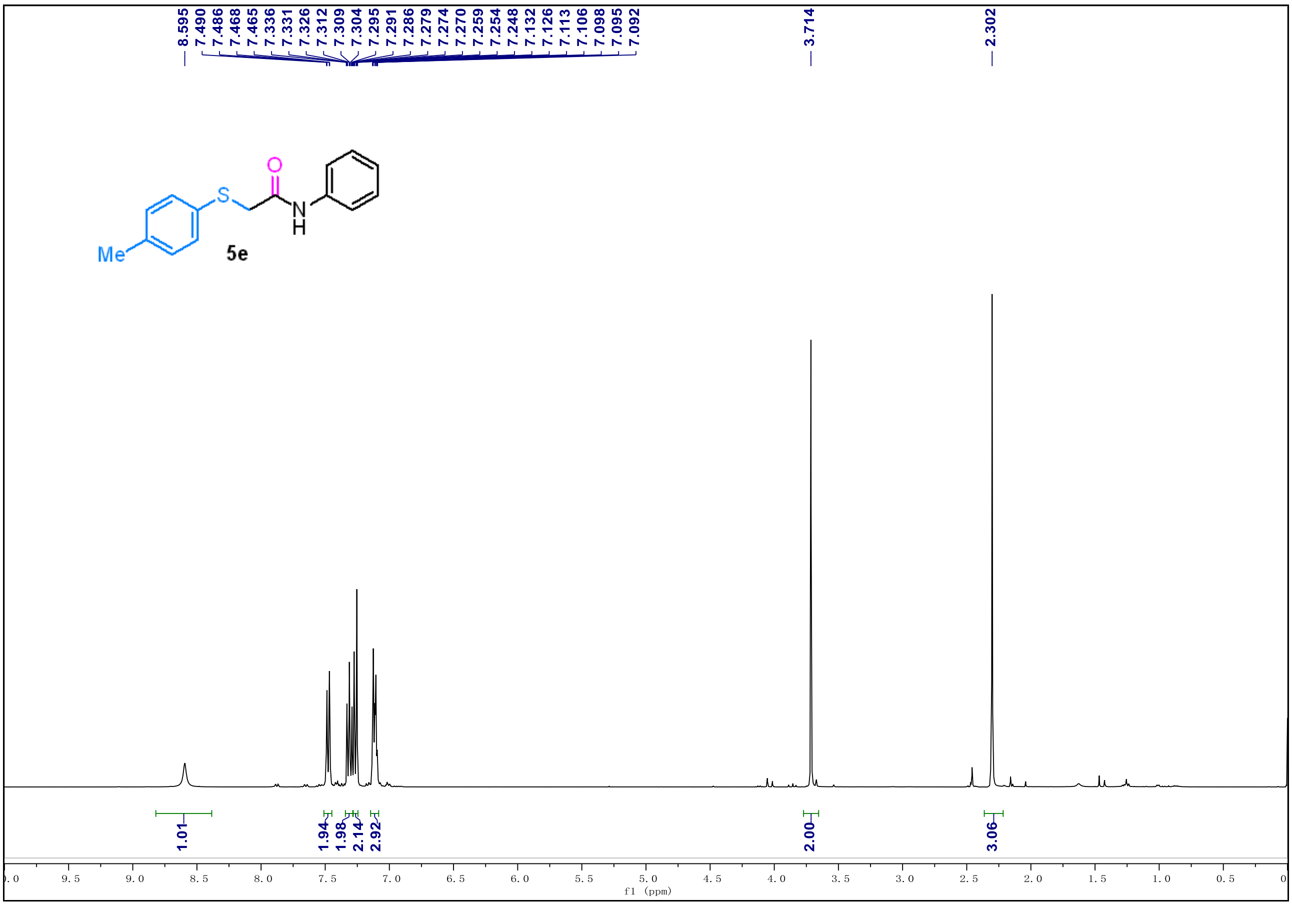

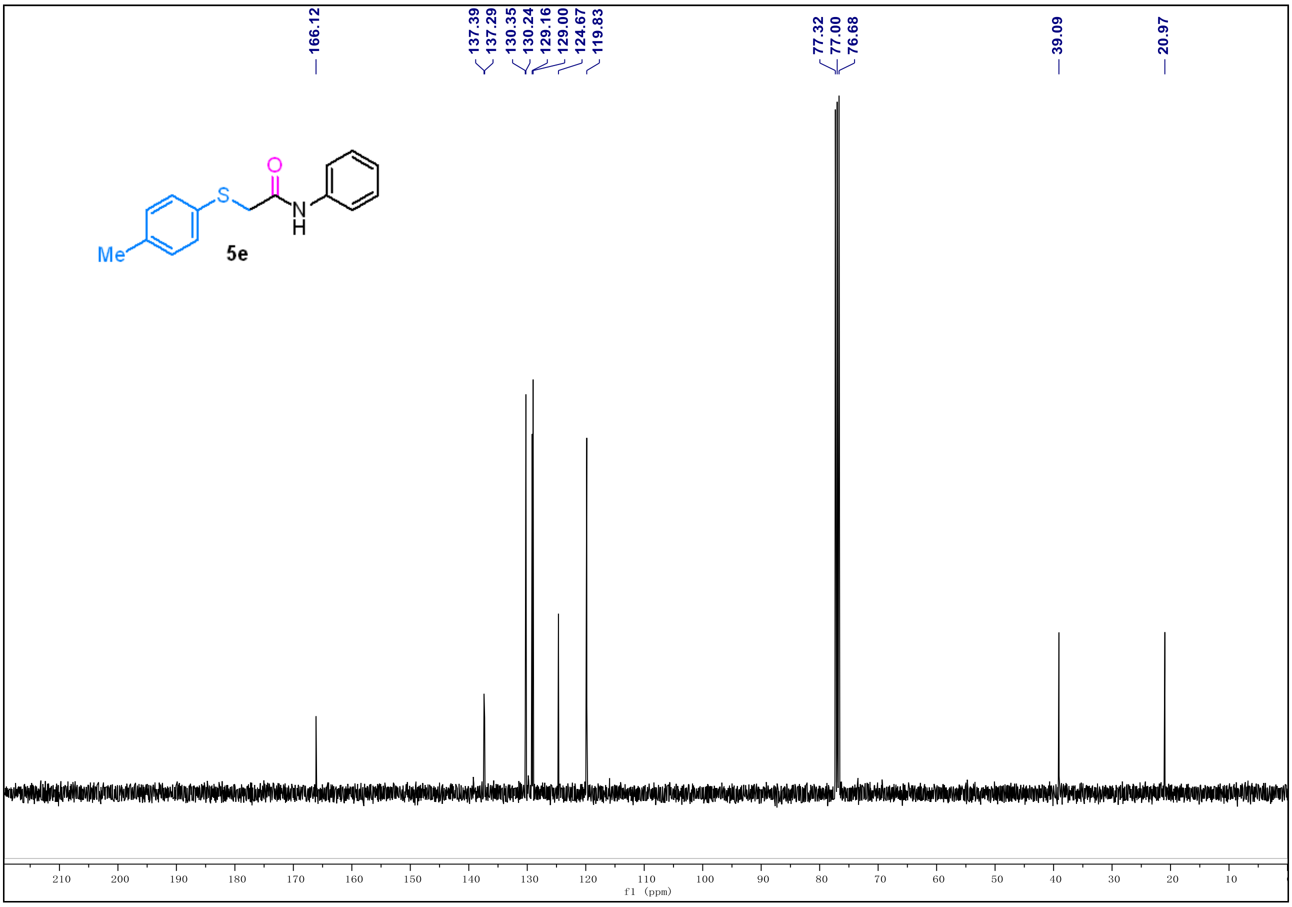

^{1}H NMR (400 MHz, CDCl$_3$) and ^{13}C NMR (101 MHz, CDCl$_3$) spectrum of 5f

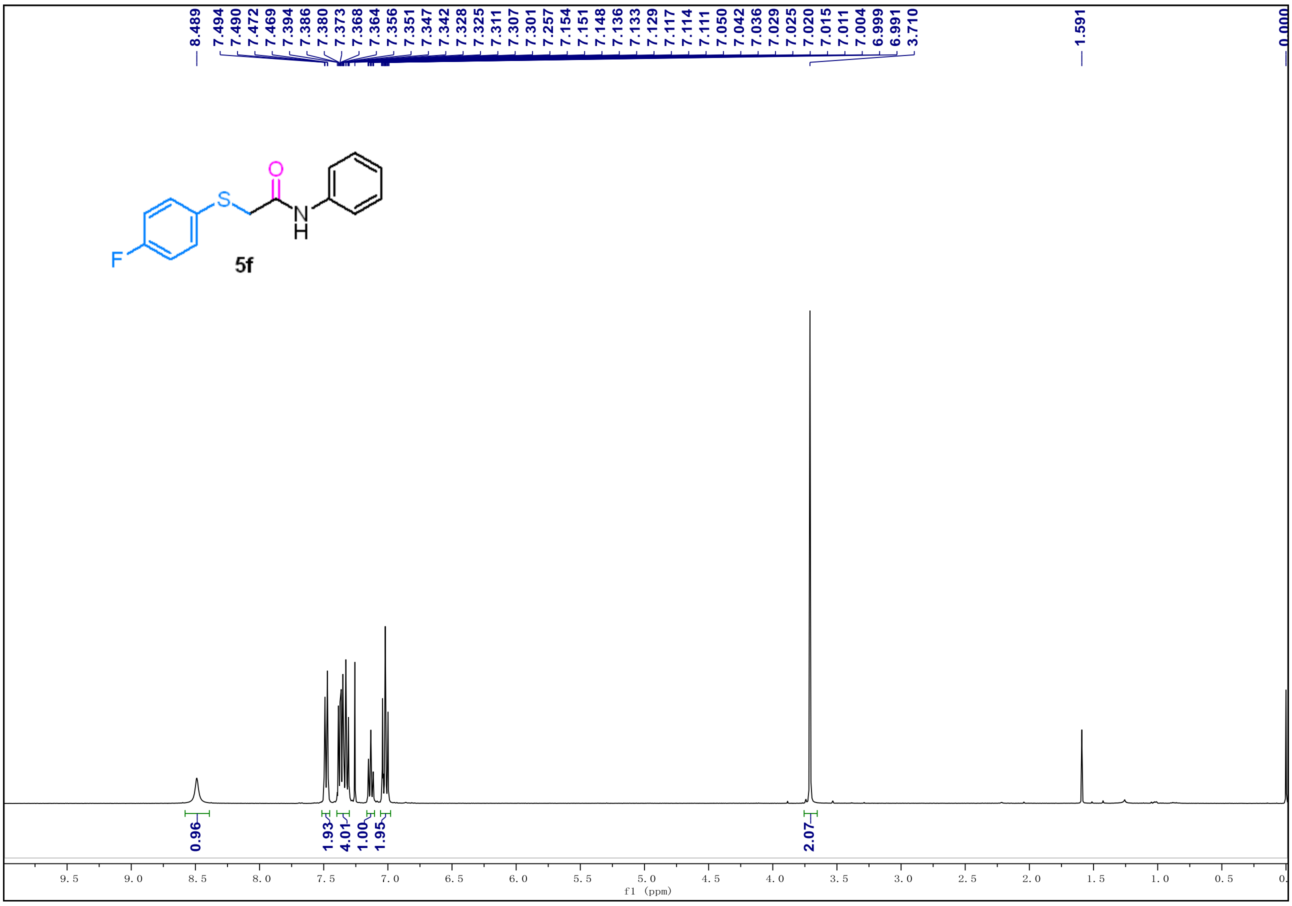

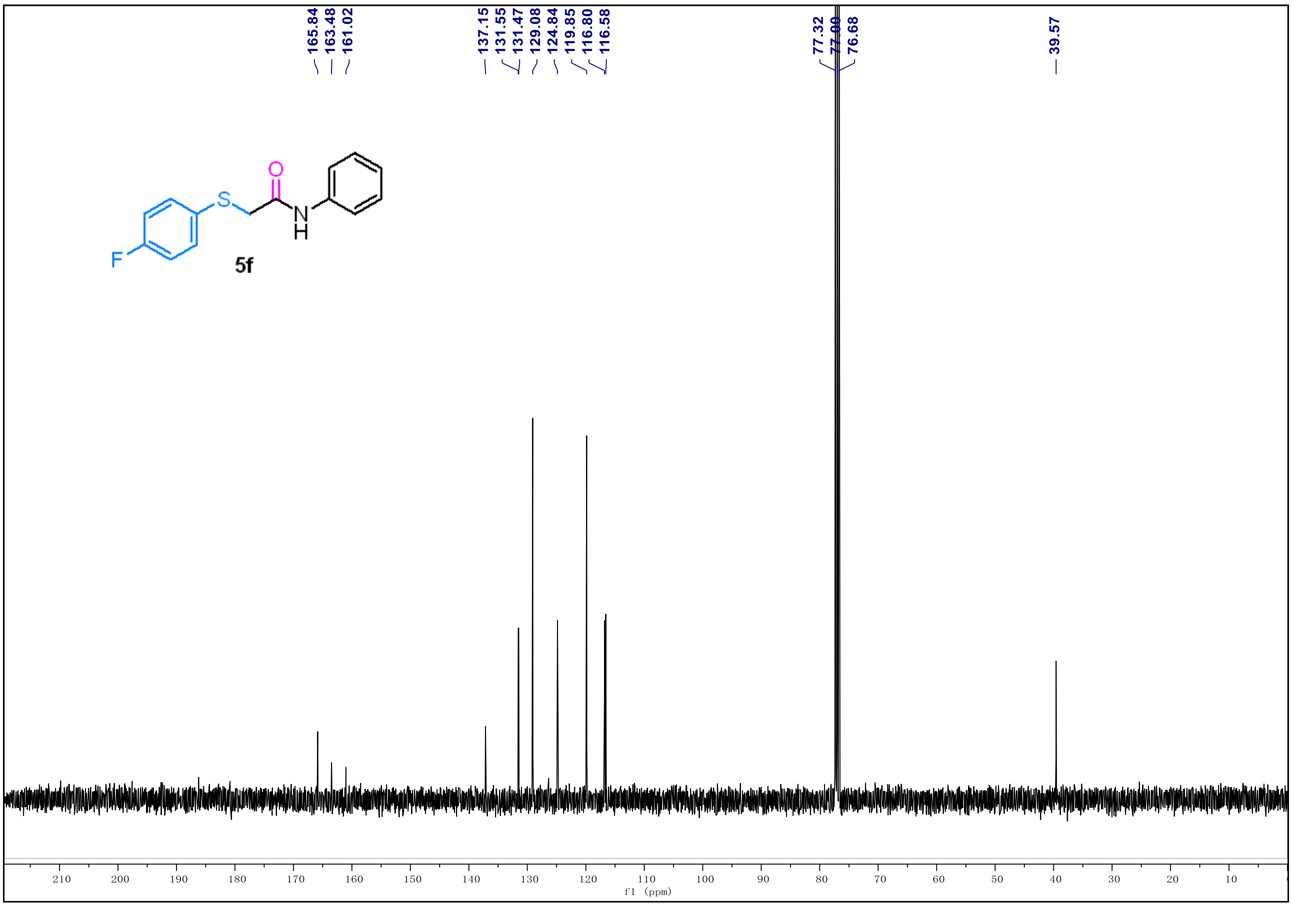

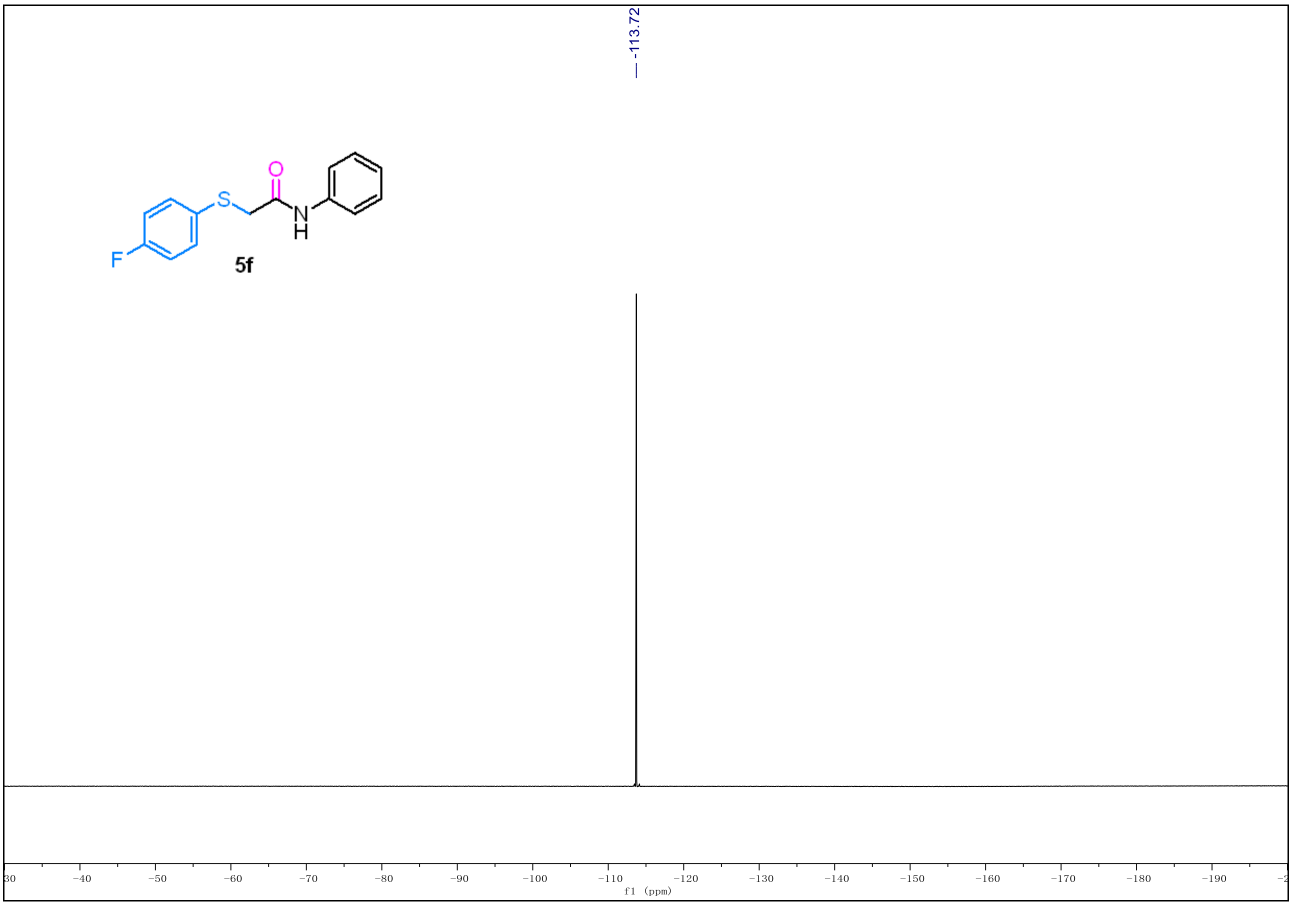
-113.72
5f
f1 (ppm)

^{1}H NMR (400 MHz, CDCl3) and ^{13}C NMR (101 MHz, CDCl3) spectrum of 5g

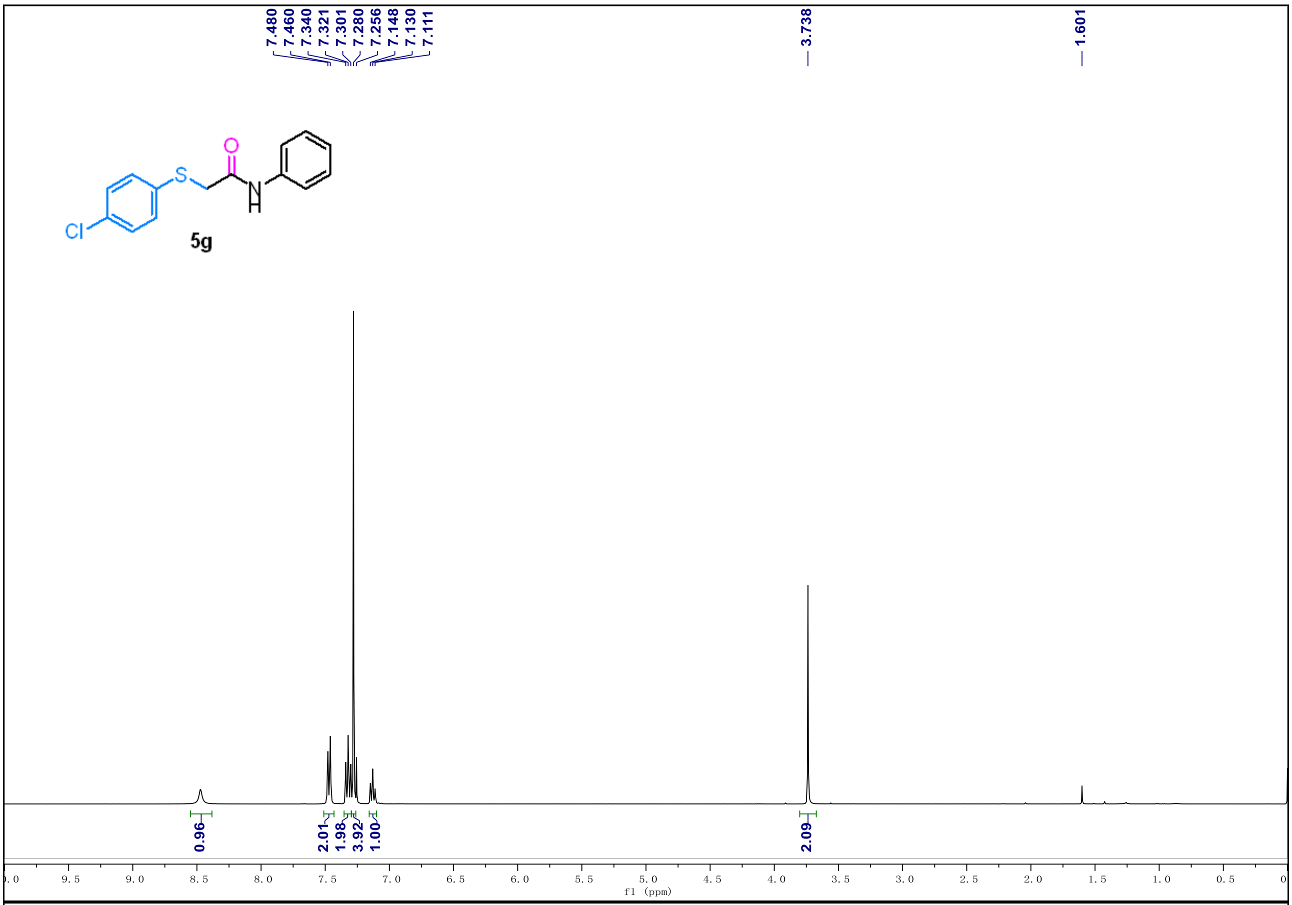

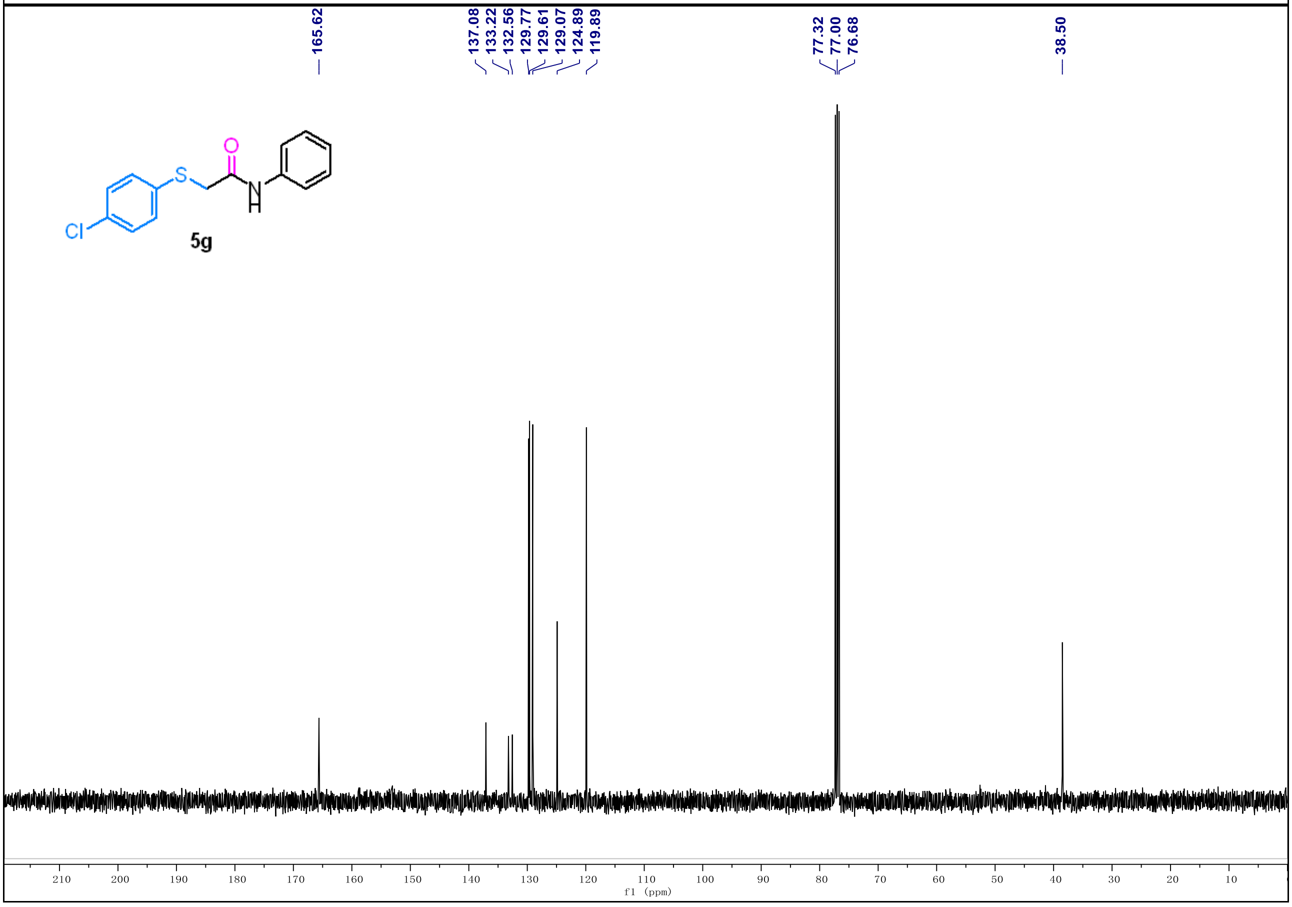

^{1}H NMR (400 MHz, $CDCl_3$) and ^{13}C NMR (101 MHz, $CDCl_3$) spectrum of 5h

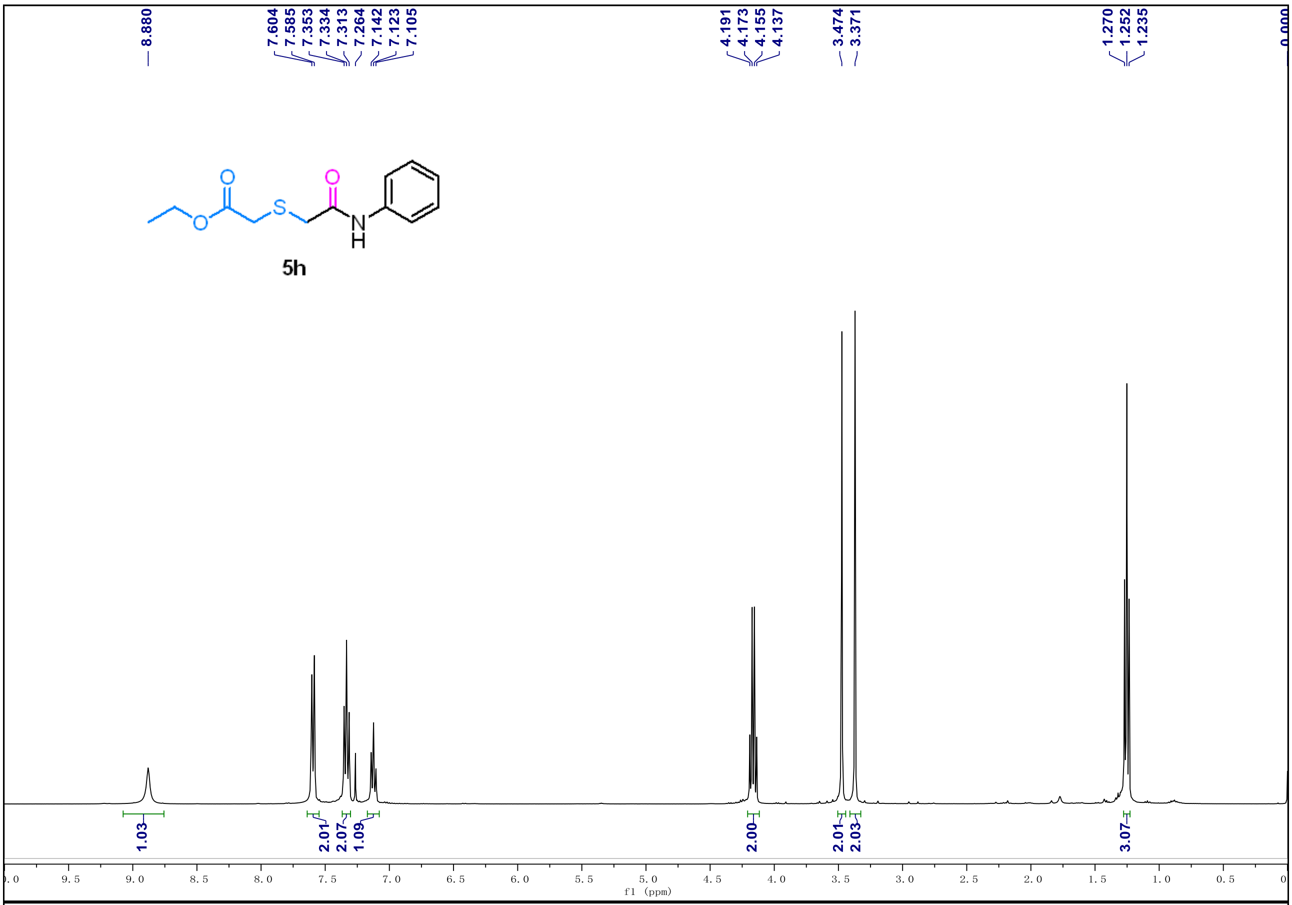

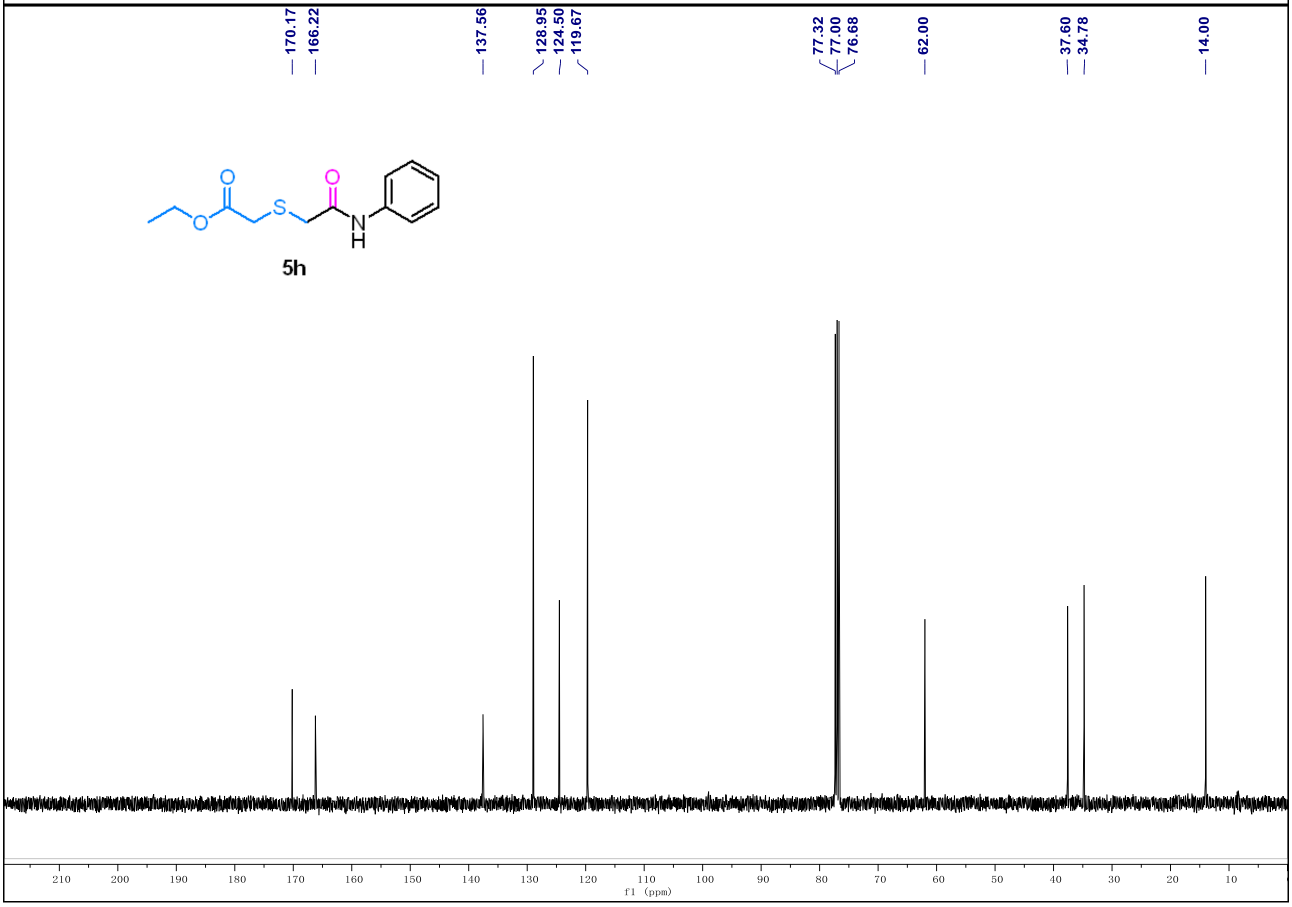

^{1}H NMR (400 MHz, $CDCl_3$) and ^{13}C NMR (101 MHz, $CDCl_3$) spectrum of 5i

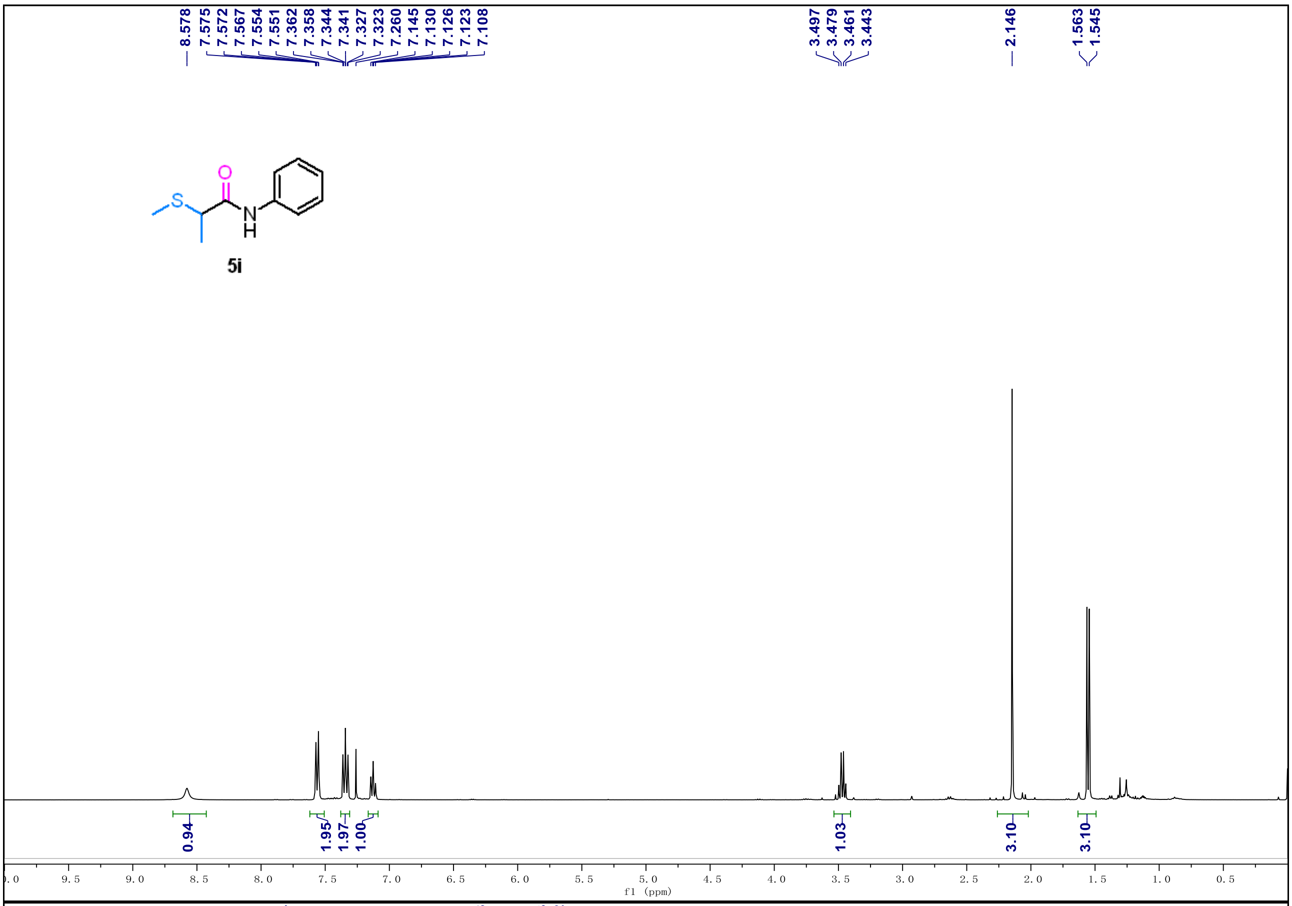

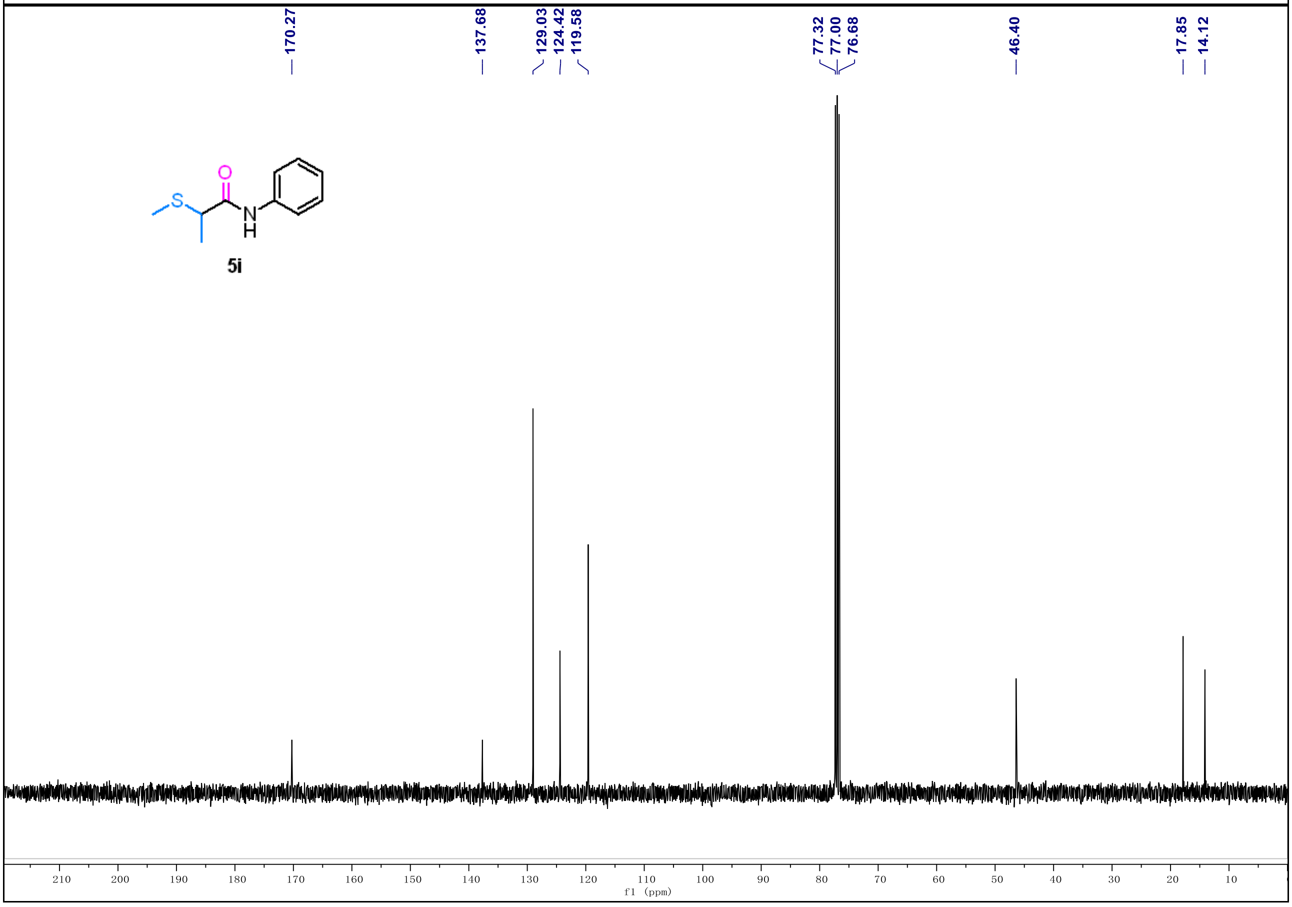

^{1}H NMR (400 MHz, $CDCl_3$) and ^{13}C NMR (101 MHz, $CDCl_3$) spectrum of 5i'

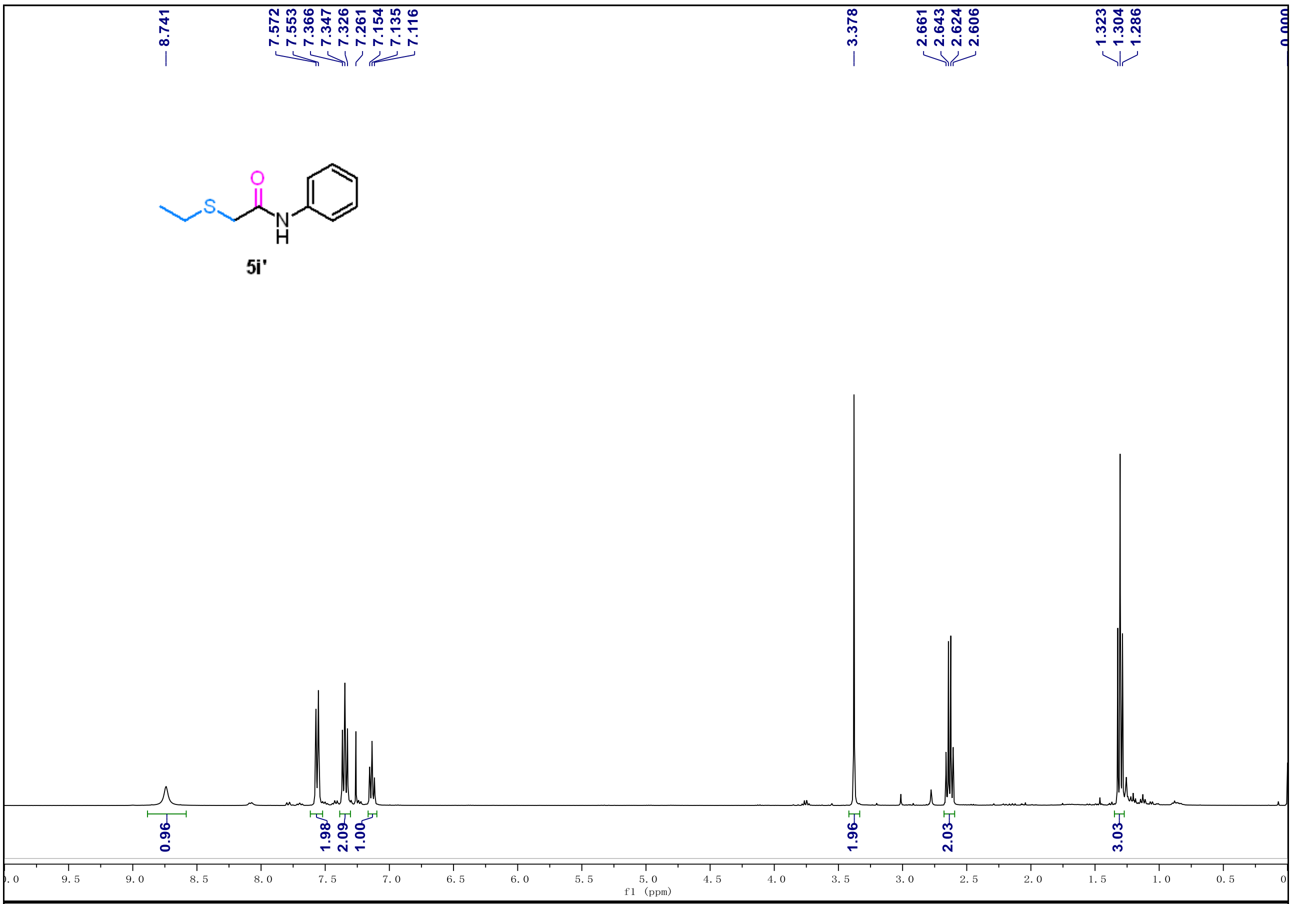

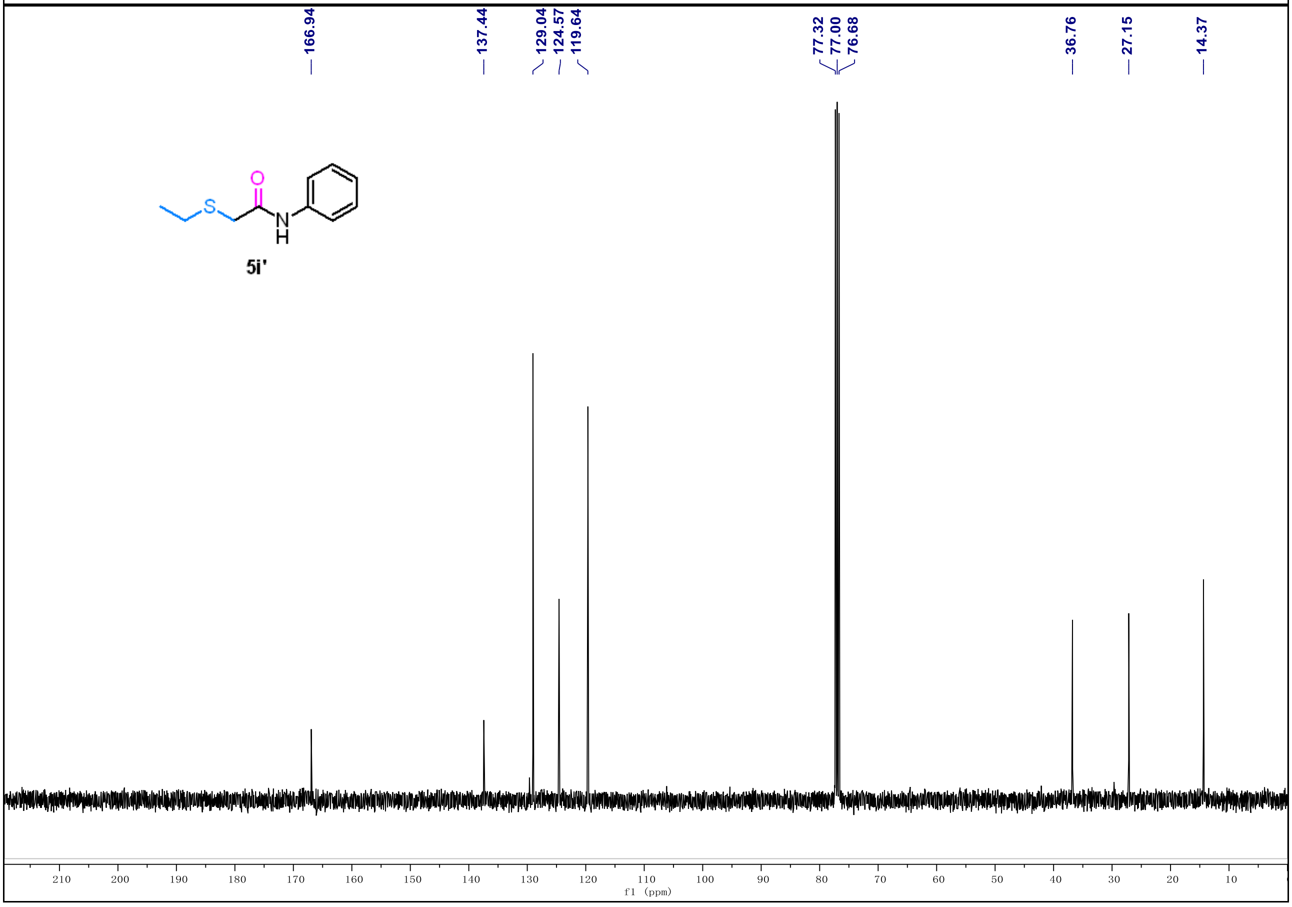

^{1}H NMR (400 MHz, CDCl$_3$) and ^{13}C NMR (101 MHz, CDCl$_3$) spectrum of 6

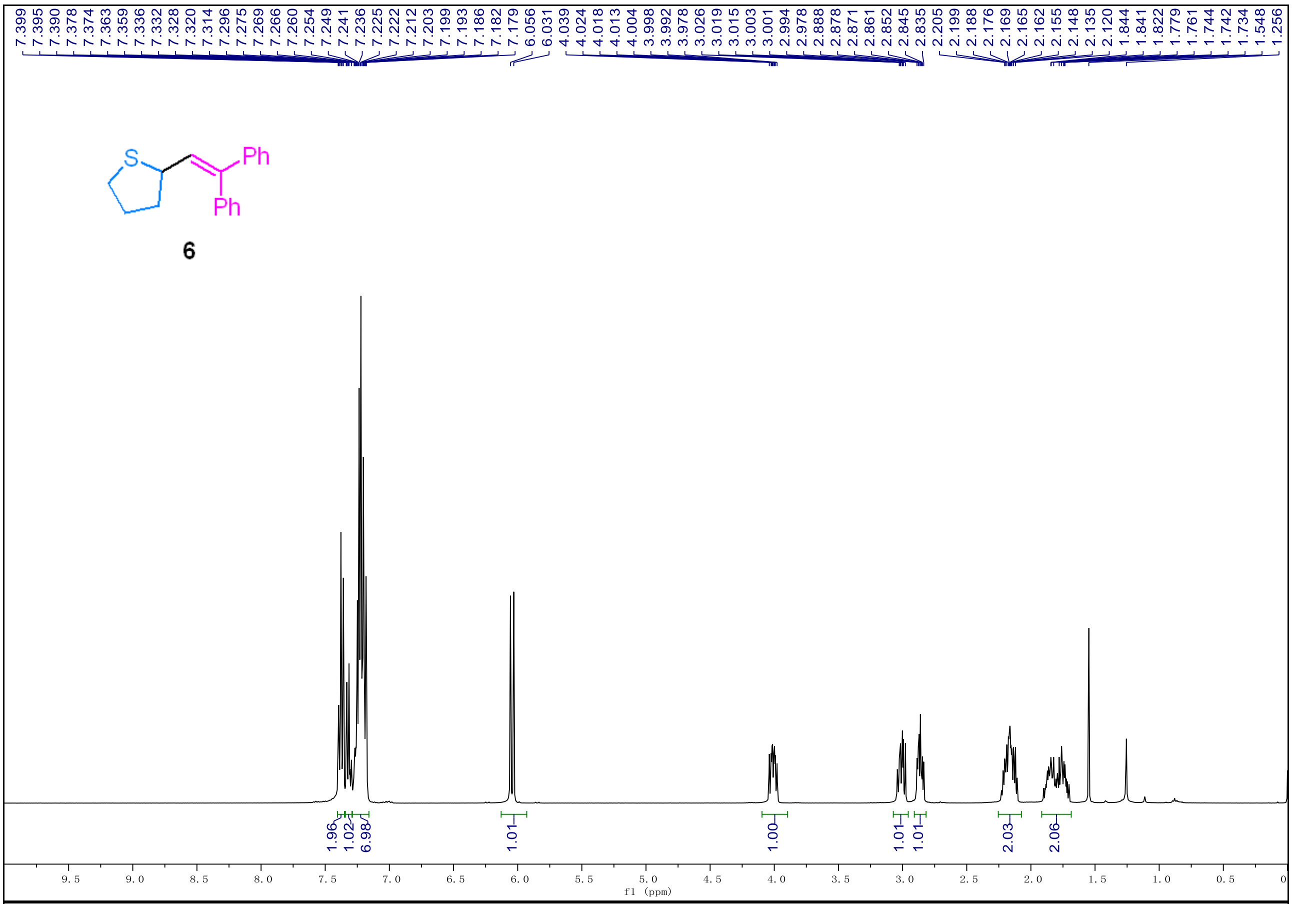

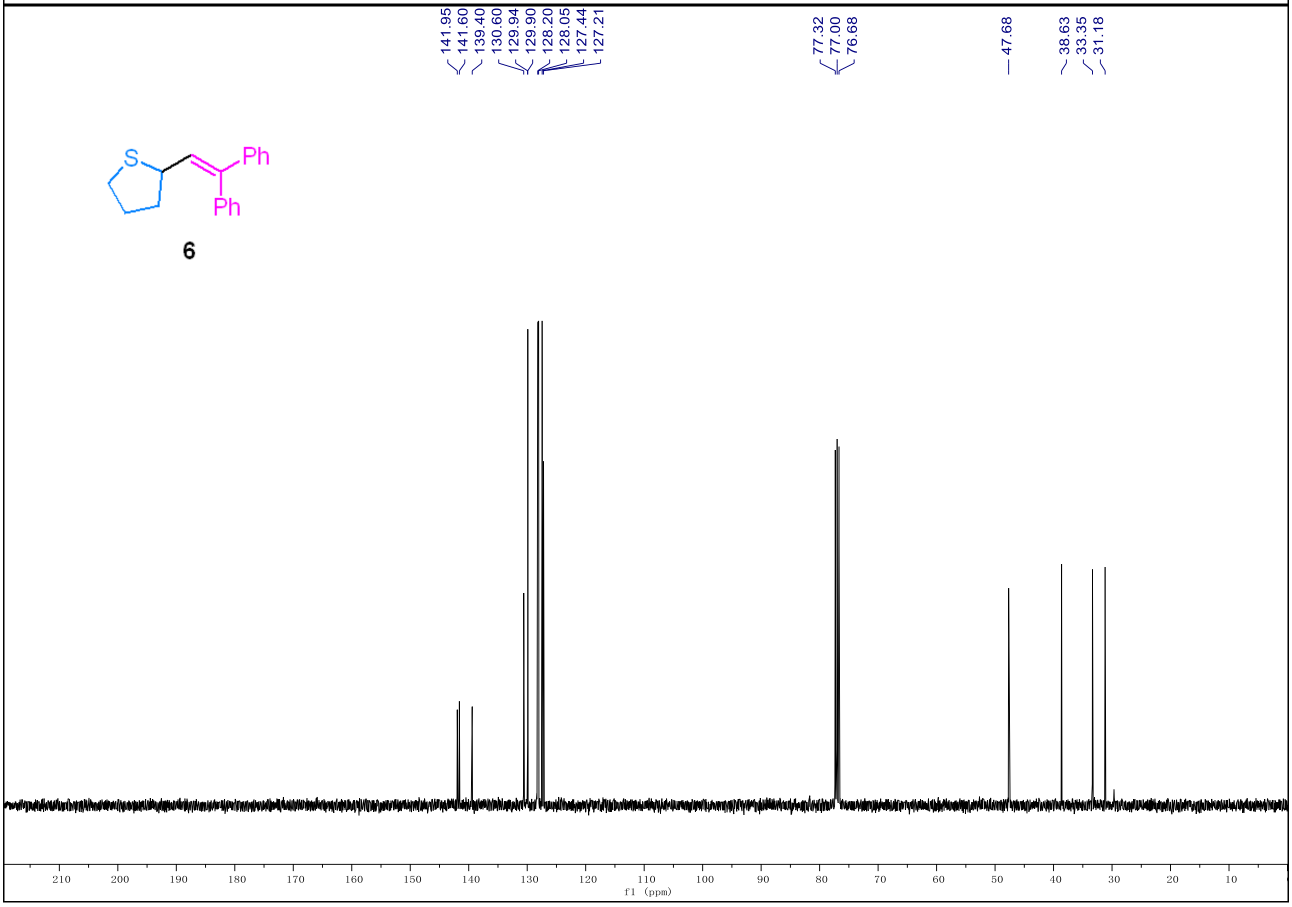

^{1}H NMR (700 MHz, $CDCl_3$) and ^{13}C NMR (175 MHz, $CDCl_3$) spectrum of 8

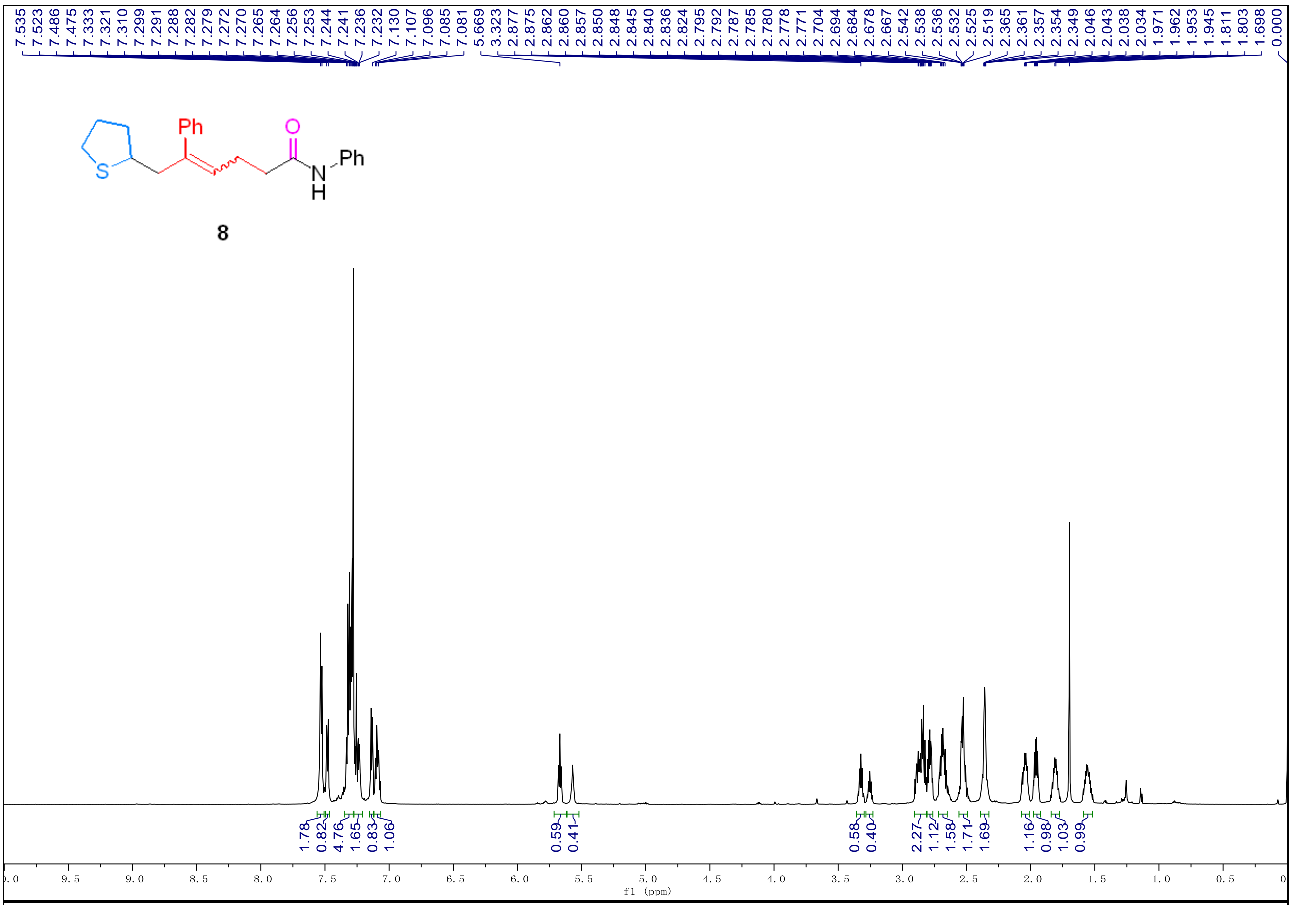

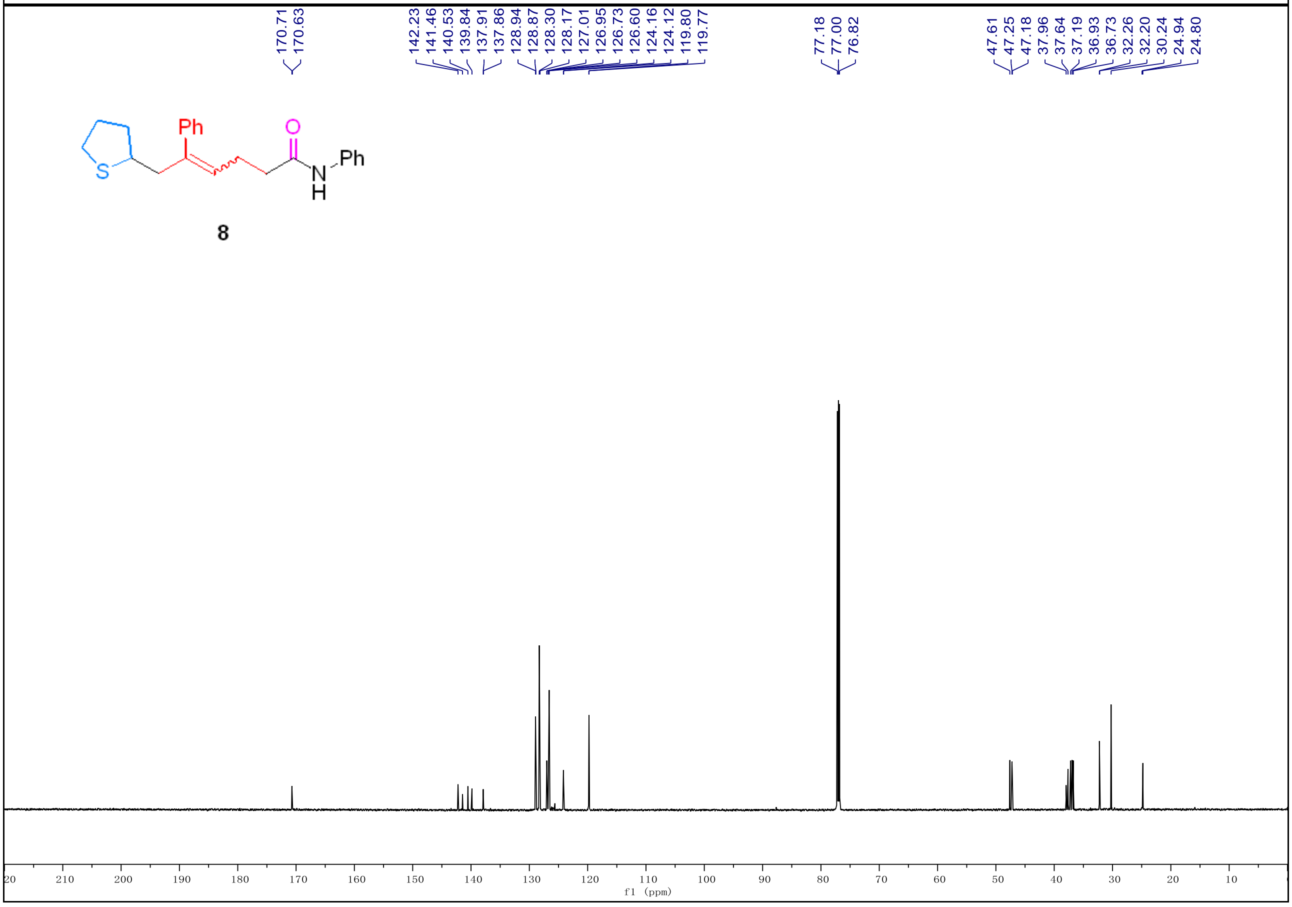

Supplement: SC-016-D5SC06696D-s001 [file SC-016-D5SC06696D-s001.pdf]
